# Ultra-strong bio-glue from genetically engineered polypeptides

**DOI:** 10.1038/s41467-021-23117-9

**Published:** 2021-06-14

**Authors:** Chao Ma, Jing Sun, Bo Li, Yang Feng, Yao Sun, Li Xiang, Baiheng Wu, Lingling Xiao, Baimei Liu, Vladislav S. Petrovskii, Bin Liu, Jinrui Zhang, Zili Wang, Hongyan Li, Lei Zhang, Jingjing Li, Fan Wang, Robert Gӧstl, Igor I. Potemkin, Dong Chen, Hongbo Zeng, Hongjie Zhang, Kai Liu, Andreas Herrmann

**Affiliations:** 1 Department of Chemistry, Tsinghua University, Beijing, China; 2 Zernike Institute for Advanced Materials, University of Groningen, Groningen, The Netherlands; 3 School of Engineering and Applied Sciences, Harvard University, Cambridge, MA USA; 4 State Key Laboratory of Rare Earth Resource Utilization, Changchun Institute of Applied Chemistry, Chinese Academy of Sciences, Changchun, China; 5 Department of Chemical and Materials Engineering, University of Alberta, Edmonton, Alberta Canada; 6 Institute of Process Equipment, College of energy engineering, Zhejiang University, Hangzhou, China; 7 Physics Department, Lomonosov Moscow State University, Moscow, Russian Federation; 8 N. N. Semenov Institute of Chemical Physics, Russian Academy of Sciences, Moscow, Russian Federation; 9 DWI - Leibniz Institute for Interactive Materials, Aachen, Germany; 10 National Research South Ural State University, Chelyabinsk, Russian Federation; 11 Institute of Technical and Macromolecular Chemistry, RWTH Aachen University, Aachen, Germany

**Keywords:** Biomedical materials, Biomedical materials, Biomedical materials

## Abstract

The development of biomedical glues is an important, yet challenging task as seemingly mutually exclusive properties need to be combined in one material, i.e. strong adhesion and adaption to remodeling processes in healing tissue. Here, we report a biocompatible and biodegradable protein-based adhesive with high adhesion strengths. The maximum strength reaches 16.5 ± 2.2 MPa on hard substrates, which is comparable to that of commercial cyanoacrylate superglue and higher than other protein-based adhesives by at least one order of magnitude. Moreover, the strong adhesion on soft tissues qualifies the adhesive as biomedical glue outperforming some commercial products. Robust mechanical properties are realized without covalent bond formation during the adhesion process. A complex consisting of cationic supercharged polypeptides and anionic aromatic surfactants with lysine to surfactant molar ratio of 1:0.9 is driven by multiple supramolecular interactions enabling such strong adhesion. We demonstrate the glue’s robust performance in vitro and in vivo for cosmetic and hemostasis applications and accelerated wound healing by comparison to surgical wound closures.

## Abstract (web summary)

Biomedical glues often face a challenge in providing strong adhesion and providing remodelling capabilities. Here the authors report on the development of a biocompatible and biodegradable protein-based coacervate adhesive and demonstrate application in haemostasis and wound healing using pig models.

## Introduction

Strong adhesives in both dry and wet conditions play an important role in many technical^1–3^ and clinical applications^4–6^. Traditionally, polymer adhesives develop high adhesion strengths through coating asperities and retarding the fracture of adhesive joints. This is achieved by in situ polymerization or crosslinking of reactive monomers that form permanent, non-adaptive covalent bonds or networks^7,8^. Recently, systems based on supramolecular interfacial bond formation, such as catechol or host-guest motifs, were introduced^9–11^. However, they failed to deliver strong adhesion strengths under ambient conditions and moreover often require hard-to-prepare or irritating components.

Especially the latter should be avoided in glues employed in biomedicine. In addition, biodegradability needs to be considered for practical applications. Biodegradability can be implemented into glues by the utilization of biomacromolecules as adhesive threads since they are degraded by biochemical processes on reasonable timescales. One example for this is wound healing where proteases are upregulated in the matrix microenvironment and actively degrade both exogenous entities and native components^12^. Several elastin-based adhesives, blood-derived fibrin sealants, and other naturally derived adhesive matrices have been developed, but require laborious and time-consuming pre-treatment by heat or UV light irradiation to prime covalent bond formation risking secondary damage to the traumatized tissues^13–16^. Moreover, the existing bio-glue solutions, such as protein- or polypeptide-based structures, adhere only insufficiently to substrates (i.e., soft tissue) and/or modestly promote wound closure and natural healing processes^17–26^. Eventually, an ideal adhesive for regenerative medicine should combine biocompatibility and -degradability with high adhesive strength, yet still being adaptive and flexible to respond to remodeling tissues where motile cells dynamically change their positional and structural order^27,28^.

To accommodate these challenges, we here present the design of a family of supercharged polypeptide-based adhesives for in vivo tissue engineering applications. These glues were formed by electrostatic complexation of cationic polypeptides and anionic aromatic surfactants avoiding covalent bond formation during the gluing process. A well-balanced combination of non-covalent bonds gives rise to ultra-high fracture strengths surpassing known protein-based adhesives by one order of magnitude. As a result, external skin wounds and internal organ defects were sealed fast and at the same time wound healing was accelerated.

## Results and discussion

**Fig. 1:**
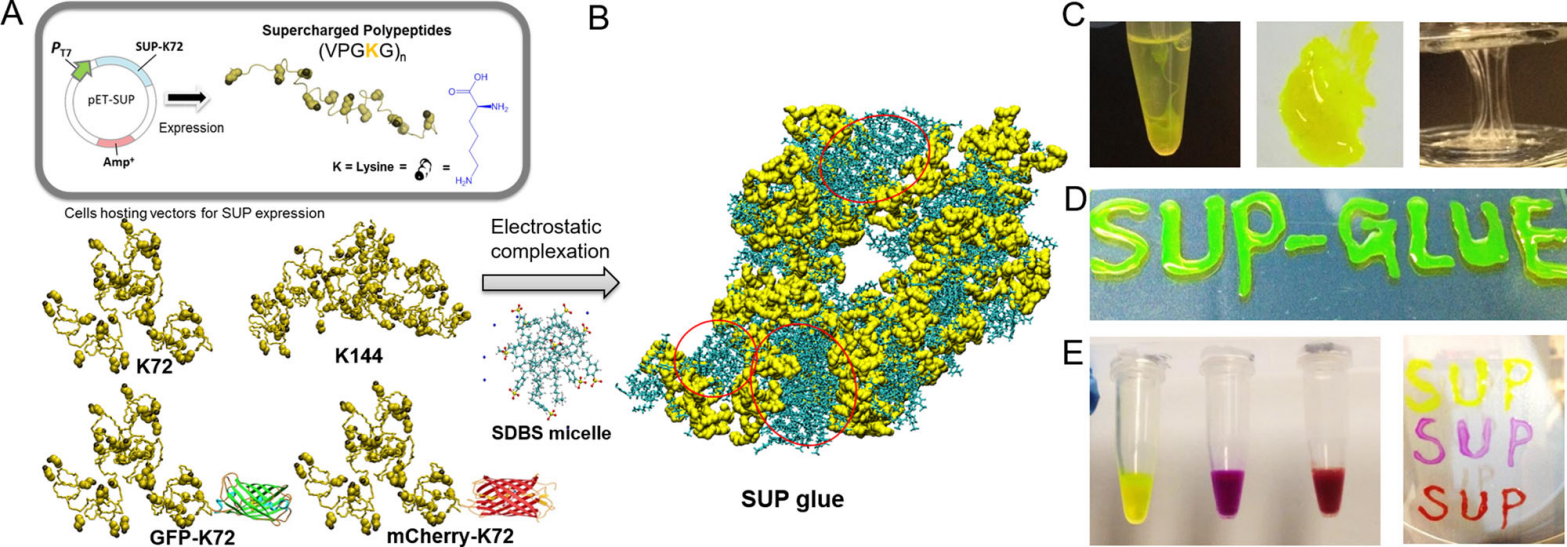
Fabrication and investigation of the SUP-SDBS glue system. **A** Schematic illustration of cationic supercharged polypeptide (SUP) expression through genetically engineered *E. coli*. The polypeptide backbones are shown in a random coil conformation. Lysine is presented in a space-filling model in yellow and the charged amino-group in gray. A series of SUPs with different chain lengths (K18, K36, K72, K108, and K144) and SUP fusions with GFP and mCherry were produced. **B** The glue was prepared via electrostatic complexation of SUP and SDBS micelles. An all-atomistic simulated K18-SDBS complex is shown. In this model, K18 is shown in yellow, SDBS in cyan, and some of SDBS micelle domains are encircled in red. **C** For the fabrication, a solution of SUP (here GFP-K72) was mixed with a solution of SDBS micelles leading to liquid-liquid phase separation and the formation of a coacervate. After centrifugation, the supernatant was removed (left) and the coacervate consisting of SUP-SDBS complex was collected by a pipette and applied to a glass surface (middle and right). **D** Fluorescent SUP glues show robust adhesion behavior when applied on glass surfaces and are deformable. **E** GFP-SUP, mCherry-SUP, and a mixture of both with different emission colors were used to prepare adhesives.

Cationic supercharged polypeptides (SUPs) are inspired by natural elastin and were recombinantly expressed in *Escherichia coli*^29,30^. The high net charge of SUPs is encoded in the pentapeptide repeat unit (VPGKG)_n_ in which the fourth-position valine is substituted with a lysine residue (K) (Fig. 1A) that is protonated under physiological conditions. A series of SUPs with different numbers of repeating units, and thus chain lengths, including K18, K36, K72, K108, and K144, were produced. The digit denotes the number of positive charges along the polypeptide backbone (Supplementary Fig. 1–3 and Tables 1–2). In addition, green and red fluorescent proteins (GFP and mCherry) were fused to the unfolded cationic SUPs to demonstrate their easy functionalization with folded proteins and for facile tracing.

Subsequently, the anionic surfactant sodium dodecylbenzene sulfonate (SDBS), which is an FDA-approved surfactant for cosmetics^31^, was complexed with the cationic SUPs to form the adhesive. Therefore, SUPs and SDBS micelles were mixed in aqueous solution in a 1:1 molar ratio of lysine unit to surfactant (Fig. 1B). As a result, the solution became turbid and after centrifugation a protein- and surfactant-rich liquid was obtained at the bottom of the tube (Fig. 1C). This behavior is termed complex coacervation and represents a liquid-liquid phase-separation process, which widely exists in nature, e.g. in mussel or sandcastle worm protein adhesives and in artificial biopolymeric adhesion systems^2,17,24^. Specifically, the coacervate here can be regarded as proteinaceous polyelectrolytes (SUPs) in an aqueous solution combined with counterions (SDBS), yielding phase-separated aggregates. The coacervate formation of the system was further investigated by determining its phase diagrams in regard to the concentration of the components, polypeptide to surfactant ratio, pH, and ionic strength (Supplementary Fig. 4). It suggests that the occurrence of a coacervate in the system is a characteristic phase-separation phenomenon. Particularly under the condition of ~150 mm physiological saline, the complex indeed does not swell.

After separation of the supernatant, the SUP-SDBS coacervates were viscous but plastically deformable (Fig. 1C–E). It is worth noting that SDBS molecules are present as micelles during the process of complexation (Fig. 1B and Supplementary Fig. 5). A representative and quantitative component determination of the SUP-SDBS complexes was carried out by proton nuclear magnetic resonance (^1^H-NMR) spectroscopy. For the K18-SDBS complex, a stoichiometry of K18:SDBS of 1:16 was measured, equaling a ca. 90% occupation of the positive lysine residues by surfactant molecules (Supplementary Fig. 5). Thermogravimetric analyses (TGA) showed that the SUP-SDBS complexes exhibited a water content of ~42% (w/w) (Supplementary Fig. 7). Besides pristine SUP chains, fusions of SUPs with proteins of different absorption and emission colors, i.e., GFP and mCherry, were converted into adhesives by complexation with SDBS, analogously to the pristine peptide chains (Fig. 1E).

**Fig. 2:**
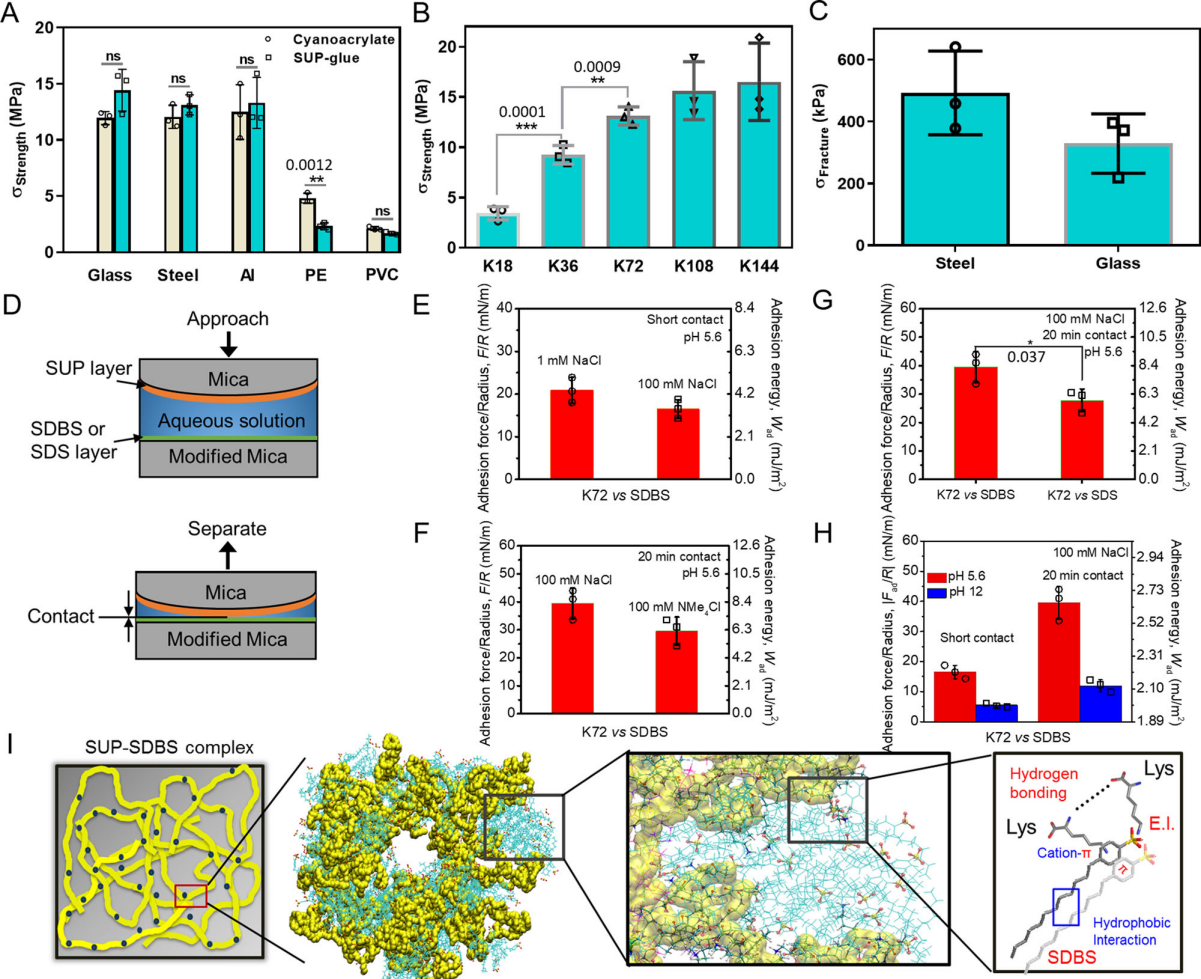
Bulk adhesion behavior of the SUP glues. **A** Illustration of lap shear testing for the K72-SDBS glue and fracture strengths in comparison to commercial cyanoacrylate-based glue on glass, steel, aluminum, polyethylene (PE), and polyvinyl chloride (PVC). ns, no significant difference. **p < 0.01. **B** Correlation of bulk adhesion strength measured on steel substrate with SUP molar masses starting from K18 and ranging to K144. **p < 0.01; ***p < 0.001. **C** SUP glue lap shear testing in an aqueous environment. Two sets of measurements were performed on steel and glass, respectively. **D** Schematic of surface force apparatus (SFA) measurement. Two opposing curved mica surfaces with radius R in crossed-cylinder configuration coated with a K72 layer and an SDBS (or a sodium dodecyl sulfate (SDS)) layer, respectively, were first approached to each other until they come into contact, and were then separated to measure the adhesion force (*F*_ad_). **E** Impact of aqueous conditions on the adhesion between K72 and SDBS layers as revealed by SFA measurements. 1 mm NaCl vs. 100 mm NaCl (pH 5.6 and short contact); **F** 100 mm NaCl vs. 100 mm NMe_4_Cl (pH 5.6 and 20 min contact); **G** Adhesion comparison between K72 and SDBS layers, and between K72 and SDS layers in 100 mm NaCl at pH 5.6. *p < 0.05. **H** pH 5.6 vs. pH 12 (100 mm NaCl and 20 min contact). The short contact refers to a contact time of 1 min. **I** Proposed molecular mechanism for the strongly adhesive SUP-SDBS complexes. The simulated structure of the K18-SDBS complex is presented in yellow and cyan, respectively. Different interactions govern the adhesive and cohesive strength including electrostatic interactions (E.I.), cation-π interactions, van der Waals forces, hydrogen bonds, hydrophobic interactions, and other aromatic interactions. All presented data are mean values ± SD from the mean from *N* = 3 independent measurements on independent samples. All *p*-values were calculated using two-sided Student’s *t*-test.

After fabrication, the bulk adhesion strengths of the SUP glues were investigated by lap shear testing (Fig. 2A and Supplementary Fig. 10). K72-SDBS and commercial cyanoacrylate glue (as a comparison) were applied on various substrates including glass, steel, aluminum, polyethylene (PE), and polyvinyl chloride (PVC). The K72-SDBS glue adhered strongly on high-energy surfaces (glass or metal) and exhibited fracture strengths in the range of 11.0–14.0 MPa, comparable to cyanoacrylate glue (Fig. 2A, Supplementary Figs. 10 and 11). This behavior indicates that rough, high-energy surfaces adhere to SUP glue strongly. This can be explained by the high potential of both chemical interactions and mechanical interlocking between the glue and the surface^32^. On low-energy surfaces (PVC or PE), the adhesion of SUP glue was reduced, but still as strong as cyanoacrylate, one of the strongest commercially available adhesives. Notably, the adhesive performance of SUP glue between two glass substrates was so robust that the substrate fractured before the glue failed as visualized by intact adherent regions. Furthermore, lap shear investigations involving K18-SDBS, K36-SDBS, K72-SDBS, K108-SDBS, and K144-SDBS glues indicated that the adhesion strength increased with increasing molar mass of the SUP (Fig. 2B and Supplementary Fig. 12) and by this the adhesion strengths could be tuned between 3.0 and 16.5 MPa.

Importantly, there was no difference in adhesion properties between the pristine K-SDBS groups and the fluorescent protein fusion variants indicating that the adhesive behavior can be maintained even when exogenous functional protein entities are introduced. Notably, the fracture strength of 16.5 ± 2.2 MPa of K144-SDBS was higher than any other reported protein-based adhesive and surpassed those by at least one order of magnitude^15–20^. The adhesion energies of SUP glue on different surfaces are summarized in Table S3. Comparison with chemically synthesized adhesives and genetically engineered protein-based adhesives (Supplementary Table 4) shows that SUP glue exhibits ultra-strong adhesion performance in both dry and wet conditions. To demonstrate the broad validity of the paradigm of the SUP glue system, the small molecule SDBS anionic surfactant component was replaced by salmon sperm DNA (~2000 bp) and the new adhesive consisting exclusively of biomacromolecules was termed SUP-DNA glue. The SUP-DNA showed similar adhesion properties as the SUP-SDBS system (Supplementary Fig. 14).

Adhesion not only involves rough but also wet surfaces. Hence, we investigated underwater adhesive strengths of SUP glues on two types of substrates (steel and glass) (Fig. 2C, Supplementary Fig. 14, and Supplementary Table 3). The K72-SDBS glue exhibited strong adhesion with fracture strengths of 490 and 330 kPa on steel and glass, respectively. These values are comparable to or higher than other proteinaceous underwater adhesives reported to date^19–26^. The underwater adhesion energy of SUP glue on glass and steel was 20 J m^−2^ and 51 J m^−2^, respectively, which is higher than for recently reported chemically synthesized adhesives (Supplementary Table 4)^2,6^.

Next, the effect of the molar ratio of SUP over SDBS to the adhesion properties was investigated. Since we showed that ~10% of lysine moieties within the SUP are not complexed by surfactant molecules, we hypothesized that these free lysine residues may contribute to the adhesion properties of the complexes. Therefore, we prepared SUP-SDBS complexes with different stoichiometry. When a lysine:surfactant molar ratio of 1:5 was chosen, for each lysine on average 3.3 SDBS molecules were detected by ^1^H-NMR measurements (Supplementary Fig. 16). As a result, the corresponding fracture strength decreased by about one order of magnitude suggesting that the non-complexed free lysine contributes significantly to the overall adhesion performance (Supplementary Fig. 17A). In addition, a lysine:surfactant molar ratio of 1:0.5 was adjusted (Supplementary Fig. 17B) showing similarly low adhesion as the 1:5 complex. This suggests that the ratio of lysine to SDBS needs to be properly adjusted to achieve optimal bulk adhesion properties. In addition, a systematic investigation of adhesion performance of SUP glues along with the initial ratios from 1:0.5 to 1:2 with 0.1 intervals was performed, showing peak strength in the subgroup of 1:1.0 (Supplementary Fig. 38). Complexing SUPs with a surfactant lacking the phenyl moiety (by using sodium dodecyl sulfate, SDS) does not result in any adhesive properties. These experiments might emphasize the major contribution of cation-π interactions between the free lysine residues and the phenyl ring of SDBS to the adhesive properties of the bio-based glue. The cation-π interaction is known to govern adhesive systems in nature, e.g., in mussel plaque^10^, and hence might play a pivotal role in the cohesive properties of SUP-SDBS complexes as well.

To investigate the contributions of molecular interactions involved in the complex to realize superb adhesion behavior, surface force apparatus (SFA) measurements were carried out to directly quantify the interaction forces between K72 and SDBS (Fig. 2D). It was found that when increasing NaCl concentration from 1 to 100 mm, the adhesion decreased to ~16.5 mN m^−1^ at short contact (Fig. 2E). The reduced adhesion most likely stems from the screening of the electrical double layer under high salinity conditions, shortening the Debye length from ~10 nm in 1 mm NaCl to ~1 nm in 100 mm NaCl. To evaluate the contribution of cation-π interaction in the SUP-SDBS complex, we introduced interfering cation NMe_4_^+^ to the aqueous media and then measured the adhesion between SUP and SDBS layers^33^. We compared the adhesion (*F*_ad_/*R* ~29.6 mN m^−1^) between SUP and SDBS layers in 100 mm NMe_4_Cl with that (*F*_ad_/*R* ~39.5 mN m^−1^) in 100 mm NaCl within 20 min contact (Fig. 2F, Supplementary Fig. 18B, C). The weakened adhesion in 100 mm NMe_4_Cl can be attributed to the presence of NMe_4_^+^ ions that form robust NMe_4_^+^-π pairs with SDBS, thereby reducing the cation-π interaction between the NH_3_R^+^ on the lysine residue in the SUP and the phenyl group in SDBS. To further estimate the contribution of cation-π interaction in SUP-SDBS complex, we compared the adhesion (*F*_ad_/*R* ~39.5 mN m^−1^) between SUP and SDBS layers in 100 mm NaCl with that (*F*_ad_/*R* ~27.8 mN m^−1^) of SUP and sodium dodecyl sulfate (SDS) under the same solution condition within 20 min contact (Fig. 2G and Supplementary Fig. 18B, E). The adhesion for the SDS group was significantly reduced, which can be explained by the absence of cation-π interactions between SUP and SDS due to the lack of phenyl group in SDS. Therefore, the above results demonstrate that cation-π interactions (i.e., NH_3_R^+^-π pairs) between SUP and SDBS play an important role in the adhesion process, estimated ~9.9 mN m^−1^ (i.e., 39.5–29.6 mN m^−1^), in addition to the electrostatic attraction. We further investigated the impact of pH on the adhesion, and measured the adhesion between SUP and SDBS layers in 100 mm NaCl at pH 12 (Fig. 2H and Supplementary Fig. 18D). It was found that the adhesion was significantly weakened to *F*_ad_/*R* ~5.5 and ~11.9 mN m^−1^ at pH 12 under 1 min and 20 min contact, respectively, as compared to *F*_ad_/*R* ~16.5 and ~39.5 mN m^−1^ at pH 5.6. As the pI of K72 is 11.87, the amino groups on lysine residues would deprotonate at pH 12, which could largely weaken the electrostatic and cation-π interactions between SUP and SDBS, thus reducing the adhesion. Interestingly, with increasing the contact time to 20 min, the SUP-SDBS adhesion was greatly enhanced to *F*_ad_/*R* ~11.9 mN m^−1^. It is likely that due to the molecular rearrangement under contact, the initially hidden hydrophobic moieties on one surface would expose to and interact with the opposing hydrophobic moieties on the other surface^34–36^. In view of this, the adhesion contributed from hydrophobic interactions could be roughly estimated to ~6.4 mN m^−1^. Given that the adhesion originating from electrostatic interaction and cation-π interaction was estimated to ~20.9 mN m^−1^ (in 1 mm NaCl, pH 5.6) and ~9.9 mN m^−1^, respectively, the contribution ratio of electrostatic interaction, cation-π interaction, hydrophobic interaction, and other interactions (hydrogen bonding, van der Waals, and other aromatic forces) to the formation of SUP-SDBS complex is approximately estimated as 4:2:1:1. Moreover, all-atomistic computer simulations showed the formation of disordered nanodomains of isolated SUPs bound to each other by surfactant micelles (Fig. 2F) and demonstrate the importance of cation-π and other supramolecular interactions for the strong adhesive properties of the complex (Supplementary Movie 1, and Supplementary Fig. 19 and 20). Furthermore, by systematically investigating the water contents in K72-SDBS complex, we conclude that the presence of a certain amount of water in the complex can strengthen and maintain intermolecular interactions in the system, allowing the lysine residues to be positively charged and available for electrostatic attraction and cation-π interaction with SDBS, as well as for maintaining the micellar structures of SDBS (Supplementary Fig. 13).

Therefore, the robust cohesion during SUP-SDBS complexation is mainly realized through the synergy of multiple interactions including electrostatic interactions, cation-π interactions, hydrophobic interactions, hydrogen bonding, and van der Waals interactions. When applying this SUP-SDBS glue to different substrate surfaces, the flowability of the SUP-SDBS coacervate enables the intimate contact between the SUP glue and substrates; meanwhile, the surface molecules and functional groups of the coacervate possess good flexibility to rearrange and interact with opposing substrate surfaces via a synergy of multiple interactions such as electrostatic interactions, cation-π interactions, hydrophobic interactions, hydrogen bonds and van der Waals interactions (depending on the substrate surface properties), thus leading to the strong glue-substrate adhesion as we have measured in bulk adhesion tests. Such strong cohesion and wet adhesion mechanisms of the SUP glue are somewhat similar to marine mussel adhesives formed via the coacervation of the mussel adhesive proteins^37,38^.

The non-covalent nature of the adhesive system endows the SUP-SDBS complex with additional attractive features. Firstly, glues are biodegradable. We showed that after enzymatic treatment with elastase, the SUP component of the glue was digested as confirmed by gel electrophoresis (Supplementary Figs. 21 and 22). Moreover, SUP glue applied on substrates could to a large extent be detached from the wet substrates under sufficiently high external shear or peeling forces, which is mainly attributed to the strong and synergistic non-covalent interactions between SUP and wet substrates. Afterwards, the polypeptide material was recovered and adhesive properties were regained demonstrating the recyclability of the glue. It was found that the regenerated SUP-SDBS complex exhibited the same adhesion strength as the original, non-recycled batch (Supplementary Figs. 23 and 24).

Furthermore, the biocompatibility of the SUP glues was investigated. We cultured HeLa cells in the presence of different concentrations of K72-SDBS. Without significant deviation from the control group, the cell viability remained higher than 90% after 24 h in all experiments (Supplementary Figs. 25–27). Moreover, mice mesenchymal stem cells (D1 cells), bone marrow-derived stem cells, human dermal fibroblasts, as well as HeLa cells were embedded into the SUP glue and SUP-DNA glue matrices for 3D culturing (Supplementary Fig. 27). The viability of encapsulated cells was quantified as well, showing good biocompatibility of the SUP glue groups (Supplementary Figs. 25–27). Compared with the alginate matrix, there is no significant difference between the SUP and alginate groups in terms of cell viability.

**Fig. 3:**
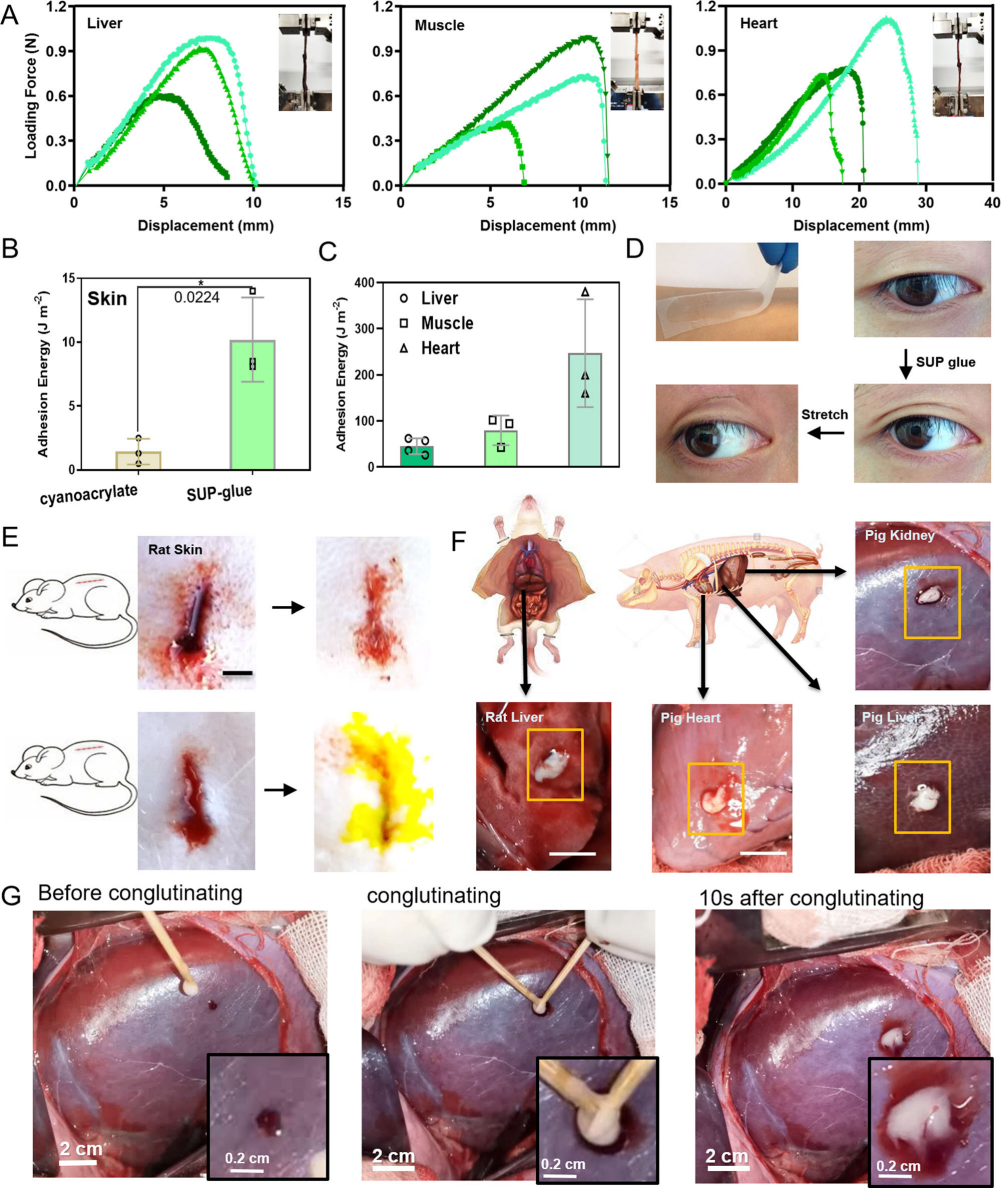
Quantification and applications of SUP glues dealing with wet tissue adhesion and wound hemostasis. **A** Quantification of pre-clinical ex vivo adhesion model with SUP-glue on porcine liver, muscle, and heart. The measurements were carried out via lap shear devices. Insets show images of the adhesion joints. Three individual curves were collected for each group of specimens. **B** Adhesion energy comparison of cyanoacrylate and SUP glue (here K72-SDBS) on pig skin. *p < 0.05. **C** Overview of SUP-glue performance in terms of adhesion energy on pig heart, liver, skin, and muscle. **D** SUP glue on parafilm pasted on the skin of the arm exhibiting strong adhesion and good conformability. SUP glue applied on a single eyelid to achieve a double eyelid effect, and reversal of the effect after stretching. Male volunteer with age of 35. **E** Bleeding wounds on rat skin treated with SUP glue. The skin wound was sealed inducing hemostasis. The second skin wound was treated with fluorescent GFP-SUP glue for tracing the glue during wound healing over nine days. **F** SUP glue worked as sealant on pig heart, kidney, liver as well as rat liver. The yellow boxes indicate the pasting position on organs. Scale bars: 10 mm (black) and 30 mm (white). **G** The demonstration of in vivo adhesion and hemostasis model on pig kidney. The bleeding wound was ceased in 10 s when SUP glue was applied. All presented data are mean values ± SD from the mean from *N* = 3 independent measurements on independent samples. All *p*-values were calculated using a two-sided Student’s *t*-test.

Motivated by the robust performance and non-toxic nature, we investigated the SUP glue’s suitability for biomedical applications ex vivo and in vivo. The quantitative evaluation of adhesion on skin or organs is intrinsically challenging due to wet conditions in combination with complex, irregular geometries and surfaces. We used SUP-SDBS complexes to glue two pieces of porcine heart, muscle, liver, and skin (Fig. 3A–C). With these samples, uniaxial extension testing was performed by recording the corresponding force-extension curves (Supplementary Movie 2). A peak force of ~1.1 N with F w^−1^ = 200 N m^−1^ and adhesion energy of 260 ± 110 J m^−2^ on porcine heart tissues was achieved, which is higher than covalently crosslinked adhesives on soft tissues reported earlier^6^. Adhesion performance of SUP glue on muscle, liver, and skin tissues was further characterized showing adhesion energies of 80 ± 26 J m^−2^, 45 ± 15 J m^−2^, and 6 ± 2 J m^−2^, respectively (Fig. 3A–C and Supplementary Fig. 8). For wet tissue, no specific adhesion strength was recorded due to the sliding nature of the soft adhesive joints when applying with stretching stress. Therefore, the quantitative descriptions of parameters, including F w^−1^ and adhesion energy, are employed using the width of the joint area. Compared to the cyanoacrylate control on the skin, the SUP glue shows significantly higher adhesion energy on this substrate (Fig. 3B). Proof-of-concept adhesive applications for cosmetics and skin healthcare were carried out on human skin and eyelids (Fig. 3D and S29).

Subsequently, the hemostatic properties of SUP glue were investigated in vivo (Fig. 3E–G). Firstly, an incision of l × h × w = 2 × 1 × 0.5 cm was performed on the back of a rat. Hereafter, K72-SDBS and GFP-K72-SDBS glues were applied to those hemorrhaging skin defects as sealants. The wounds were sealed in 10 s after the glues were applied, confirming the hemostatic properties of the SUP glues (Fig. 3E). Beyond functioning in the context of external skin defects, SUP glues exhibited tissue adhering and hemostatic properties internally on bleeding models of rat liver, pig liver, pig kidney, and pig heart (Supplementary Movie 3–8, Fig. 3F, G, and Supplementary Fig. 31). The results indicate that the in vivo wounded tissues can be fully sealed with SUP glue and the hemostasis effect was realized within 5 s. Moreover, commercial adhesive Histoacryl^®^ containing cyanoacrylate as an effective component was applied as a control, which solidified fast on the pig organs. However, the bleeding still continued when the wound was brought in contact with Histoacryl^®^, indicating the inappropriateness of this product when applied on the liver (Movie S5, Supplementary Fig. 31). Quantification of adhesion performance on organ surfaces was established in terms of hemostasis time (Supplementary Fig. 31C). It was shown that the SUP glue has superior performance on liver wounds and comparable hemostasis effects on heart and kidney wounds compared to Histoacryl^®^. These experiments demonstrate the flexibility and conformability of the SUP glue and indicate the potential of this material for tissue hemostatic applications.

**Fig. 4:**
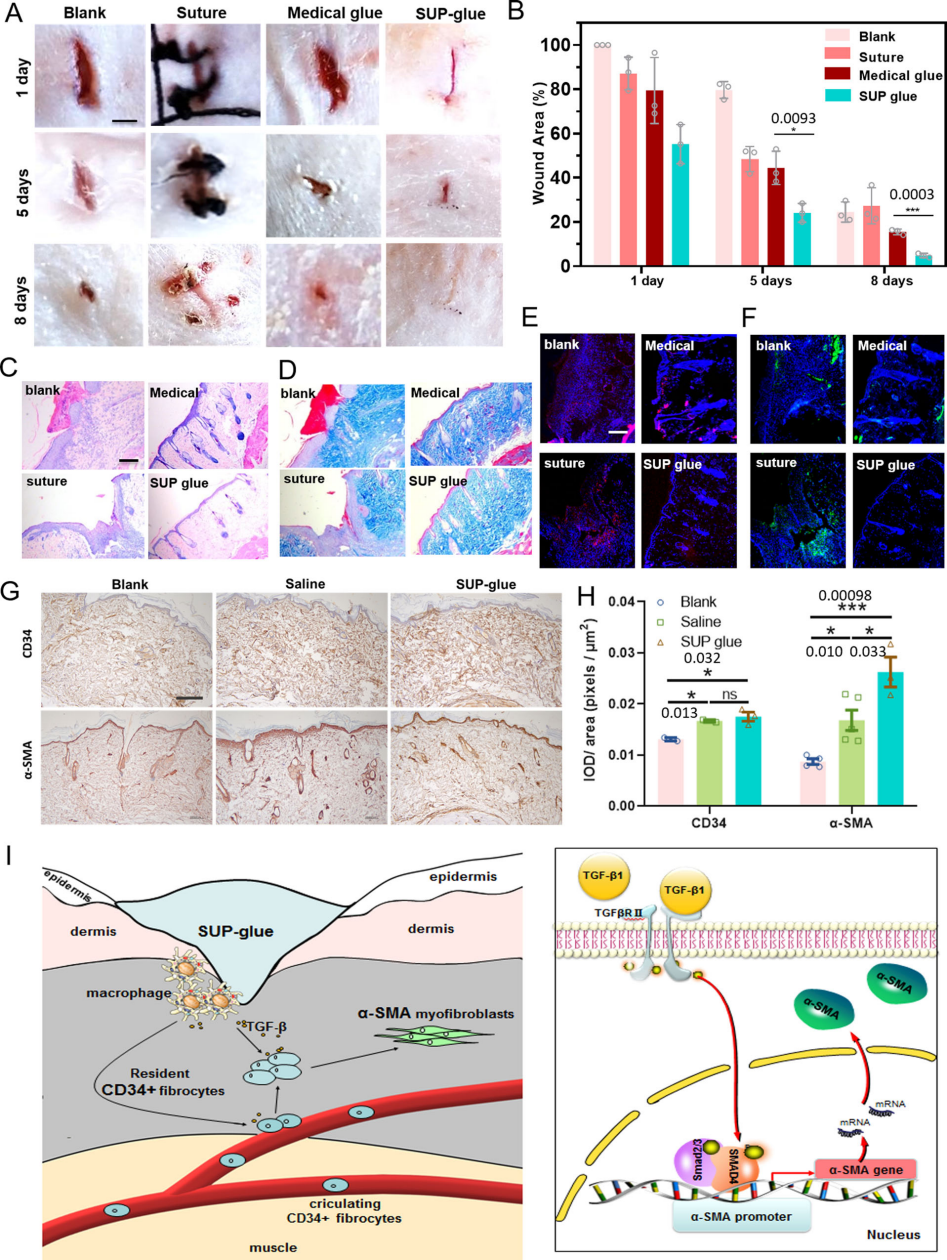
SUP glue facilitates wound sealing and tissue regeneration in vivo characterized by histological investigation of rat skin tissue in an 8 d post-wounding process. **A** Different treatments for wound healing, including blank-no treatment, suture closure, commercial medical adhesive COMPONT^®^, and SUP glue. Three times each experiment was repeated independently with similar results. Scale bar: 10 mm. **B** Quantitative analysis of SUP glue treatment over time monitoring the wound closure area. Three trials were performed for each treatment. **C** Histological investigation: H&E staining to investigate tissue regeneration. **D** Masson’s trichrome staining to show collagen recovery in the wound area. **E** Red immunofluorescent staining as an indicator of the level of IL-6. **F** Green immunofluorescent staining revealing the level of TNF-α. **C**–**E** Independent evaluation by clinical pathologists of histological results is presented in Supplementary Table 5 and Supplementary Fig. 30. **C**–**F** Three times each experiment was repeated independently with similar results. Scale bar: 100 µm. **G** Immunohistochemistry analysis on the levels of CD34 and α-SMA in the wounded areas after eight-day treatment. Anti-CD34 and anti-α-SMA antibodies were employed in these experiments, respectively. Scale bar: 200 μm. **H** Quantification of CD34 and α-SMA in the wounded areas (*p < 0.05; **p < 0.01; ***p < 0.001). **I** Proposed mechanism of the wound healing process applying SUP glue. The SUP glue accelerates the healing process by activating TGF-β/Smad pathway. All presented data are mean values ± SD from the mean from *N* = 3 independent measurements on independent samples. All *p*-values were calculated using a two-sided Student’s *t*-test.

In addition, a systematic in vivo evaluation of wound healing was performed employing a rat model with customized linear incisions and round openings (Fig. 4A and Supplementary Fig. 33). Four different animal groups for wound treatment were applied including a blank without treatment, suture closure, commercial medical adhesive COMPONT^®^, and SUP glue. In the group treated with SUP glue, the wound was tightly sealed after the SUP glue was adjusted to the incision. The healing progress was evaluated quantitatively over 8 d (Fig. 4B). After 5 d, a significant increase of repaired wound area was detected for the SUP glue compared to the other groups, demonstrating the capacity of the SUP glue for regenerating skin. On day 8, the scar was almost invisible for the rats treated with SUP glue (4% wound area left), outperforming the commercial medical adhesive. A similar trend was observed in a study involving injury dressing of round wounds (Supplementary Fig. 33). From these experiments, one can conclude that the SUP glue is actively facilitating hemostasis and accelerating the healing of wounds of different geometries. In stark contrast to suture closure and commercial chemical adhesives, the SUP glue with its biodegradable nature and supramolecular bonding might accustom well to dynamics of matrix and tissue, which might explain accelerated healing and regeneration of skin defects^27,28^.

Histological analyses applying hematoxylin and eosin (H&E) as well as Masson’s trichrome staining were utilized to analyze the regeneration of healed skin tissue. H&E staining showed the formation of new blood vessels and abundant follicle and sebaceous glands in the group treated with SUP glue while the recovery of control group tissues was inferior (Fig. 4C and Supplementary Fig. 34). Masson’s trichrome staining revealed that there was more collagen deposition when treated with SUP glue than for the other treatment groups (Fig. 4D and Supplementary Fig. 35). The H&E and Masson’s trichrome staining results of the four groups were judged by double-blind scoring in regard to the healing performance on the 8th day according to the degree of skin repair, inflammatory cell infiltration, newborn capillary formation, and collagen levels around the wound. The evaluation indicates that the SUP-glue showed superior performance among all test groups (Supplementary Table 6 and Supplementary Fig. 32). On top of blood leakage, inflammation is another fatal consequence of severe wounds. Therefore, the efficacy of SUP glue to prevent injury-associated inflammation was assessed via immunofluorescence analysis. In this regard, pro-inflammatory cytokines including interleukin-6 (IL-6) and tumor necrosis factor-α (TNF-α) were measured. IL-6 accelerates scar formation via stimulating keratinocyte migration and proliferation, and mediating fibroblast proliferation by platelet-derived growth factor^39,40^. In our study, IL-6 in the wound tissue was suppressed after administration of SUP glue, compared with controls, indicating the potential function of SUP glue, i.e., prevention of keloid and hypertrophic scar occurrence (Fig. 4E and Supplementary Fig. 36). Moreover, for the same control groups, green fluorescence was recorded suggesting high levels of secreted TNF-α (Fig. 4F and Supplementary Fig. 37). In stark contrast, the wounds treated with SUP glue did not show these signs of inflammation. Interestingly, when GFP-SUP glue was used for the experiments, no GFP signal was detected in wound areas, which is a strong indication for the in vivo biodegradability of the glue (Supplementary Fig. 37). Therefore, the degradation of the SUP backbone into body’s own amino acid building blocks might be important for the comparatively low immunogenicity and remodeling of matrix and tissue. This is an attractive feature of the SUP glue for accelerating wound healing and especially promising for future surgical applications. Furthermore, we investigated whether the contribution of SUP glue on the process of wound healing involves certain signaling pathways. In the TGF-β/Smad pathway, resident CD34 positive (cluster of differentiation 34, CD34+) fibrocytes are phenotypically metamorphosed to SMA positive (α-smooth muscle actin, SMA+) myofibroblasts under the stimulation of TGF-β1. Then SMA assists to remodel the extracellular matrix (ECM) and promotes the contraction and healing of wound tissue^40,41^. The immunohistochemistry staining tests showed that the level of CD34 around the wound was significantly upregulated in the SUP glue group particularly compared with the control group after eight-days healing implying an increased influx of circulating CD34+ fibrocytes to wound areas and intensive production of α-SMA (Fig. 4G, H). Thus, we suggest that the SUP glue might interact with macrophages or other antigen-presenting cells, promoting the upregulation of inflammatory cytokines and chemokines around wound tissue and thus facilitating the wound healing process (Fig. 4I).

To summarize, we here demonstrated that robust adhesive strength, comparable to cyanoacrylate superglue, can be achieved in the absence of polymerization or crosslinking processes involving the formation of covalent bonds. Instead, an intricate set of non-covalent interactions establishes strong adhesion and cohesion in the dry and wet states. Adhesive threads were realized on various hard substrates and soft tissues. The adhesive performance is more than ten times higher than for all other bio-inspired protein-based adhesives reported to date. The supramolecular buildup of cationic SUPs complexed electrostatically to aromatic surfactants endows the SUP glue with ultra-strong adhesive and biodegradable properties. This unique set of features renders the material perfectly suited for cosmetic skincare applications and as an in vivo bio-glue enabling tissue regeneration after surgical interventions. The robust in vivo performance was highlighted by wound-healing studies characterized by fast hemostasis, avoidance of an inflammatory response, and accelerated healing.

## Methods

### Materials

SDBS and other chemicals were obtained from Sigma-Aldrich (Netherlands and China). Illustration figures of chemical structures in the manuscript were plotted via ChemDraw software (version 11). Double-stranded salmon sperm DNA was purchased from Thermo Fisher (Waltham, MA). The water used in this research (typically 18.2 MΩ cm at 25 °C) was from a Milli-Q ultrapure water system (Merck, Germany). All biochemicals for cloning and SUP expression, such as LB medium, salts, antibiotics as well as inducer compounds, were used as received (from Sigma-Aldrich) without any further purification. The pUC19 cloning vector, restriction enzymes, and GeneJET Plasmid Miniprep kit were purchased from Thermo Fisher Scientific (Waltham, MA). Digested DNA fragments were purified using QIAquick spin miniprep kits from QIAGEN (Valencia, CA). *E. coli* XL1-Blue competent cells for plasmid amplification were purchased from Stratagene (La Jolla, CA). Oligonucleotides for sequencing were ordered from Sigma-Aldrich (St. Louis, MO). Alpha-cyano-4-hydroxycinnamic acid and sinapinic acid was used as matrix during MALDI mass spectrometry and was purchased from Thermo Scientific (Waltham, MA). Mouse Anti-αSMA (Abcam, ab7817, lot#GR31757-1), used at 1:1500 from 1 mg mL^−1^ stock solution. Rabbit Anti-CD34 (Abcam, ab81289, lot#GR45485-1), used at 1:1500 from 1 mg mL^−1^ stock solution. Animal experiments and human skin adhesion experiments are in agreement with the guidelines of the Regional Ethics Committee for Animal and Clinical Experiments of Jilin University Institutional Animal Care and Use and the Second Hospital of Jilin University, respectively. Informed, written consent was given by human volunteers. Other solvents used in the work were of analytical grade.

### Preparation of recombinant SUP proteins

#### Cloning/gene oligomerization

The building blocks of the SUP genes were ordered from Integrated DNA Technologies (Iowa, USA). Gene and respective amino acid sequences of the monomer (K9) are shown in Supplementary Fig. 1. The SUP gene was excised from the pCloneJET vector by restriction digestion and run on a 1% agarose gel in TAE buffer (per 1 L, 108 g Tris base, 57.1 mL glacial acetic acid, 0.05 m EDTA, pH 8.0). The band containing the SUP gene was excised from the gel and purified using the QIAGEN spin-column purification kit. pUC19 was digested with *EcoR*I and *HinD*III and dephosphorylated. The vector was purified by agarose gel extraction after gel electrophoresis. The linearized pUC19 vector and the SUP-encoding gene were ligated and transformed into chemically competent DH5α cells (Stratagene, Texas, USA) according to the manufacturer’s protocol. Cells were plated and colonies were picked and grown overnight in LB medium supplemented with 100 µg mL^−1^ ampicillin, and plasmids were isolated using the GenElute Plasmid Miniprep Kit (Sigma-Aldrich, Missouri, USA). Positive clones were verified by plasmid digestion with *PflM*I and *Bgl*I and subsequent gel electrophoresis. The sequences of inserts were further verified by DNA sequencing (GATC, Konstanz, Germany). Gene oligomerization, known as recursive directional ligation (RDL), was performed as described by Chilkoti and co-workers^42^. In brief, monomer K9 was digested using *PflM*I and *Bgl*I from the parent vector as one insert. A second parent vector with K9 was cut with *PflM*I only, dephosphorylated and afterwards applied as a host plasmid. Ligation between the insert fragment and the host vector was performed in the presence of T4 ligase at 22 °C for 1 h. Positive clones were verified by plasmids miniprepand gel electrophoresis. Consequently, doubled SUP fragments (i.e., K18) were obtained. For dimerizing K18–K36, K36– K72, and K72–K144, a similar protocol was applied. The same holds true for the fabrication of K108. Therefore, K36 and K72 gene fragments were combined.

#### Expression vector construction

The expression vector pET 25b(+) was modified by cassette mutagenesis, for incorporation of a unique *Sfi*I recognition site and an affinity tag consisting of six histidine residues at the C-terminus (hence in the following sections called pET-*Sfi*I), as described before^43^. SUP fragments were obtained via restriction enzyme digest using *PflM*I and *Bgl*I from cloning vector and ligated into the expression vector pET-*Sfi*I.

For GFP-K72 and mCherry-K72 fusion proteins, the pET-*Sfi*I was further digested with *Xba*I and *Nde*I, dephosphorylated and purified using a microcentrifuge spin column kit. The GFP or mCherry genes including the ribosomal binding site were excised from the pGFP and pmCherry vectors, respectively (both vectorsare kind gifts from Prof. D. Hilvert, Federal Institute of Technology, Zurich, Switzerland) by digestion with *Xba*I and *Sac*I, and the excised gene fragments were purified by DNA extraction from agarose gel after electrophoresis. A linker sequence that connects GFP or mCherry gene and the *Sfi*I restriction site was constructed. Thus, pET-*Sfi*I, the insert containing GFP or mCherry and the linker were ligated, yielding pET-GFP-*Sfi*I or pET-mCherry-*Sfi*I. To introduce the K72 gene, pET-gfp-*Sfi*I was linearized with *Sfi*I, dephosphorylated and purified using a microcentrifuge spin column kit. The K72 gene was excised from the pUC19 vector by digestion with *PflM*I and *Bgl*I. The excised K72 gene and the linearized GFP vector were ligated, transformed into XL1-Blue cells, afterwards screened for containing the insert and verified by DNA sequencing. The construction of the vector containing the mCherry-K72 fusion was performed in an analogous manner.

#### Protein expression and purification

*E. coli* BLR (DE3) cells (Novagen) were transformed with the pET-*Sfi*I expression vectors containing the respective SUP genes. For protein production, Terrific Broth medium (for 1 L, 12 g tryptone, and 24 g yeast extract) enriched with phosphate buffer (for 1 L, 2.31 g potassium phosphate monobasic and 12.54 g potassium phosphate dibasic) and glycerol (4 mL per 1 L TB) and supplemented with 100 µg mL^−1^ ampicillin, was inoculated with an overnight starter culture to an initial optical density at 600 nm (OD600) of 0.1 and incubated at 37 °C with orbital agitation at 250 rpm until OD600 reached 0.7. Protein production was induced by a temperature shift to 30 °C. Cultures were then continued for an additional 16 h post-induction. Cells were subsequently harvested by centrifugation (7000 × *g*, 20 min, 4 °C), resuspended in lysis buffer (50 mm sodium phosphate buffer, pH 8.0, 300 mm NaCl, 20 mm imidazole) to an OD600 of 100 and disrupted with a constant cell disrupter (Constant Systems Ltd., Northands, UK). Cell debris was removed by centrifugation (40,000 × *g*, 90 min, 4 °C). Proteins were purified from the supernatant under native conditions by Ni-sepharose chromatography. Product-containing fractions were pooled and dialyzed against ultrapure water and then purified by anion exchange chromatography using a Q HP column. Protein-containing fractions were dialyzed extensively against ultrapure water. Purified proteins were frozen in liquid N_2_, lyophilized, and stored at −20 °C until further use.

### Characterization of SUPs

The concentrations of the purified polypeptides were determined by measuring absorbance at 280 nm using a spectrophotometer due to the presence of a Trp residue at the C-terminus of the SUP backbone (Spectra Max M2, Molecular Devices, Sunnyvale, USA). Product purity was determined by sodium dodecyl sulfate-polyacrylamide gel electrophoresis (SDS-PAGE) on a 10% polyacrylamide gel. Afterwards, gels were stained with Coomassie staining solution (40% methanol, 10% glacial acetic acid, 1 g L^−1^ Brilliant Blue R250). Photographs of the gels after staining were taken with an LAS-3000 Image Reader (Fuji Photo Film GmbH, Düsseldorf, Germany). The resulting stained gel is shown in Supplementary Fig. 2. The SUPs exhibit different electrophoretic mobility according to their charge and molar mass (Supplementary Table 1).

Mass spectrometric analysis was performed using a 4800 MALDI-TOF Analyzer in linear positive mode. The protein samples were mixed 1:1 v/v with sinapinic acid matrix (SIGMA) or alpha-cyano-4-hydroxycinnamic acid (100 mg mL^−1^ in 70% MeCN and 0.1% TFA). Mass spectra were analyzed with the Data Explorer software (version 4.9) and the data were plotted with Origin Pro 9.1. Values determined by mass spectrometry were in good agreement with the masses that were calculated (shown in Supplementary Fig. 3 and Supplementary Table 2) based on the amino acid sequence.

### Preparation of the SUP glue

An aqueous solution of the SUP with a concentration of ~220 μm (K18, K36, K72, K108, K144, GFP-K72, and mCherry-K72) was obtained by dissolving the lyophilized SUP in milliQwater. In a second solution made from ultrapure water, the concentration of SDBS lipid was adjusted to 10–20 mm at room temperature. Both solutions were combined in a 1:1 molar ratio so that ~1 mol of surfactant equals 1 mol of lysine residues within the SUP. Important to note, the critical micelle concentration of SDBS is 0.1 g L^−1^ (https://www.ams.usda.gov/sites/default/files/media/SDBSTR052617.pdf), which is far below the initial SDBS concentration in our prepared solution (~15 g L^−1^). Thus, the SDBS adopts micelles during complexation with the polypeptide component.

As a result of mixing, the transparent solution became cloudy because the SUP-SDBS complex segregated from the aqueous phase. After centrifugation, the SUP-SDBS complex sediments at the bottom of the vial as a coacervate and was separated from the aqueous supernatant. The supernatant was removed by a pipette and the SUP-SDBS glue material was collected.

When the original solution after complex formation was shaken heavily overnight, the solution became cloudy as the complex could not be re-dissolved in the aqueous medium. Due to this fact, smaller droplets formed that scatter light. This indicates that the occurrence of a coacervate in the system is a characteristic phase-separation phenomenon.

### Characterization of the SUP glues

#### Nuclear magnetic resonance spectroscopy

Proton nuclear magnetic resonance (^1^H-NMR) spectroscopy was employed to determine the optimal molar ratio of the two components in the SUP-SDBS system. The experiment was carried out by taking the short K18-SDBS complex as an example. The specific primary structure of the SUP, i.e., its repeating amino acid sequence (VPGKG)_n_, renders a quantitative evaluation of the ^1^H-NMR spectra via the integration of valine’s CH_3_ groups possible. In the K18-SDBS sample, both the solutions of SUP and SDBS were mixed in an aqueous solution with a molar ratio of lysine to surfactant of 1:1.

The analysis of the stoichiometry of the K18-SDBS complex by ^1^H-NMR (400 MHz) was performed in D_2_O/CD_3_OD (delay time: 10 s). The signal of aromatic ring protons (marked by a), methylene protons (marked by b) in SDBS and dimethyl group of Valine (marked by c) in the SUP were utilized to quantify the molar ratio of SUP and SDBS. If one SUP molecule can be combined with *n* SDBS molecules (SUP: *n*∙SDBS), then after complexation, the total number of protons (marked b + c) in K18-SDBS can be expressed as SUP (having 22 valine units, each valine carrying 2 CH_3_) × 6 + SDBS (having 3 CH_2_) × *n*. According to the integration of the protons of SDBS surfactant and SUP-SDBS in their ^1^H-NMR spectra as shown above, we have: $22\times 6+5.78n=13.66n$, where *n* can be determined to be 16.7. Therefore, SUP (K18): SDBS = 1:16.7 and the stoichiometric ratio of SDBS and lysine moiety is roughly 0.9:1, indicating that ~10% of lysine moieties are not complexed with a surfactant molecule.

To further confirm the NMR experiments, we have employed inductive coupled plasma mass spectrometry (ICP-MS) of sulfur atoms in the liquid supernatant during SUP glue complex formation. In this way, residual SDBS can be quantified. Three individual experiments were performed and the results showed that there is ~20% SDBS present in the supernatant compared to the SDBS solution before mixing with the peptide solution. The analysis by UV spectroscopy of the supernatant at 280 nm showed that there is ~10% SUP left compared to the starting solution, implying that not all components were incorporated into SUP-SDBS complexes during the coacervate formation. Since during the glue preparation the lysines within the SUP and SDBS are combined in equimolar ratio the above analysis suggests that 90% of the lysine residues in the coacervate (i.e., the glue) are involved in complexation with SDBS.

#### Thermogravimetric analysis

TGA was carried out using a TA Instruments Q1000 system in an N_2_ atmosphere and with a heating/cooling rate of 10 °C min^−1^. The TGA test is used to evaluate water content of the freshly prepared SUP-SDBS glue (here taking K72-SDBS as an example), collected from an Eppendorf vial (Supplementary Fig. 7).

#### Structure determination of the SUP glue

Polarized optical microscopy (POM) was conducted on a Zeiss Axiophot. Small-angle X-ray scattering (SAXS) was performed by employing a conventional X-ray source with radiation wavelength of *λ* = 1.54 Å and a Bruker Nano/microstar machine was used to obtain small-angle scattering profiles, where the sample-to-detector distance was 24 cm. The sample holder is a metal plate with a small hole (diameter ~0.25 cm, thickness ~0.15 cm), where the X-ray beam passes through. The SUP-SDBS complex was loaded into the hole by a pipette and was then sealed by kapton. The scattering vector *q* is defined as *q* = 4π sinθ λ^−1^ with 2θ being the scattering angle and the x-ray signal is further processed in FIT2D (version 18) software.

The absence of birefringence in the K72-SDBS sample indicated its disordered molecular packing (Supplementary Fig. 8). SAXS was used to investigate the average packing distance of the SUP-SDBS complexes. Based on a rough estimation of volumes and a comparison between TGA and SAXS experimental data, we estimate that the complex is composed of hydrated SUP units of ~2.2 nm thickness separated by SDBS surfactant domains of ~1.8 nm thickness.

### Mechanical characterization of the SUP glue

#### Evaluation of SUP glue with TGA

The freshly prepared SUP-SDBS complex was briefly freeze dried for 3–5 min prior to adhesion investigation. The water content of SUP glue before lap shear testing was characterized with TGA (Supplementary Fig. 9).

#### Evaluation of SUP glue with lap shear measurements

All lap shear measurements were carried out on different substrates including steel, glass, aluminum, PE, and PVC. Steel/aluminum substrates (10 × 0.5 × 0.2 cm) were sanded with 120 grit sandpaper, washed with soapy water, and rinsed with ethanol prior to testing. Glass, PE, and PVC substrates (10 × 0.5 × 0.2 cm) were cleaned with soap water, rinsed with deionized water, and dried overnight in air. After adding the glue onto one substrate, a second piece of substrate was then placed atop the first one to create a lap shear joint with an overlap area of 5 × 5 mm. The substrates were then allowed to cure 12 h at room temperature. Office clamps were used to hold the substrates together during the curing period.

Lap shear measurements were carried out with an INSTRON universal material testing system (model 5565) equipped with a 1000 N load cell, at a rate of 10 mm min^−1^ or 40 mm min^−1^. The bonding strength for each trial was obtained by dividing the maximum load (kN) observed at bond failure by the area of the adhesive overlap (m^2^), giving the bonding strength in Mega Pascals (MPa = 1000 kN m^−2^). Each sample was tested a minimum of three times and gave the average value.

#### Testing of commercial adhesive (cyanoacrylate)

Lap shear measurements for commercial cyanoacrylate glue as control experiments were conducted using the same method. For bulk dry lap shear measurements, all samples were cured for 12 h.

#### Investigation of adhesion performance of K72-SDBS complex with varied water ratios

To investigate the effects of the water content on adhesion performance of the SUP-SDBS glue, control experiments involving K72-SDBS complex with different water contents were carried out. In this regard, the K72-SDBS complexes were prepared with varying freeze-drying times (2–12 min) to yield a series of samples with different water contents. Lap shear measurements on steel substrates were conducted using the same method as described above. For bulk dry lap shear measurements, all samples were cured for 4 h. The weight ratio of water contents was calculated as following: Water content (%) = *W*_water_∙(*W*_water _+ *W*_complex_)^−1^.

#### Preparation of the K72-DNA glue

The lyophilized K72 was dissolved in milliQ water at a concentration of 2.14 mm (stock solution). Moreover, an aqueous solution of DNA was obtained by dissolving salmon sperm DNA in milliQ water at a concentration of 7.69 μm. For the production of the K72-DNA complex, the solution of K72 was combined with salmon DNA solution in a 1:6.7 volume ratio. In this way, 1 mol of phosphate of the DNA is combined with 1 mol of lysine residues of K72. After mixing, a cloudy solutions were formed. The complex was centrifuged at 14,500 × *g* for 5 min and separated from the supernatant. The K72-DNA complex was collected and freeze-dried for 10–15 min. Lap shear measurements for K72-DNA glue were then carried out on steel and glass substrates using the same method as for the SUP-SDBS glue.

#### Underwater lap shear measurements

To test underwater adhesion, steel substrates were polished before performing the lap shear measurements. Glass substrates were cleaned with soap water, rinsed with deionized water, and dried overnight in air. The SUP-SDBS complex was added atop of the two different substrates. After adding the complex onto one substrate, a second piece of the respective substrate was placed atop the first one to create a lap shear joint with an overlap area of 5 × 5 mm. In addition, clamps were used to hold the substrates together during the curing period. Then the substrates were immersed into water for 60 min. Finally, the bonding strength was measured as described above.

#### Proton nuclear magnetic resonance with SUP-SDBS complex in a molar ratio of 1:5

To investigate the underlying adhesion mechanism of the SUP-SDBS glue, ^1^H-NMR experiments were performed. For this, the SUP-SDBS complex was prepared from a starting ratio of lysine to surfactant of 1:5. The NMR measurements revealed a stoichiometry of 3.3 SDBS surfactant molecules per lysine within the resulting SUP-SDBS product.

The analysis of the stoichiometry of the K18-SDBS complex by ^1^H-NMR (400 MHz) was performed in D_2_O/CD_3_OD (delay time: 10 s). The signal of aromatic ring protons (marked by a), methylene protons (marked by b) in SDBS, and dimethyl group of valine (marked by c) in SUP were utilized to quantify the product molar ratio of SUP and SDBS. It was assumed that one SUP molecule can combine with *n* SDBS molecules (SUP : *n*∙SDBS). After complexation, the total number of protons (marked b + c) in K18-SDBS can be expressed as SUP (having 22 valine units à 2 CH_3_) × 6 + SDBS (having 3 CH_2_) × *n*. According to the integration of the protons of SDBS surfactant and SUP-SBDS in the ^1^H-NMR spectra, as shown above, we obtain: $22\times6+6.3n=8.5n$, where *n* equals 60. Thus, the stoichiometric ratio of SDBS and lysine moieties within K18 is roughly 3.3:1, indicating an excess of surfactant molecules being present within the complex.

#### Characterization of the adhesion of the SUP glue prepared with molar ratioof lysine to surfactant molecule of 1:5 and of 1:0.5

To investigate the underlying mechanism of the SUP-SDBS glue, control experiments involving K72-SDBS complexes with different molar ratios of lysine to surfactant were carried out. The K72-SDBS complexes were prepared with a starting ratio of lysine to surfactant of 1:5 and 1:0.5. For lap shear measurements with 12 h curing the same method as described above was used. For the lysine:SDBS ratio 1:5, experiments were performed on steel substrates while for the ratio 1:0.5 steel and PVC were employed.

A bilayer adhesion test was used to measure the adhesion energy. The adhesives tested with this method include SUP glues and cyanoacrylate. After adding the glue onto one substrate, a second piece of substrate was placed atop the first one to create a lap shear joint with an overlap area of 5 × 5 mm. The substrates were then allowed to cure for 12 h at room temperature. Office clamps were used to hold the substrates together during the curing period. The measurements were carried out with an INSTRON universal tensile tests system (model 5565) equipped with a 1000 N load cell, at a rate of 10 mm min^−1^. At the same time stress-strain curves were recorded. In our system, the adhesion energy *E* can be calculated by using a simplified equation according to previous reports^44^: *E* = *F*(*λ*−1), where *F* and *λ* are the breaking force per unit width (the force in the current state divided by the width of the sample in the pristine state) and the breaking extensibility after stretching, respectively.

### Molecular force measurements using surface forces apparatus (SFA)

The normal force-distance profiles and adhesion forces between two components (namely cationic polypeptide and anionic surfactant) that form the bio-glue were determined using an SFA. The typical experimental setup and working principle of SFA have been reported previously^45–48^. Briefly, thin back-silvered mica sheets (thickness 1–5 μm) were first glued onto cylindrical silica disks (radius *R* = 2 cm). Both polypeptide and surfactant coatings were prepared by drop coating method. For polypeptide coatings, 100 μL polypeptide aqueous solution (50 μg mL^−1^) was placed on mica substrates. After 20 min adsorption, the mica surfaces were thoroughly rinsed by Milli-Q water in order to remove unbound or loosely bound polypeptides. For surfactant coatings, mica surfaces were first pretreated with (3-aminopropyl) triethoxysilane (APTES) ethanol solution (5% w/w) for 20 min to form APTES-grafted mica substrates. The modified mica substrates were drop-coated by 100 μL surfactant aqueous solution (50 μg mL^−1^) and incubated for 20 min, followed by a thorough rinsing with Milli-Q water. One polypeptide-coated mica surface and one surfactant-coated mica surface were mounted into the SFA chamber in a crossed-cylinder geometry, the interaction of which is equivalent to a sphere of radius *R* approaching a flat surface when their separation *D* is much smaller than *R* based on the ‘Derjaguin approximation’^49^. Desired testing aqueous solution was then injected between two surfaces and the system was allowed to equilibrate for 30 min before force measurements. The normal interaction forces *F* between the two curved surfaces were measured as a function of surface separation distance *D*. Adhesion was measured when the two attractive surfaces were separated and jumped apart from each other (so-called “jump out”). *D* could be monitored in real time and in situ using an optical technique called multiple-beam interferometry (MBI) by employing the fringes of equal chromatic order (FECO)^50^. The reference distance (*D* = 0) was defined as the contact of two bare mica surfaces in air. The coating thickness *D*_T_ could be determined via the shift of FECO wavelength before and after coating. *F* was determined according to the Hooke’s law^50^. During a typical force measurement, two surfaces were first brought to approach each other (“approach”) and were kept in contact for a certain time followed by separation (“separation”). The adhesion *F*_ad_ measured during separation is related to the adhesion energy per unit area *W*_ad_ for two flat surfaces of the same materials based on the Johnson−Kendall−Roberts (JKR) model *F*_ad_/*R* =1.5∙π∙*W*_ad_, which is generally applied for soft deformable surfaces with relatively large curvature and strong adhesion ^51–53^.

### Computer simulations of SUP-SDBS and SUP-SDS complexes

All-atomistic simulations were conducted in NVT ensemble using GROMACS 2018 package^54^. We used force field Optimized Parameters for Liquid Simulation - All Atomfor simulations of peptides, surfactants, ions, and TIP3P for water^55–57^. The integration of equations of motion was performed by using Verlet algorithm with time step 1 fs^58^. The shot-range electrostatic and Lennard-Jones interactions were calculated with a cutoff radius of 1.2 nm. The particle mesh Ewald technique was used for the long-range electrostatic interactions. All bonds involving hydrogen are constrained using a LINCS algorithm^59,60^. The velocity rescale temperature coupling scheme was employed for NVT ensemble at 310 K and time constant 0.01 ps. The cubic box size was varied from 8 to 25 nm depending on the number of SUPs in the simulations. The periodic boundary conditions were imposed in all dimensions. We used the pentapeptide (VPGKG)_n_ as a repeating unit, where V is a valine, P proline, G glycine, and K lysine, respectively. The primary amine of the lysine was protonated in all repeating units. The overall electric neutrality of the system was provided by OH^−^ counterions. The terminal group for the peptides was hydrogen. Computer simulations were performed for K18, K36, and K72 molecules. Fully elongated SUP molecules in explicit water and OH^-^ counterions were equilibrated during 50 ns of simulation. The equilibration was accompanied by shrinkage of the SUPs. Then sodium dodecylbenzene sulfonate (SDBS) or sodium dodecyl sulfonate (SDS) molecules with different molar ratio to lysine 1:1, 0.89:1, 0.78:1, and 0.67:1 were added. They were homogeneously distributed throughout the simulation box. Further annealing (simulation) proceeded during 80 ns, which was accompanied by electrostatics-driven complexation of surfactant with the SUPs. Snapshots of the complexes are shown in Supplementary Fig. 19. Each peptide in the complex looks rather single than aggregated with each other. The integrity of the complex is provided by surfactant nanodomains, which bind different peptides. The x,y,z-components of the average gyration radius of the single peptide in the complex as a function of the number of repeating units *n* = 18–72 are shown in Supplementary Fig. 20A–C. The difference in the x,y,z-components means anisotropy of the peptides in the complexes, especially for high values of n. For *n* = 72, the peptides are characterized by a radius of ~1 nm (thickness ~2 nm), which correlates with the above SAXS data (~2.2 nm).

To study the cohesion strength of the complexes, we used five molecules of K18 combined with the different surfactants SDBS and SDS. Different molar ratios of lysine to surfactant in the complexes were investigated. Classical MD simulations were used for SUP-surfactant complexes in an aqueous solution. Moreover, we applied the TIP3P water model. The protein and surfactant force field parameters were taken from OPLS-AA. The box size was 20 × 10 × 10 nm^3^. The complexes were first annealed during 100 ns of simulation. The whole system contains five K18-proteins and different number of surfactant molecules (90, 80, 70, 60, and 50). Forces were applied to the center of mass of second and fourth protein in the aggregate and had a constant value (Supplementary Fig. 19A). If the value of the applied forces was not high enough for separation, the simulation was repeated with higher values until the splitting of the complex occurred. The amounts of lysines in the protein backbone were simulated to verify the peak performance of the SUP glue systems (Supplementary Fig. 19B). In this context, Lys12 (*a* = 4, *b* = 6), K18 (*a* = 1, *b* = 9) and Lys20 (*a* = 0, *b* = 10) were employed, where a and b indicate relevant parameters in the molecule [(GVG**V**P)_a_(GVG**K**P)_b_]_2_. Compared to K18, Lys12 exhibits a higher valine content, while Lys20 completely consists of GVGKP units. The results show that K18-SDBS complex reaches a higher critical force than the other samples. This result implies that a delicate ratio of hydrophobic interaction and electrostatic force is important for the optimal performance of the system.

Snapshots of equilibrium stoichiometric SUP-SDBS complexes show the formation of disordered nanodomains of isolated SUPs bound to each other by surfactant micelles (Supplementary Fig. 19C). Splitting of the material under applied external forces (black arrows Supplementary Fig. 19C) proceeded mainly via disaggregation of hydrophobic domains of the SDBS due to weaker van der Waals interactions compared to electrostatic bonds (Movie S1). It was demonstrated that a maximum force is needed at ~0.9 molar ratio of SDBS to lysine (Supplementary Fig. 19D), which is in very good agreement with the experimental findings. Independent from the fraction of surfactants, SUP-SDS complexes disintegrated at much smaller applied forces compared to complexes involving the aromatic surfactant (Supplementary Fig. 19D). Thus, the presence of phenyl rings improves the cohesive strength of the complex via attractive π-π- and cation-π interactions.

To study π-π interaction in the micelles, we calculated the number of contacts between aromatic groups. By contact, we mean the presence of groups at a characteristic distance for each interaction. There are three types of π–π interactions—sandwich type (S-type, face to face, *R*_S _= 0.4 nm), T-type (edge to face, *R*_T _= 0.5 nm) and parallel displaced type (PD-type, R_PD _= 0.39 nm)^61^. We observed no S- or PD- types in our simulation but only T-type, which is the weakest type of π-π interaction and corresponds to −5 kcal mol^−1^ binding energy. The number of the stacked benzene rings was estimated for a different surfactant ratio with the characteristic distance of 0.5 nm (Supplementary Fig. 20D). The calculation of the radial distribution function between benzene rings is in agreement with Sinnokrot. There were 18 ± 6 % T-type aromatic pairs in case of 1:1 complexation ratio of lysineto surfactant (Supplementary Fig. 20E). Though there are also hydrophobic interactions between the alkyl tails of SDBS, the observed T-type interactions could provide more toughness. The assembly of SDBS molecules is dictated by hydrophobic interactions between their alkyl tails. A similar behavior is seen for SDS. However, the SDBS glue system, provided more toughness within the SDBS glue system originating from a few T-type interactions.

In our case there are two types of cation-anion pairs and all charged groups form small complexes with 5–6 NH_3_^+^, SO_3_^−^ and a few Na^+^ and OH^-^ generally. The typical area of charged groups is shown in Supplementary Fig. 20F. The cationic part of lysine can interact with the anion of one SDBS molecule and the benzene ring of another SDBS molecule in the area. Obviously, the main driving force in SUP-SDBS/SDS complexation is electrostatic interaction. We only examinedπ - π and cation- π pair interactions. Aromatic interactions (18 ± 6% of benzene rings, Supplementary Fig. 20E) are presented in the form of edge to face, i.e. T-type. The calculation showed that 17.4 ± 3% of NH_3_^+^ groups are at a characteristic distance of 0.4 nm (from the radial distribution function, Supplementary Fig. 20F) from the benzene rings in the glue with a 1:1 molar ratio lysine to surfactant (Supplementary Fig. 20G). This distance corresponds to the binding energy −5 kcal mol^−1^ ^62,63^.

### Cytotoxicity evaluation of the SUP Glue

Both cytotoxicity of SUPs and SUP-SDBS complexes were evaluated. XTT cell viability assay was used for biocompatibility test of K72-SDBS (Supplementary Fig. 25). Briefly, 5 × 10^3^ HeLa cells per well were seeded in a 96 well plate and grown overnight. Various concentrations of K72-SDBS complex immersed in 100 µL culture medium (DMEM with 10% FBS) were incubated with the cells for 72 h at 37 °C, 5% CO_2_ (in triplicate). 50 µL of XTT solution mixed with PMS was added to each well, afterwards the plate was incubated for 2 h. Absorbance at 450 and 630 nm was recorded. Statistical analysis was performed using *t*-test with GraphPad Prism 7.0 program (*n* = 3).

CellTiter-Glo cell viability assay was used for cytotoxicity test of SUPs. HeLa cells were seeded as above. K144, K108, K72, K36, and K18 at various concentrations dissolved in 100 µL culture medium (DMEM with 10% FBS) were incubated with cells for 72 h at 37 °C, 5% CO_2_ (in triplicate). 100 µL CellTiter-Glo Reagent was added to each well with 2 min mixing and 10 min signal stabilization time at room temperature. Then luminescence was recorded (Supplementary Fig. 26). Statistical analysis was performed using *t* test with GraphPad Prism 7.0 program.

To assess cytotoxicity that reflects the situation during the gluing process, 3D cell culture tests involving SUP-SDBS glue and fluorescence-based live/dead staining were performed (Supplementary Fig. 27). Human fibroblasts (HFB), Hela cells, and bone marrow-derived stem cells (BMSC) (Pcocell Life Science & Technology) were cultured in DMEM medium supplemented with 10%FBS. Cells were seeded at a density of 1.5 × 10^5^ per well onto 25mm circle microscope cover slides positioned at the bottom of 24-well plates. Afterwards, SUP-SDBS glue was applied on top of the cells by a pipette tip. PBS and SUP-DNA glue were treated similarly representing controls for SUP-SDBS glue samples. After incubation with glue for 24 h, medium was removed and each well was washed three times with PBS buffer. Then, 1 µL of calcein-AM (AM, 1 mg mL^−1^ solution in H_2_O) that stains cytoplasm and membrane of living cells by a specific enzymatic reaction indicating active living cells and 1 µL of propidium iodide (PI, 1 mg mL^−1^ solution in DMSO) that stains apoptotic cells by inserting into nuclear DNA were added and incubated for 20 min. After incubation with PI and AM, dyes were removed and each slide was taken out and washed three times with PBS buffer. Afterwards, the slides of cells with SUPs-SDBS glue and controls were photographed at emission wavelengths of 495 nm for AM and 539 nm for PI by LSCM. It is important to note that the PI dye can also interact with other non-cellular DNA. Thus, the background in DNA-glue groups were shown in red to some extent due to the dye staining the salmon sperm DNA component of the glue.

For the cell encapsulation in the alginate gel, sodium alginate dissolved in 100 µL culture medium (DMEM with 10% FBS) at a concentration of 1% wt was incubated with 1.5 × 10^5^ HeLa cells per well onto 25 mm circle microscope cover slides positioned at the bottom of 24-well plates. Then, 10 µL calcium chloride (1% wt) was added into the well and incubated for 24 h at 37 °C, 5% CO_2_ (in triplicate).

### Ex vivo adhesion model tests on porcine skin and human eyelids

Fresh and clean porcine skin was cut into pieces with dimensions of 10 × 0.5 × 0.2 cm. SUP glue (GFP tagged for easy tracking) was applied on the surface of the skin. After adding the glue onto one piece of the sample, a second one was placed atop the first one to form a lap shear joint with an overlap area of 5 × 5 mm. The substrates were then allowed to cure for 10 min at room temperature. Office clamps were used to hold the skin substrates together during the curing period. The lap-shear mechanical characterization was performed similarly as in the protocol detailed above.

#### Adhesion test on wet tissue

Besides employing the glue on hard substrates, the SUP-SDBS complex was applied on soft biological tissue. Therefore, the surface of tissue samples (skin, liver, heart, and muscle) was cleaned with filter paper. After application of the glue on one tissue substrate, the second substrate was placed atop and pressed for 10 s with 1 N weight. Afterwards, the samples were cured for 1 h. To measure shear strength, adhered tissues with a joint area of 5 × 5 mm were characterized by a SHIMADZU testing machine (500 N loading cell), at various speeds of 10, 50, 100, and 600 mm min^−1^. The results are shown in Fig. 3A and Supplementary Fig. 28.

#### Cosmetic adhesion application

As shown in Fig. 3D, a plastic paraffin film coated with SUP glue was firmly applied to the skin on the arm. The SUP glue combines strong adhesion and deformability, rendering it particularly suitable for transdermal drug delivery, wearable device assembly, or wound dressings. Furthermore, the SUP glue was applied to the skin of the eyelid to achieve a long-lasting and reversible creased upper eyelid effect (Fig. 3D and Supplementary Fig. 29). This cosmetic transformation is very popular in Asian cultures where even surgical interventions are undertaken to transform single-eyelid to double-eyelid appearance^64,65^. In this context, SUP glue might be useful to reduce ptosis and sagging skin, to recover peripheral vision, and to circumvent blepharoplasty^66,67^. Importantly, the adhesion effect of SUP glue on skin endures during two days, meanwhile being readily cleaned with excess water owing to the non-covalent and reversible chemical adhesion (Supplementary Fig. 29).

The study protocol for producing double eyelids was approved by the Ethic Committee of the Second Hospital of Jilin University. A male volunteer with a single eyelid was used for this study. 5–10 mg of SUP glue were applied on one of the upper eyelids. A crescent-shaped double eyelid of 1.5 cm length was formed in <60 s curing. Stretching the adhered region clearly showed that the skin of the eyelid firmly stuck together (Fig. 3D), indicating robust and efficient adhesion of SUP glue on human skin. The artificial double eyelid was maintained in its shape for up to 48 h. Besides, a pilot test on the cleaning of SUP glue in the eyelid region was performed. The glue can be removed readily with an excessive amount of water (5 mL applied on cellulose tissue), which is consistent with in vitro data in Supplementary Figs. 23 and 24.

### In vivo linear wound hemostasis and healing

The lethal dose of SDBS for rats is 1.26 g kg^−1^. There was no significant toxic effect observed when rats were given oral doses of 1000 ppm in water, i.e., 1 g L^−1^, and no systemic toxicity was observed over 28 d. Typically, a rat weighs ~300 g and the threshold dose is thus ~400 mg. For the applied glue in the wound area of the rat, ~3 mg of SDBS was involved. Therefore, the toxicity and safety of SDBS is of little concern in regard to the SUP glue system.

In vivo skin wound sealing and healing: linear wounds were produced by a scalpel with a dimension of l × h × w = 2 × 1 × 0.5 cm^3^ to evaluate the effect of SUP glue on wound sealing and healing of rat skin. Animal experiments were approved by Jilin University Institutional Animal Care and Use unit. Healthy female Wistar rats (180–200 g) were purchased from Beijing HFK Biotechnology Ltd. First, the rats were randomly divided into five groups (*n* = 3) and anesthetized with chloral hydrate (10 wt.%). After 10 min, the rats were completely anesthetized. The back areas of rats were depilated with VEET hair removal cream and disinfected with 75% disinfecting ethanol. The wounds formed by a scalpel, except the untreated control group, were treated with SUP glue, saline, suture, and medical adhesive COMPONT®, respectively. Thereafter, each group of the rats was individually housed. The photographs of the wounds were taken with a digital camera every day. Then the rats were euthanized, and fresh portions of the wound site from each rat were harvested rapidly on the ninth day. Then they were fixed in neutral buffered formalin (10%). After that, the samples were dehydrated using grades of ethanol (70, 80, 90, 95, and 100%). Lastly, the samples were impregnated with molten paraffin wax, embedded, and blocked out. The tissue sections were cut into 2–4 microns thickness and mounted on the glass slides. The H&E staining, Masson’s trichrome staining, and immunofluorescent staining with IL-6 and TNF-α were performed with protocols as described in literature^21,68^. The photographs of stained sections were taken by using an optical microscope (Nikon, Japan).

In vivo liver wound hemostasis: healthy rats with a weight of 180 g (three rats for one trial) were randomly chosen and were anesthetized with chloral hydrate (10 wt.%). After 10 min, the rats were completely anesthetized. The abdomen and chest of the rats were disinfected with 75% disinfecting ethanol. Then, the chest of rats was opened, and the surface of the heart and the liver were exposed. The liver of the rats was punctured to bleed with a needle (1.2 mm diameter). SUP adhesive was rapidly applied to the sites of the punctures. The video of the process of ceasing bleeding was recorded with a camera (Movie S2).

The histological scoring of the data was performed in blind setup (Supplementary Table 5). The linear wound healing was evaluated independently by three pathologists from medical college or clinical units. Regarding the scoring rules, four parameters were evaluated, including degree of skin repair, inflammatory cell infiltration, newborn capillaries, and collagen levels near the wound area. The results indicate that the group using SUP glue shows improved wound healing compared to the blank and sutured groups. The blind scoring for round-shape wound healing was carried out in the same way (Supplementary Table 6).

### Wound hemostasis in tiny pig model

Wound hemostasis in tiny pig model: Healthy female tiny pigs (30–40 kg) were purchased from Changchun Muzilongsheng Biotechnology Ltd. and randomly chosen (three pigs for one trial). The pigs were anesthetized with pentobarbital sodium (3% wt. 1 mL kg^−1^) and Alphadione® (0.14 mL kg^−1^). After 20 min, the tiny pigs were completely anesthetized. The skin of the abdomen and chest sterilized with 75% disinfecting ethanol was removed and an incision was made in the mid-abdomen near the ventral side. Then, the liver and kidney were exposed and punctured with a needle (24 mm diameter). For the hemostasis of the heart, a large incision was made in the chest to open the whole thorax and punctures were made located in the tip of the heart. The punctures were rapidly smeared with SUP glue and Histoacryl®, respectively. The process of hemostasis in the liver, kidney, and heart was recorded by a camera. To evaluate the hemostatic ability of SUP glue and Histoacryl®, after the glue was smeared completely over the defect, hemostatic time was recorded until the blood clotting was observed.

### In vivo round-shape wound dressing test

We induced wounds of round shape and a diameter of 10 mm on the back of rats with a specific puncher. After 6 d, excellent wound repair and skin regeneration were detected for the SUP-glue group compared with saline, cyanoacrylate, and no treatment groups in Fig. S33A. These results were supported by histopathologic analysis which were performed on the 7th day (Supplementary Fig. 33B). There were many new blood vessels formed and abundant granulation was detected in tissue treated with the SUPglue. Moreover, we performed Masson’s trichrome staining. There was more mature and compact collagen content in the group of SUP glue compared to other control groups shown in Supplementary Fig. 35. IL-6 and TNF-α were used to characterize the level of inflammation. The data not only indicate the anti-inflammatory effect of SUP glue, but also prove its good biodegradability because almost no GFP signal was detected in the experiments performed with GFP-K72-SDBS (Supplementary Figs. 36 and 37).

### Reporting summary

Further information on research design is available in the Nature Research Reporting Summary linked to this article.

## Supplementary information

Supplementary Information

Peer Review File

Description of Additional Supplementary Files

Supplementary Movie 1

Supplementary Movie 2

Supplementary Movie 3

Supplementary Movie 4

Supplementary Movie 5

Supplementary Movie 6

Supplementary Movie 7

Supplementary Movie 8

Reporting Summary

## Data Availability

The authors confirm that the data supporting the findings of this study are available within the article (Figs. 1–4) and/or its supplementary materials (Supplementary Figs. 1–38, Tables 1–6 and Movie 1–8). Data are also available from the corresponding authors upon reasonable request.
