# Supplementary material for: Ultra-strong bio-glue from genetically engineered polypeptides
Source: Nat Commun. 2021 Jun 14;12:3613. doi: 10.1038/s41467-021-23117-9 (PMC8203747; doi:10.1038/s41467-021-23117-9)
Supplement: Supplementary file 1 — Supplementary Information [file 41467_2021_23117_MOESM1_ESM.pdf]

Supplementary Information

Ultra-strong bio-glue from genetically engineered polypeptides

Chao Ma,[1,2,3†] Jing Sun,[1†] Bo Li,[4†] Yang Feng,[4] Yao Sun,[4] Li Xiang,[5] Baiheng Wu,[6] Lingling Xiao,[4] Baimei Liu,[4] Vladislav S. Petrovskii,[7,8] Bin Liu,[4] Jinrui Zhang,[1] Zili Wang,[1] Hongyan Li,[2,9] Lei Zhang,[4] Jingjing Li,[4] Fan Wang,[4] Robert Göstl,[9] Igor I. Potemkin,[7,8,9] Dong Chen,[6] Hongbo Zeng,[5] Hongjie Zhang,[1,4] Kai Liu[1,4]* and Andreas Herrmann[2,9,11]*

[1]Department of Chemistry, Tsinghua University, 100084, Beijing, China.

[2]Zernike Institute for Advanced Materials, University of Groningen, Nijenborgh 4, 9747 AG Groningen, The Netherlands.

[3]School of Engineering and Applied Sciences, Harvard University, 29 Oxford Street Cambridge, MA 02138, USA.

[4]State Key Laboratory of Rare Earth Resource Utilization, Changchun Institute of Applied Chemistry, Chinese Academy of Sciences, 130022, Changchun, China.

[5]Department of Chemical and Materials Engineering, University of Alberta, Edmonton, Alberta T6G 1H9, Canada.

[6]Institute of Process Equipment, College of energy engineering, Zhejiang University, Hangzhou 310027, China.

[7]Physics Department, Lomonosov Moscow State University, Moscow 119991, Russian Federation.

[8]N. N. Semenov Institute of Chemical Physics, Russian Academy of Sciences, Moscow 119991, Russian Federation.

[9]DWI - Leibniz Institute for Interactive Materials, Forckenbeckstr. 50, 52056 Aachen, Germany.

[10]National Research South Ural State University, Chelyabinsk 454080, Russian Federation.

[11]Institute of Technical and Macromolecular Chemistry, RWTH Aachen University, Worringerweg 1, 52074, Aachen, Germany.

*kailiu@tsinghua.edu.cn; herrmann@dwi.rwth-aachen.de

†These authors contributed equally.

Table of contents

1. Molecular cloning and SUP expression 3
2. Characterization of SUPs 3
3. Preparation of the SUP glue 6
4. Characterization of the SUP glues 8
5. Mechanical characterization of the SUP glue 10
6. Molecular Force Measurements using surface forces apparatus (SFA) 22
7. Computer simulations of SUP-SDBS and SUP-SDS complexes 23
8. Biodegradability and recyclability of the SUP glue 25
9. Cytotoxicity Evaluation of the SUP Glue 27
10. *Ex vivo* adhesion model tests on porcine skin and human eyelids 29
11. *In vivo* linear wound hemostasis and healing 30
12. Wound hemostasis in tiny pig model 33
13. *In vivo* round-shape wound dressing test 35

1. Molecular cloning and SUP expression

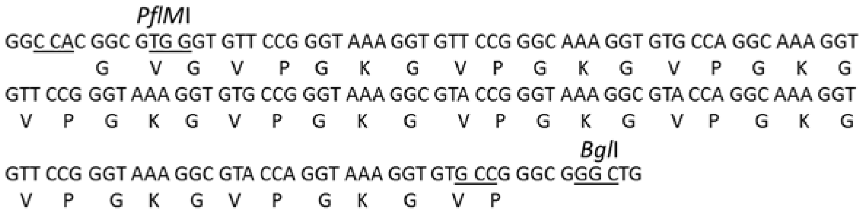

Supplementary Figure 1. Genes and corresponding polypeptide sequences of SUP K9 (containing nine lysine residues). Restriction sites flanking the insert gene are *PflMI*and *Bgl*I.

2. Characterization of SUPs

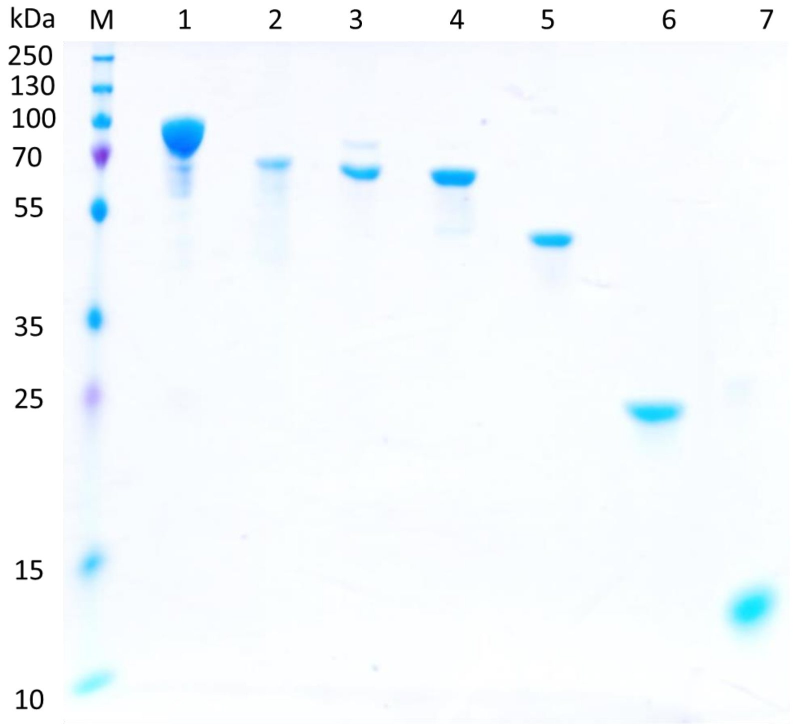

Supplementary Figure 2. SUP samples used in this study characterized by SDS-PAGE. M, PageRuler plus prestained protein ladder. Lane 1-7: K144, K108, mCherry-K72, GFP-K72, K72, K36 and K18. The electrophoretic behavior of the SUP polypeptides with a high net charge is different from folded proteins, which usually exhibit balanced charges as present in the marker lane M. Three times each experiment were repeated independently with similar results.

Supplementary Table 1. General information of supercharged proteins used in this work.

Name of SUPs	Isoelectric point (PI)	Sequence	Molar mass (Da)
K18	9.35	GAGP[(GVGVP)(GKGVP)$_9$]$_2$GWPH$_6$	10176
K36	11.54	GAGP[(GVGVP)(GKGVP)$_9$]$_4$GWPH$_6$	19019
K72	11.85	GAGP[(GVGVP)(GKGVP)$_9$]$_8$GWPH$_6$	36313
K108	12.03	GAGP[(GVGVP)(GKGVP)$_9$]$_{12}$GWPH$_6$	53870
K144	12.16	GAGP[(GVGVP)(GKGVP)$_9$]$_{16}$GWPH$_6$	71294
GFP-K72	10.20	GFP- GAGP[(GVGVP)(GKGVP)$_9$]$_8$GWPH$_6$	63910
mCherry-K72	10.18	mCherry-GAGP[(GVGVP)(GKGVP)$_9$]$_8$GWPH$_6$	63286

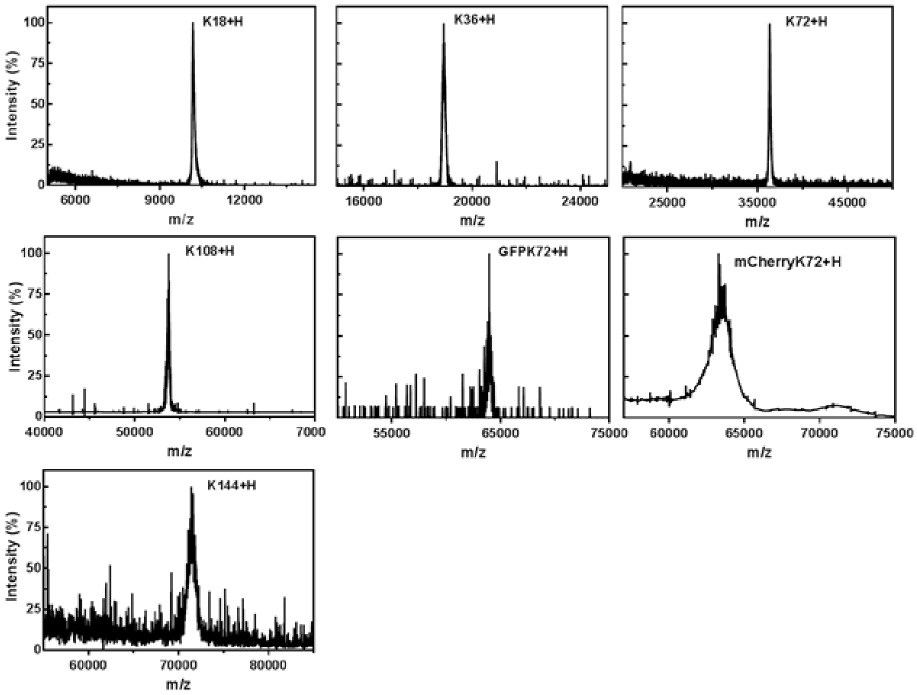

Supplementary Figure 3. MALDI-TOF mass spectra of the SUP samples.

Supplementary Table 2. Mass determination of supercharged polypeptides. *average molar mass calculated with ProtParam (https://web.expasy.org/protparam/). #molar mass determined by MALDI-TOF mass spectrometry.

	$M_{calculated}$* (Da)	M_{MS}# (Da)
K18	10176	10162 +/- 50
K36	19019	18975 +/- 50
K72	36313	36348 +/- 50
K108	53870	53858 +/- 50
GFP-K72	63910	63963 +/- 100
mCherry-K72	63286	63281 +/- 100
K144	71294	71321 +/- 100

3. Preparation of the SUP glue

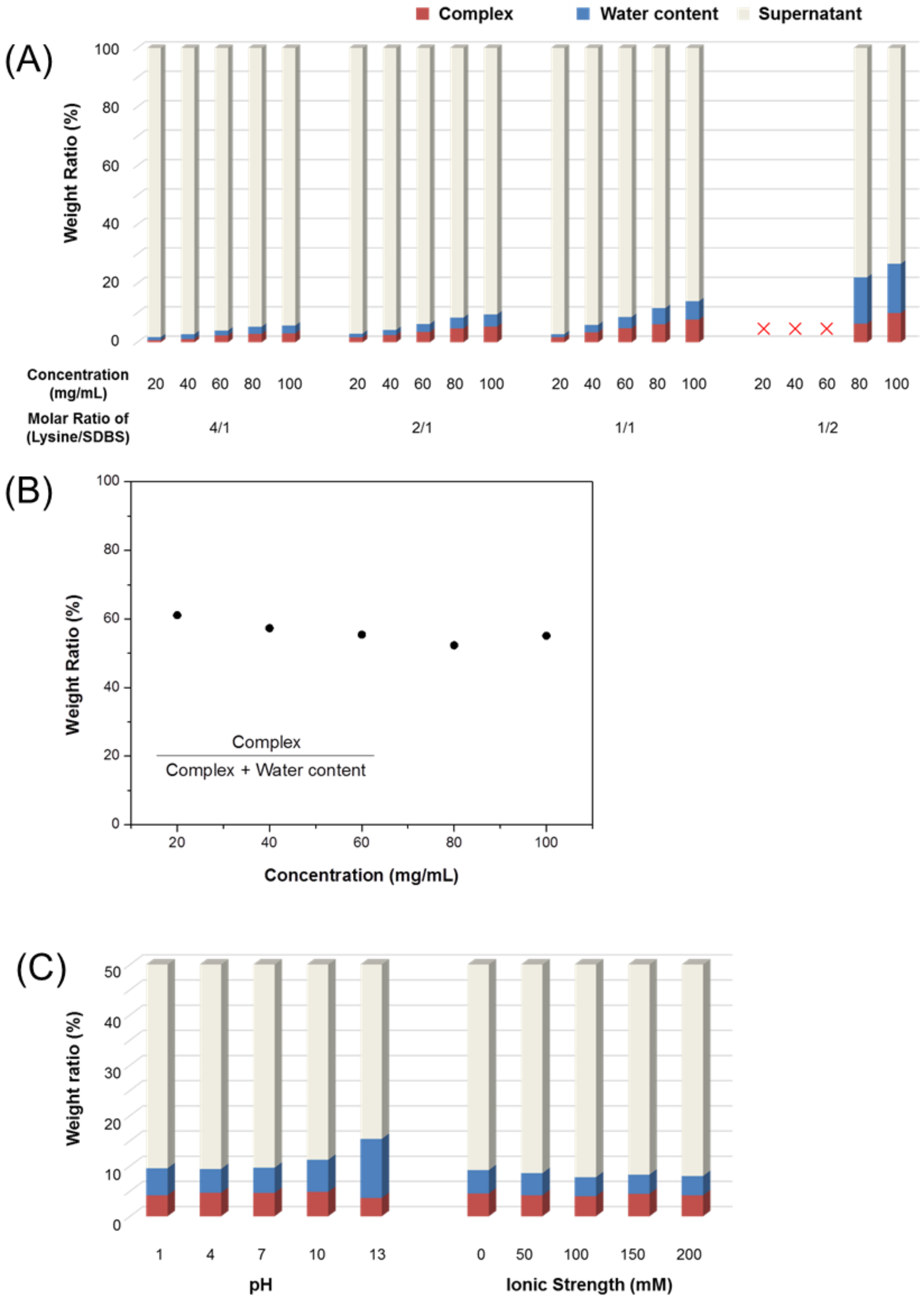

Supplementary Figure 4. Phase diagrams of SUP glue. Here, K72–SDBS complexes are shown as an example. (A) Dependence of the coacervate formation on the lysine:SDBS molar ratio, which is the molar ratio of lysine in K72 protein to sulfonate group in SDBS. The concentration on the x-axis represents the total concentration of protein and SDBS. The weight ratio on the y-axis shows the weight ratio of complex, water content and supernatant of the whole system. The coacervates are the sum of K72-SDBS complex and water content. (B) The weight ratio of SUP-SDBS complex in the coacervates, *i.e.* complex/(complex+water content), as a function of the total concentration of protein and SDBS. The lysine:SDBS molar ratio was 1:1. (C) Dependence of the coacervate formation on pH and ionic strength. The lysine:SDBS molar ratio was 1:1. The total concentration of protein and SDBS was 100 mg·mL^{-1}. The red, blue and gray columns represent the weight ratios (wt%) of SUP-SDBS complex, water content and supernatant in the whole system, respectively.

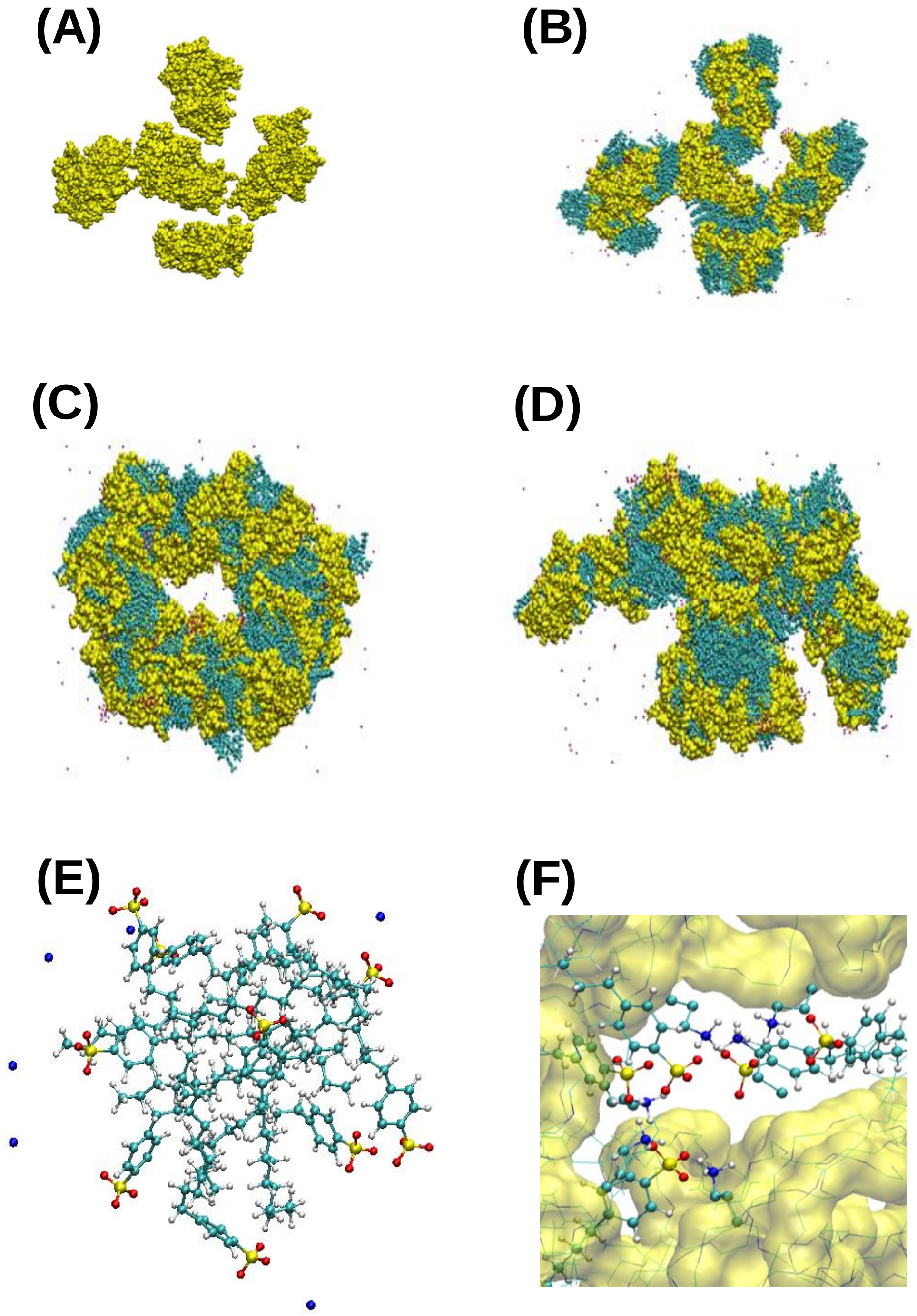

Supplementary Figure 5. Computer simulations and snapshots of equilibrium stoichiometric SUP-surfactant complexes. (A) and (B) the complexes formed by 5 molecules of K36. SDBS molecules and ions are not shown in (A), and shown in (B). (C) and (D) show SUP-SDBS complexes formed by 20 molecules of K18 and are depicted from two different angles. The SUP and SDBS domains are shown in yellow and cyan, respectively. Counterions and cations are indicated by dots. (E) SDBS micelle formed in the solution are shown by simulation. (F) A local area inside the complex showing the interaction profile of SDBS and protein backbones. Na^+, OH^- and water are not shown. The SUP molecules and surfactants are shown in yellow and cyan, respectively. Sodium ions are drawn as blue dots.

4. Characterization of the SUP glues

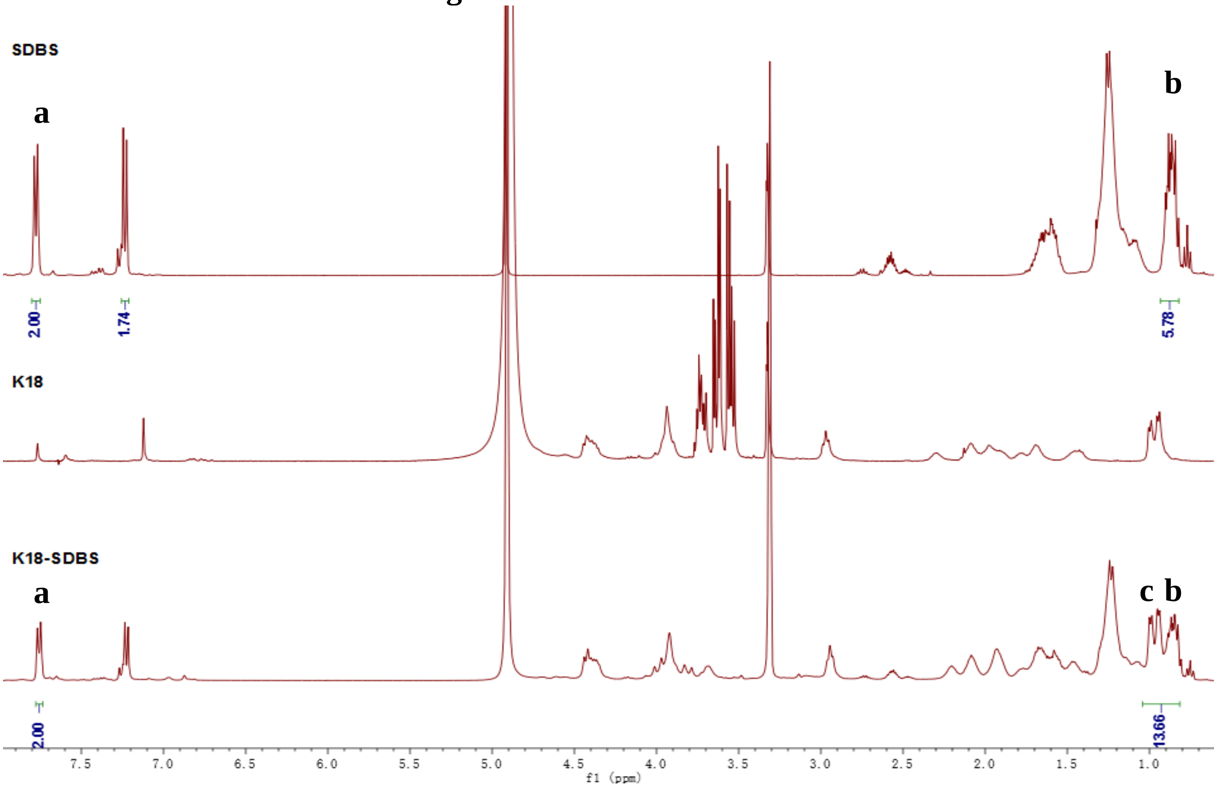

Supplementary Figure 6. Proton Nuclear magnetic resonance (^{1}H-NMR) spectroscopy characterization of SUP glue systems (Delay time: 10 s). Here K18-SDBS adhesives with a molar ratio of 1:1 was used a representative example.

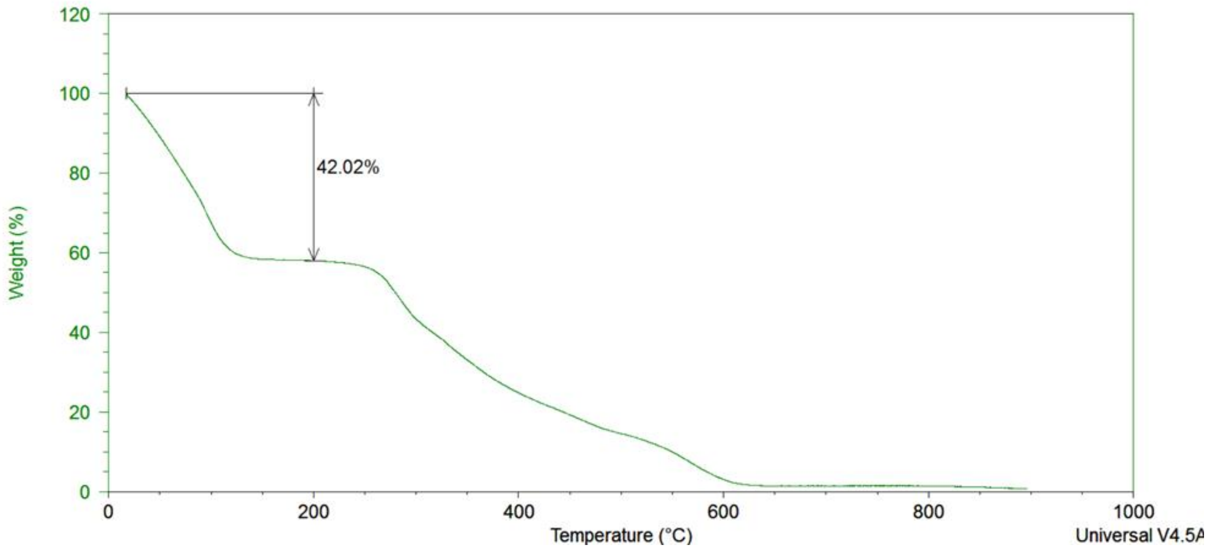

Supplementary Figure 7. TGA characterization of the K72-SDBS complex. After removing the supernatant, the complex was transferred to a specific chamber for TGA analysis. It is evident that ca. 42% water content is remaining in the complex.

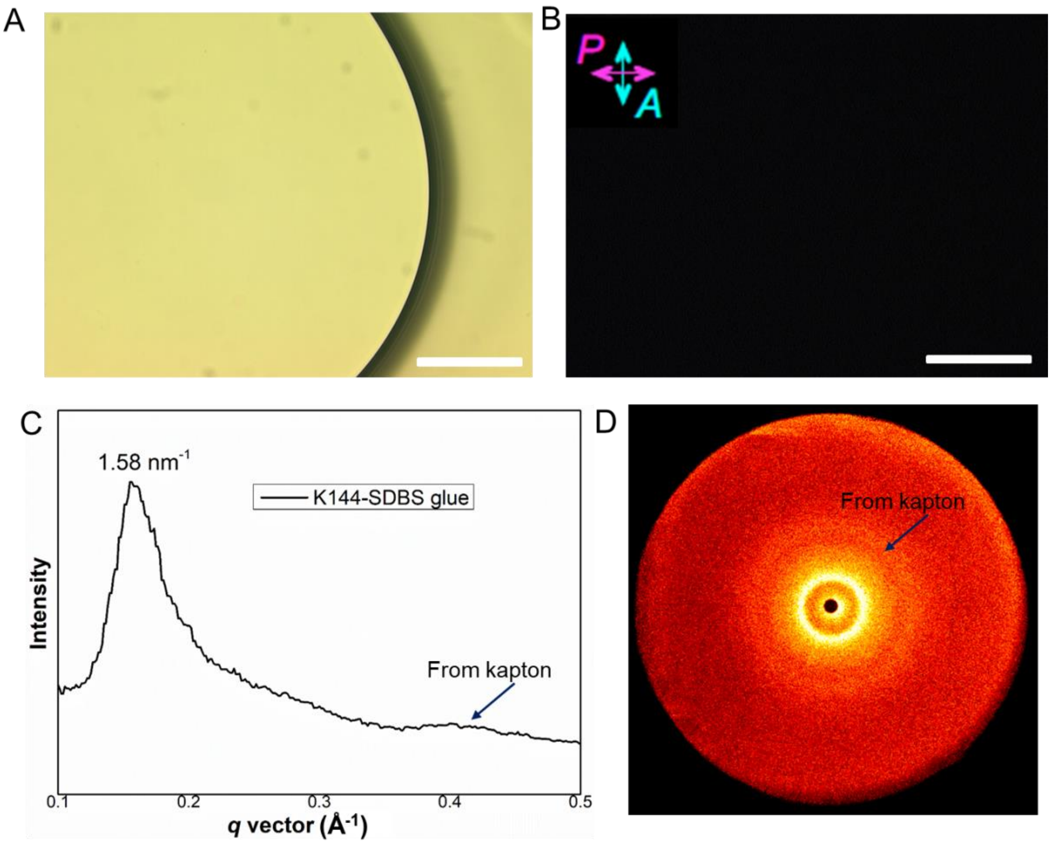

Supplementary Figure 8. Characterization of the SUP-SDBS coacervate (here taking K144-SDBS as an example). (A) Optical image of the liquid and (B) the corresponding POM. No birefringence was observed in case of the K144-SDBS sample, indicating its disordered molecular packing. Three times each experiment were repeated independently with similar results in (A) and (B). (C, D) SAXS analysis of the K144-SDBS liquid. The broad diffraction peak at $q \approx 4$ nm^{-1} is due to the Kapton, which was used for sealing of the SUP-SDBS fluid sample. SAXS profile showed one broad diffraction peak corresponding to a d spacing of 40.0 Å. Based on a rough estimation of volumes and comparison between TGA and SAXS experimental data, the complex is composed of hydrated SUP units of ~2.2 nm thickness separated by regions containing disordered SDBS surfactant molecules of ~1.8 nm thickness. Scale bar: 100 µm.

5. Mechanical characterization of the SUP glue

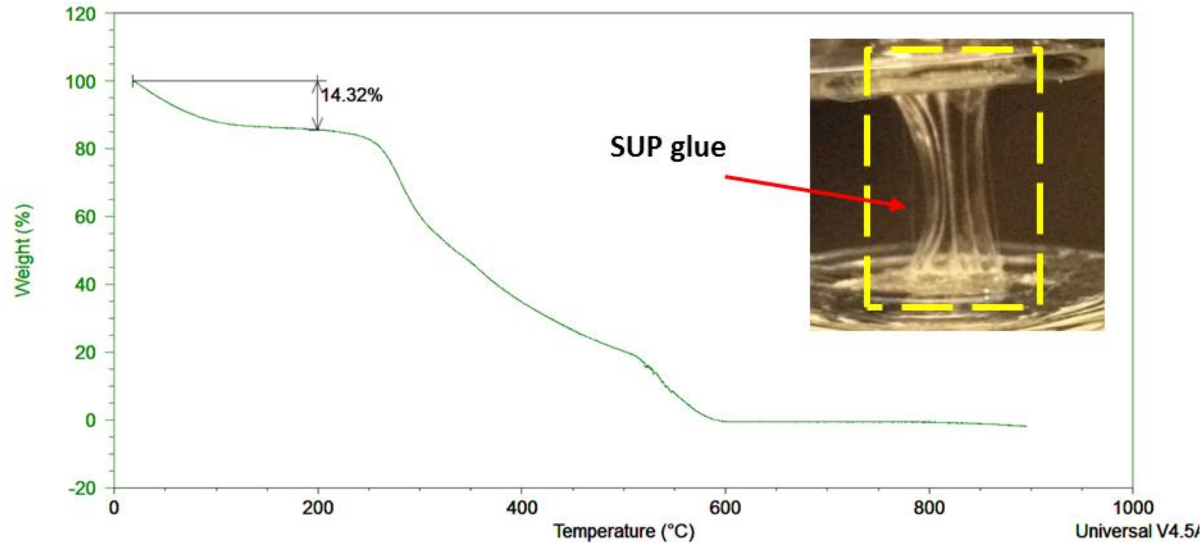

Supplementary Figure 9. TGA investigation of the K72-SDBS glue before lap shear testing. The measurement shows that ca.14% water are remaining in the SUP glue system. Inset represents the sticky behavior of SUP glue applied on two glass surfaces.

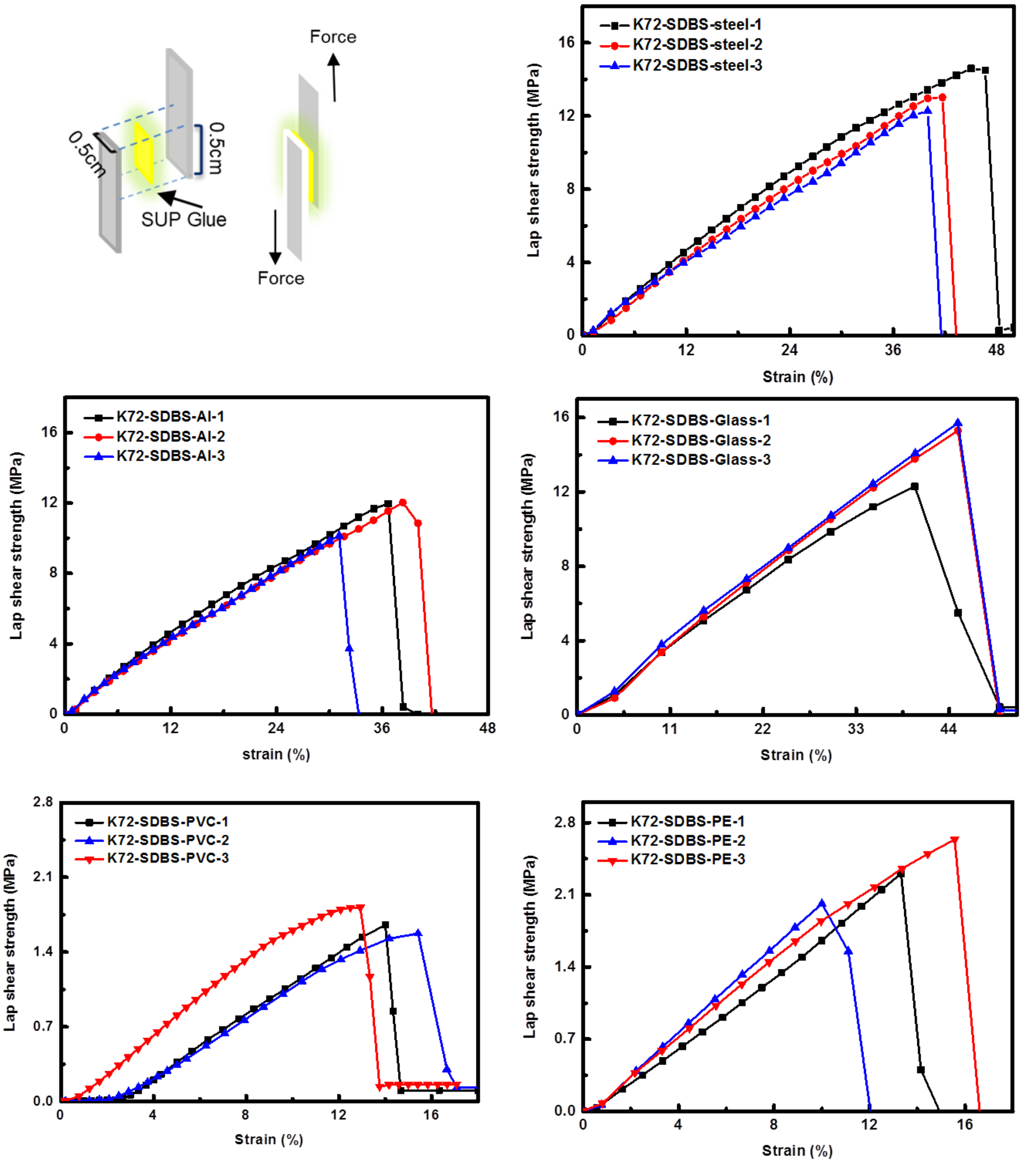

Supplementary Figure 10. Adhesion properties of SUP glue (here, K72-SDBS was taken as an example) quantified on different substrates, including steel, aluminum (AL), glass, polyethylene (PE) and polyvinyl chloride (PVC). Three individual tests were performed for each group.

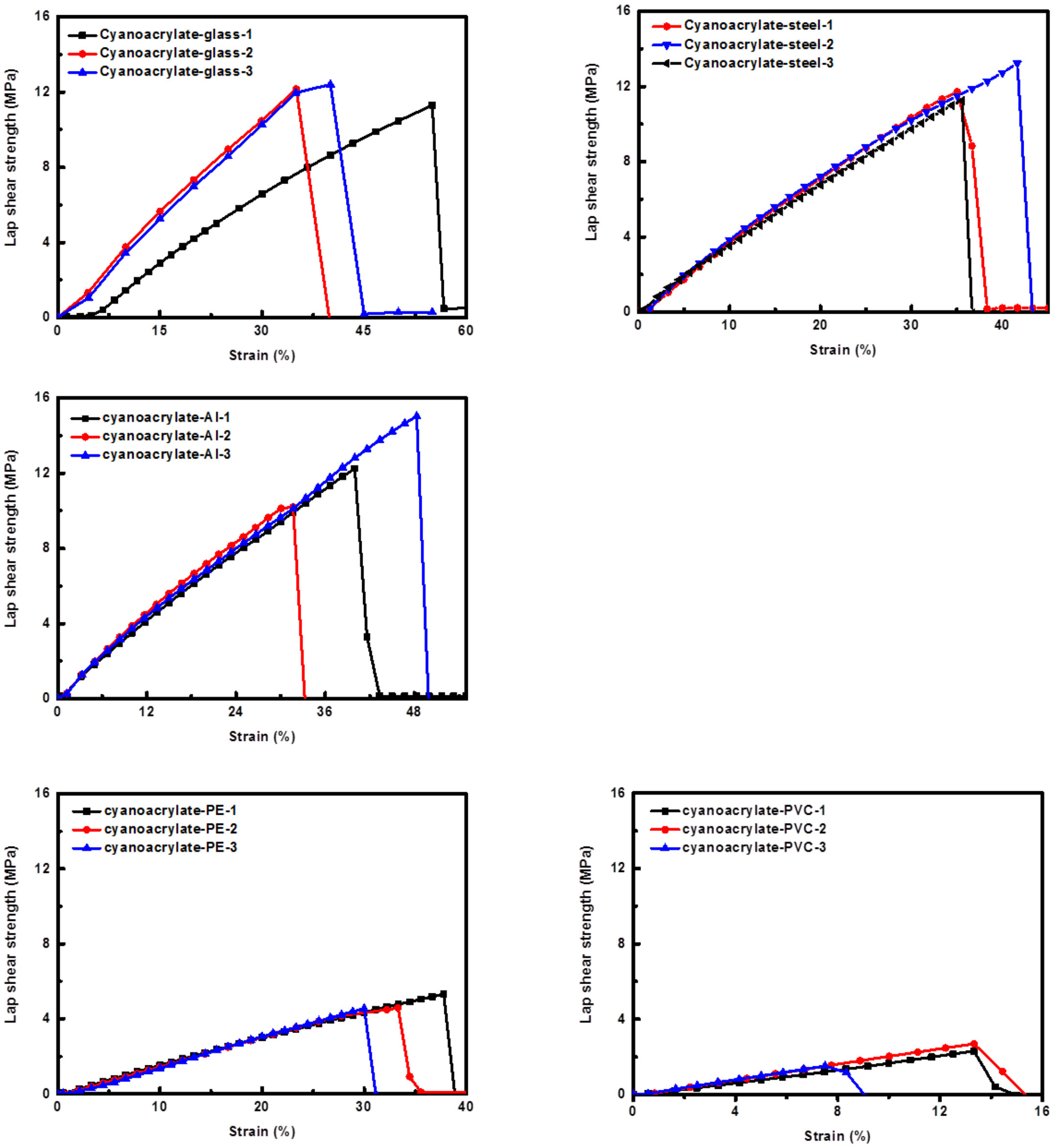

Supplementary Figure 11. Typical bulk characterization of cyanoacrylate glue on different substrates as control tests, indicating the comparable capacities of our SUP glue with the superglue cyanoacrylate products.[3]

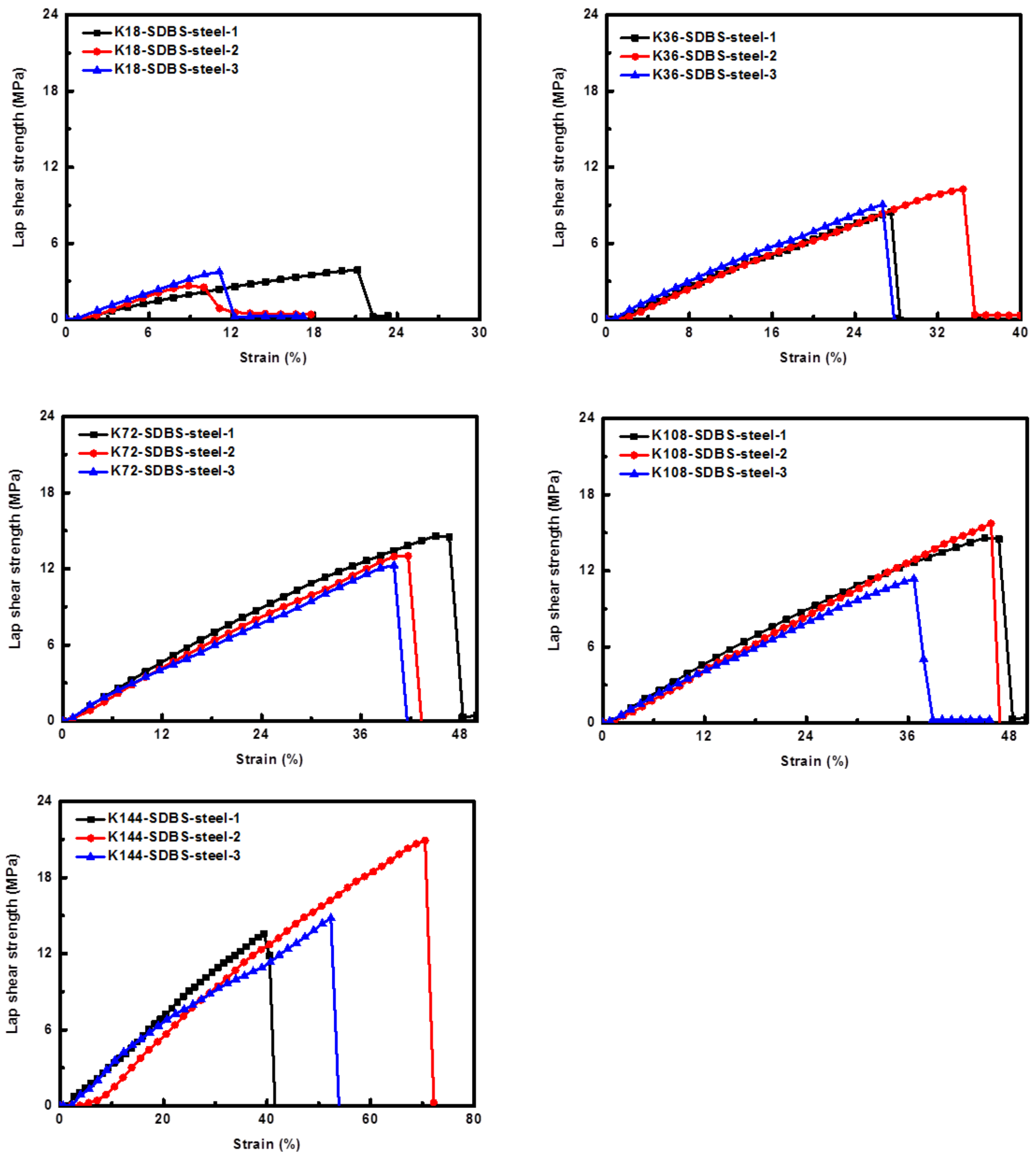

Supplementary Figure 12. Study of the influence of the molar mass of the SUP component of the glue on the adhesion behavior including K18, K36, K72, K108 and K144 variants. The tests were performed on steel substrates with three individual tests for each subtype.

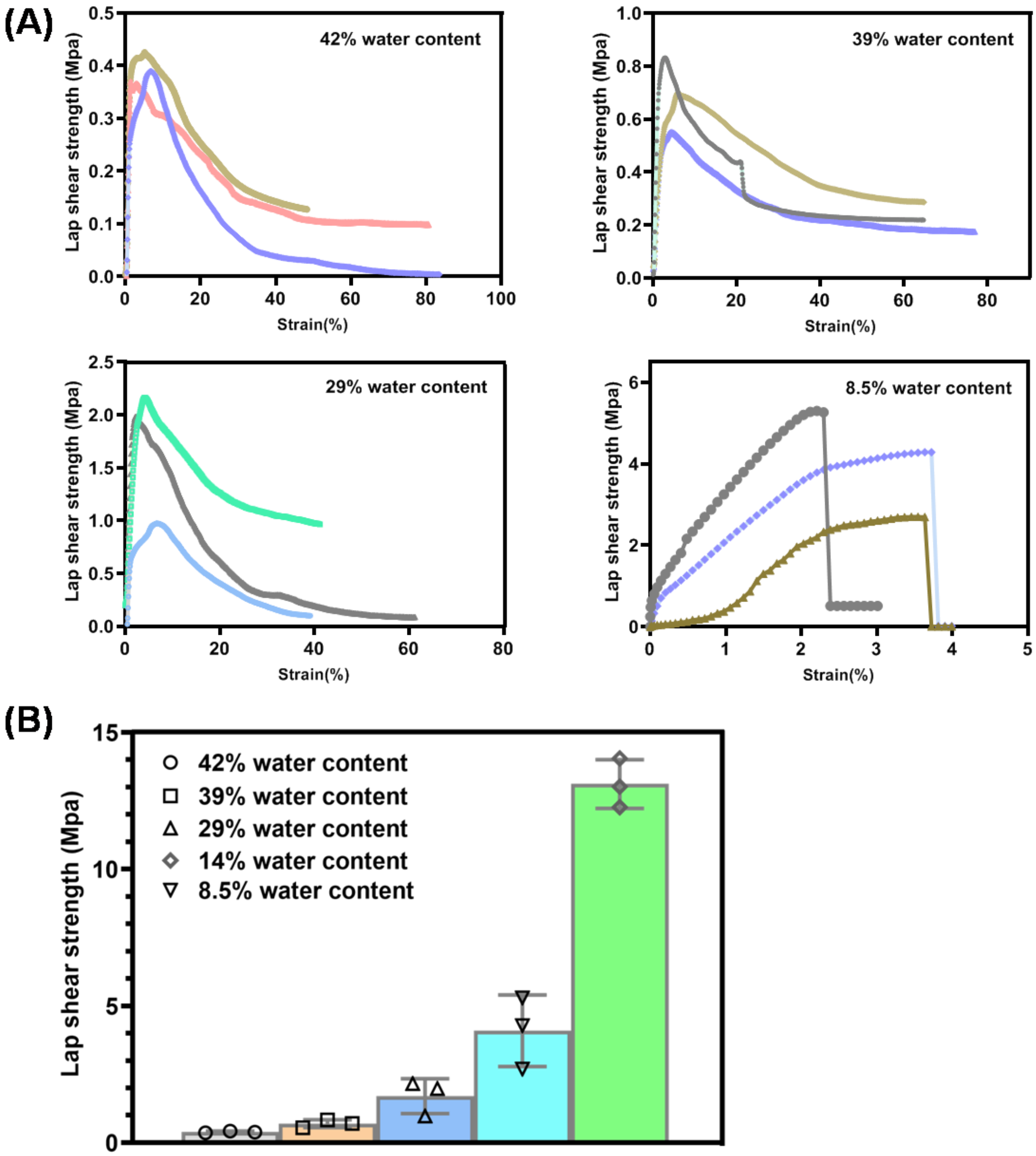

Supplementary Figure 13. Dry lap shearcharacterization on steelof K72-SDBS complexes with different water contents. (A) Original tensile curves recorded by the instrument with varying water contents ranging from 42% to 8.5%. (B) Compiled data showing a peak performance at 14% water content. The original stress-strain curves of the 14% test group are shown in Supplementary Figure 12. Data are mean values ± SD from the mean from N = 3 independent measurements on independent samples.

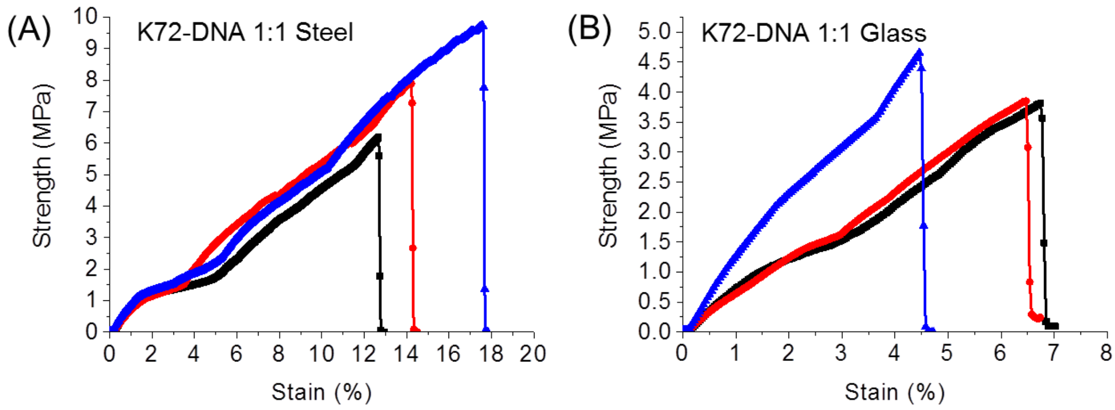

Supplementary Figure 14. Adhesion test of K72-DNA complexes on steel (A) and glass(B).

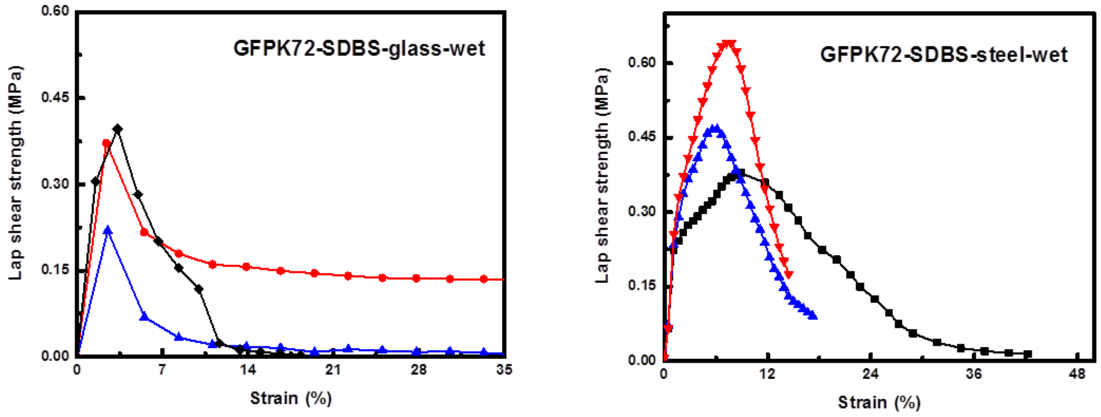

Supplementary Figure 15. Underwater adhesion tests of SUP glues (here GFPK72-SDBS was taken as an example). The quantification of adhesion strength revealed values in the range of hundreds of kilopascals.This range is comparable to other underwater bio-adhesives reported to date.[4,5] Two types of substrates (glass and steel) were selected and three individual measurementsfor each sample were carried out.

(A)

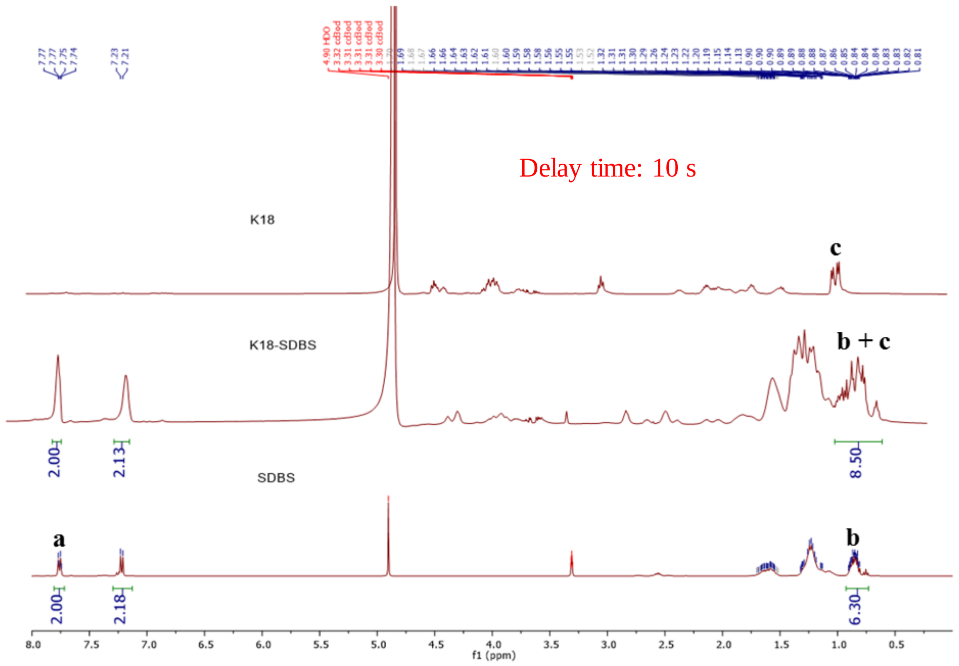
Delay time: 10 s
K18
c
K18-SDBS
b + c
2.00
2.13
8.50
SDBS
a
b
2.00
2.18
6.30
8.0 7.5 7.0 6.5 6.0 5.5 5.0 4.5 4.0 3.5 3.0 2.5 2.0 1.5 1.0 0.5
f1 (ppm)

(B)

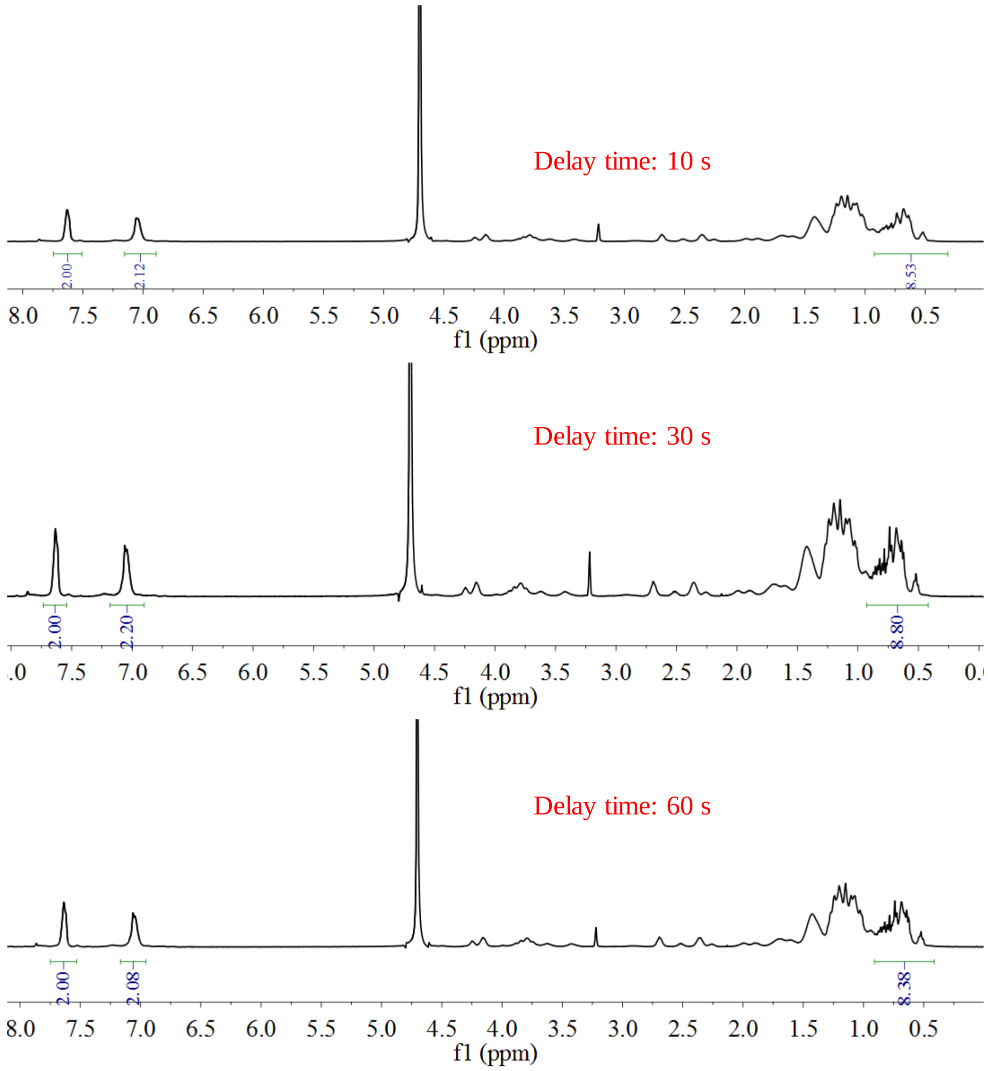
Delay time: 10 s
2.00
2.12
8.53
8.0 7.5 7.0 6.5 6.0 5.5 5.0 4.5 4.0 3.5 3.0 2.5 2.0 1.5 1.0 0.5
f1 (ppm)
Delay time: 30 s
2.00
2.20
8.80
7.5 7.0 6.5 6.0 5.5 5.0 4.5 4.0 3.5 3.0 2.5 2.0 1.5 1.0 0.5
f1 (ppm)
Delay time: 60 s
2.00
2.08
8.38
8.0 7.5 7.0 6.5 6.0 5.5 5.0 4.5 4.0 3.5 3.0 2.5 2.0 1.5 1.0 0.5
f1 (ppm)

(C)

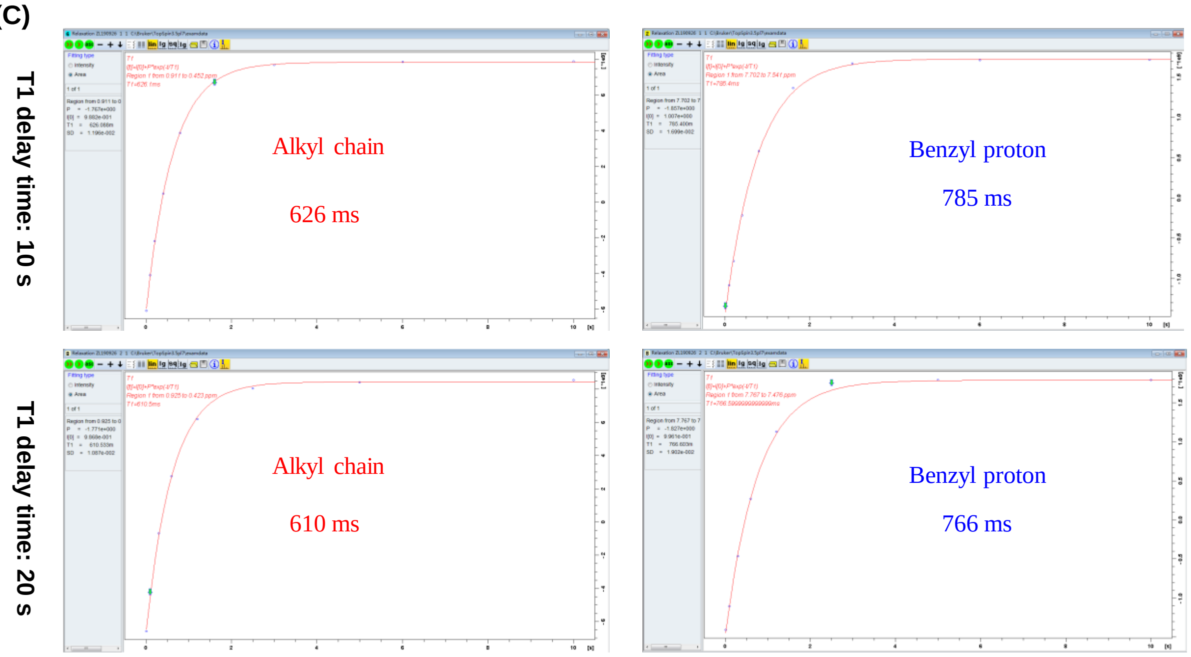

Supplementary Figure 16. (A) ^{1}H-NMR measurements of the SUP glue systemand the individual components (delay time: 10s). Here K18-SDBS adhesives with a molar ratio of lysine to surfactant of 1:5 was used as a representative example.Labelling of protons as described in paragraph 5. (B) To verify thereliability of the measurements, ^{1}H-NMR measurements of K18-SDBS complex (1:5) in $D_2O/CD_3OD$with different delay times were performed, including 10, 30, and 60 s. The results showed that there are no significant differencesfor measurements with different delay times. (C) The longitudinal relaxation time of the aromatic ring protons and alkyl chain protons were investigated for different T1 delay times. Here K18-SDBS (1:5) at complexation amounts of 10.0 mg K18 and 30.0 mg SDBS at 298 K in D_2O/CD_3OD was measured as a representative example.

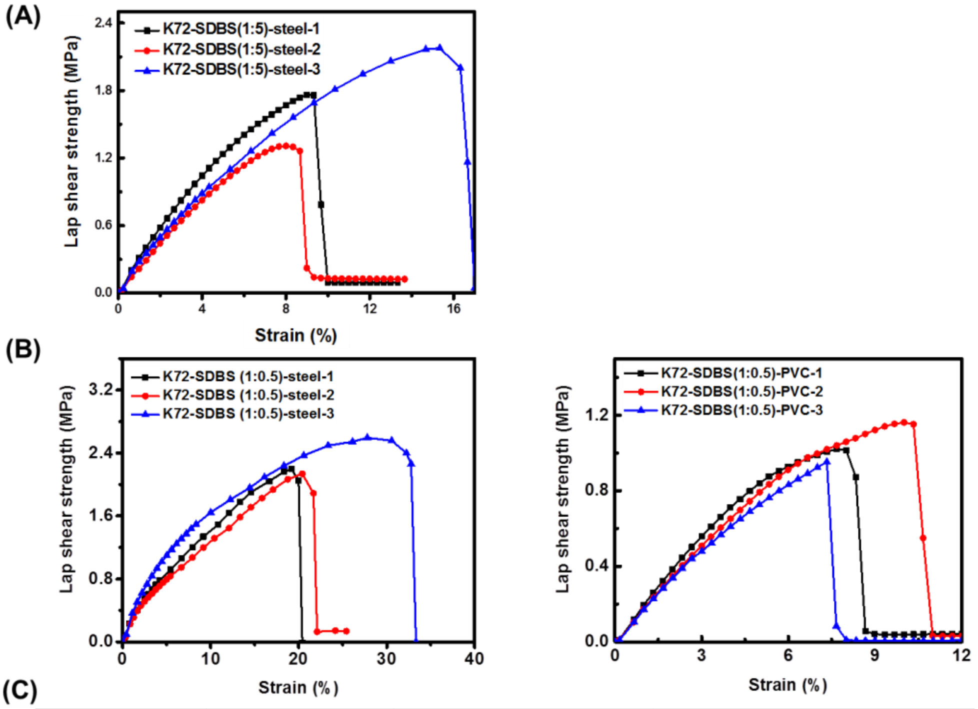

(C)

Adhesive	Substrate	Lap shear strength (MPa)	Adhesion energy (J/m^2)
K72-SDBS (1:5)	Steel	1.8 ± 0.4	995 ± 599
K72-SDBS (1:0.5)	Steel	2.0 ± 0.3	1795 ± 142
	PVC	1.0 ± 0.1	438 ± 124

Supplementary Figure 17. Adhesion characterization of K72-SDBS glue with different complexation ratios. (A) The performance on a steel surface with the glue prepared in a molar ratio of lysine to surfactant of 1:5. Compared to the K72-SDBS systemprepared with 1:1 molar ratio, the present sample exhibits significantly reduced adhesion performance.The adhesion strength declined from around 14 MPa to 1.8 MPa. (B) The adhesion performance of K72-SDBS glue prepared in a molar ratio of lysine to surfactantof 1:0.5 on steel and PVC surfaces. (C) Summary of the mechanical performance of the two types of K72-SDBS complexes.

Supplementary Table 3. Adhesion energies of cyanoacrylate and SUP glue on different surfaces. (A) Data presentation in a table. (B) Presentation of data in form of column chart. **p < 0.01. All presented data are mean values ± SD from the mean from N = 3 independent measurements on independent samples. All p-values were calculated using two-sided Student's t-test.

(**A**)

Adhesives	Substrates	Adhesion energy (J/m^2)
Cyanoacrylate	Glass	5107 ± 931
	Steel	5141 ± 930
	Al	5050 ±2042
	PE	2420 ± 470
	PVC	440 ± 1600
K72	Glass	6290 ± 1190
	Steel	5394 ± 470
	Al	4577 ± 176
	PE	510 ± 180
	PVC	1023 ± 118
K18	Steel	735 ± 448
K36	Steel	4523 ± 848
K108	Steel	7891 ± 2571
K144	Steel	10273 ± 3976
K72-recovered	PE	354 ± 61
GFP-K72 (wet)	Glass	20 ± 12
	Steel	50 ± 18

(**B**)

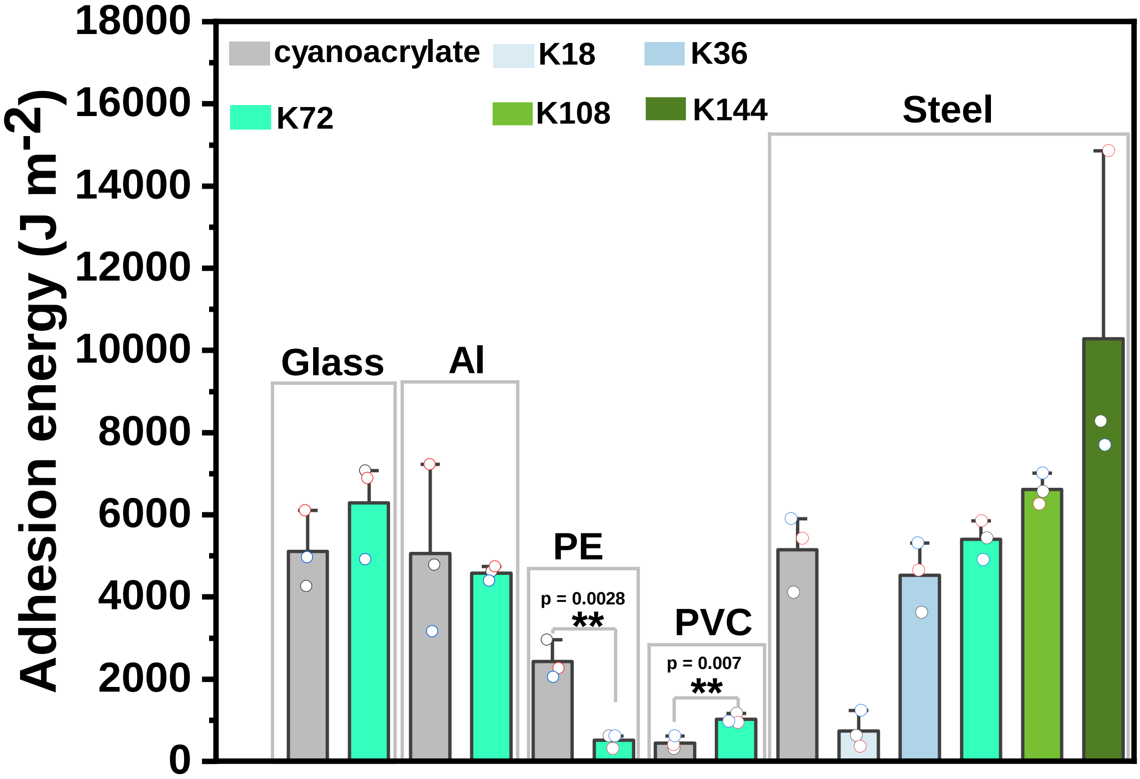

Adhesion energy (J m-2)
18000
16000
14000
12000
10000
8000
6000
4000
2000
0
cyanoacrylate
K18
K36
K72
K108
K144
Steel
Glass
Al
PE
p = 0.0028
**
PVC
p = 0.007
**

Supplementary Table 4. Overview of adhesion strengths of adhesives in the literature. Two classes of typical adhesives were compiled, including genetically engineered proteinaceous adhesives and chemically synthesized biomimetic adhesives.

adhesives		adhesion test	substrate	conditions	adhesion strength/energy	referennce
Genetic engineering protein-based adhesive	Mfp-CsgA	SFA	mica	wet	20.9 mJ/m^2	Nat. Nanotechnol., 2014, 9, 858
	mfp-1 functionalized film	SFA	mica	Fe^{3+}/underwater	~ 5.7 mJ/m^2	PNAS, 2010, 107, 12850
	rmfp-1/MADQUAT	SFA	mica	wet	~ 4.8 mJ/m^2	PNAS, 2016, 113, e847
	dfp-5	SFA	mica	underwater	~ 3.7 mJ/m^2	Angew. Chem. Int. Ed., 2014, 53, 13360
	Mfp-151, HA	lap shear	aluminum	dry	~ 3-4 MPa	Biomaterials 2010, 31, 3715
	WIMBA (mrfp/ HA)	lap shear	bladder tissue	underwater	~140 kPa	Biomaterials, 2015, 72, 104
	Rmfp-1	lap shear	aluminum	Fe3+, wet	~ 200 kPa	Biomacromolecules, 2014, 15, 1579
	rMaSp1/rMaSp2	lap shear	steel	dry	0.75 Mpa	Biomacromolecules, 2016, 17, 3761
	mELYs	lap shear	glass	dry	2.1 ± 0.5 Mpa	Biomaterials, 2017, 124, 116
		lap shear	glass	wet	~ 240 kPa	
	MeHA/ELP-ZnO	lap shear	glass	dry	0.5 Mpa	ACS Biomater. Sci. Eng., 2018, 4, 2528
Chemically synthetic biomimetic adhesive	PAAcat- QCS-Tf2N	SFA	glass	underwater	~ 2 J/m^2	Nat. Mater., 2016, 15, 407
	Functionalized Silicon Nanoparticles	lap shear	porcine liver	wet	6 ~ 25 J/m^2	Nature, 2014, 505, 382
	mfp-3s mimetic copolyampholytes	SFA	mica	wet	~ 32.9 mJ/m^2	J. Am. Chem. Soc., 2015, 137, 9214
	Catechol-functionalized zwitterionic coacervate	SFA	mica	wet	50 mJ/m^2	Nat. Communs. 2015, 6, 8663
	PEG-DOPA functionalized silk fibroin	lap shear	aluminum	$NaIO_4$	~ 130 kPa	Biomacromolecules, 2016, 17, 237
	PEG-DOPA-polylysine	lap shear	porcine skin	horseradish peroxidase/wet	~ 147 kPa	Adv. Funct. Mater., 2017, 27, 1604894
	poly[(3,4-dihydroxystyrene)-co-styrene]	lap shear	aluminum	dry	~ 11 MPa	Adv. Funct. Mater., 2014, 24, 3259
	Sandcastle worm mimetic coacervate/Mg2+	lap shear	aluminum	underwater	~ 600 kPa	Adv. Mater., 2010, 22, 729
	P(DMA-co-MEA)/PDMS	AFM	-	wet	86.3 ± 5 nN / pillar	Nature, 2007, 448, 338

6. Molecular Force Measurements using surface forces apparatus (SFA)

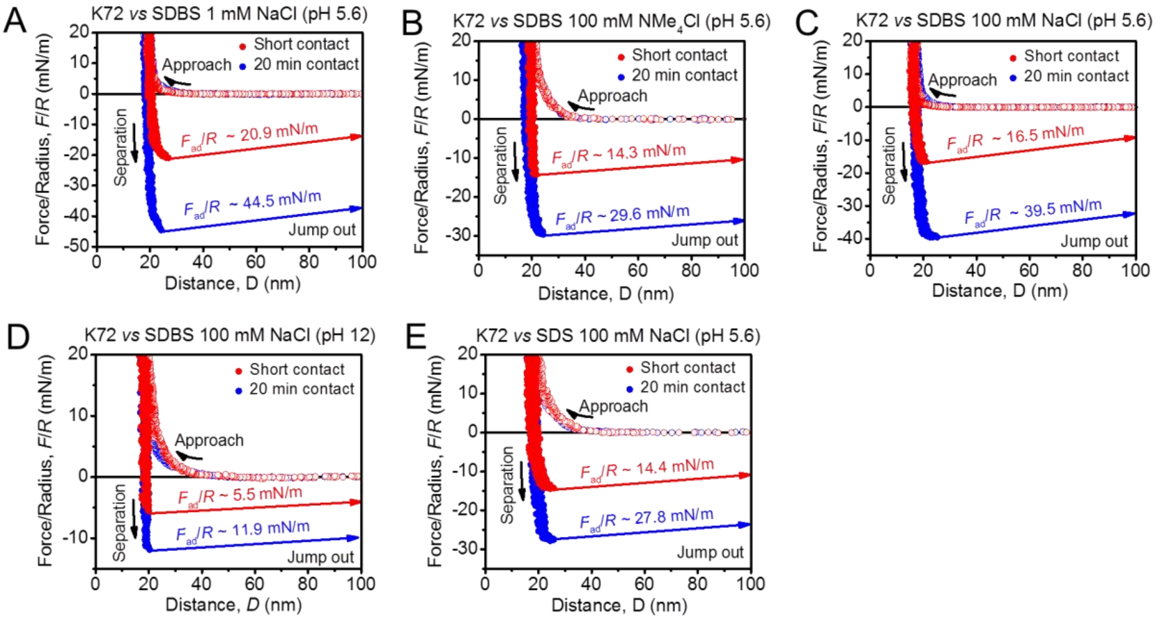

Supplementary Figure 18. Surface force apparatus (SFA) characterization offorce-distance profiles between K72 and SDBS layers, and between K72 and SDS layers in various aqueous media. (A) - (D) Impact of aqueous conditions on the interaction force curves between K72 and SDBS layers: (A) 1 mM NaCl and pH 5.6, (B) 100 mM NaCl and pH 5.6, (C) 100 mM NMe_4Cl and pH 5.6, and (D) 100 mM NaCl and pH 12. (E) Force-distance profiles measured between K72 and SDS layers in 100 mM NaCl at pH 5.6. The short contact refers to the contact time of 1 min.

7. Computer simulations of SUP-SDBS and SUP-SDS complexes

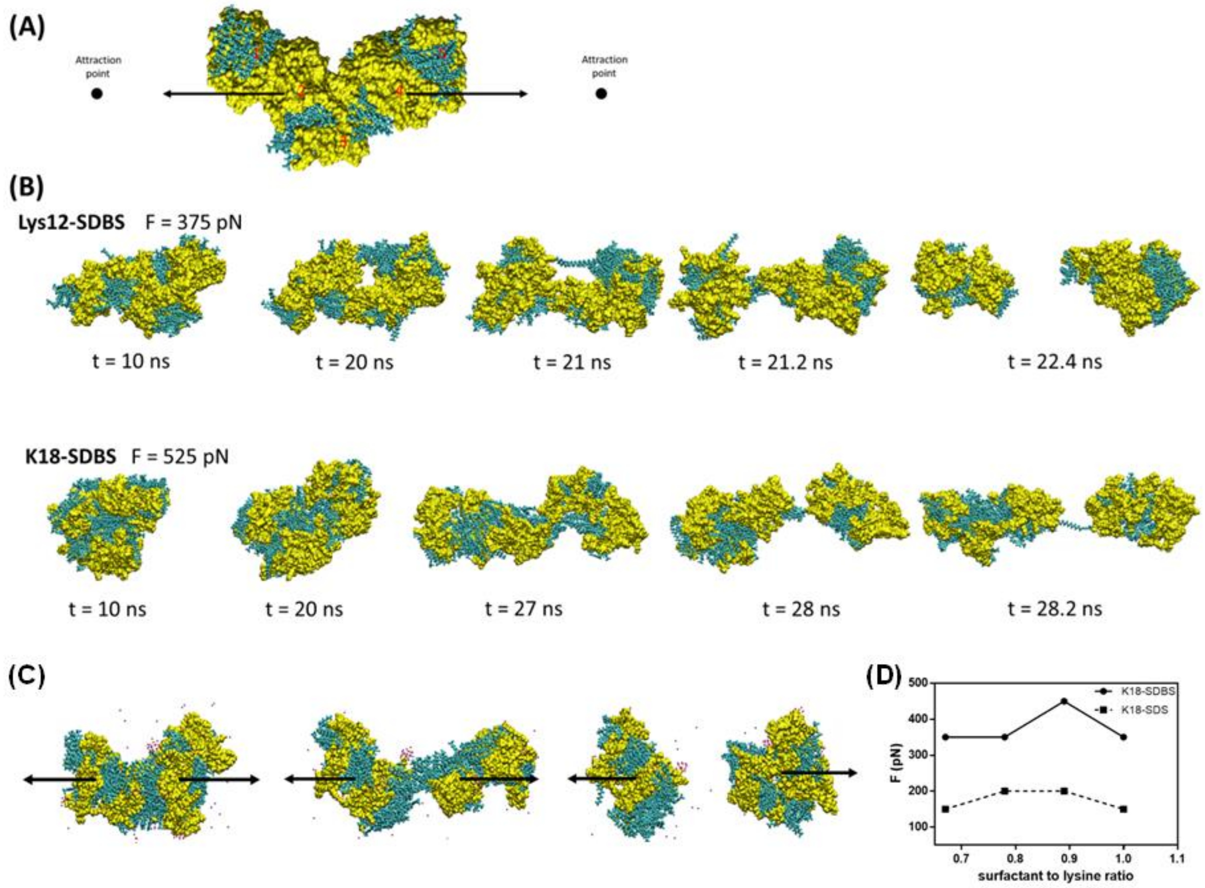

Supplementary Figure 19. Characterization of the splitting process of SUP-SDBS complexes. (A) Simulated splitting of K18-SDBS complexes by external force. Here, five molecules of K18 are complexed with stoichiometric amount of SDBS molecules. The opposite directions of forces are indicated. (B) Two more examples, *i.e.* Lys12-SDBS and K18-SDBS, are shown with a series of splitting snapshots. Maximum forces are indicated. (C) Three consecutive snapshots of the molecular simulation of disintegrating SUP-SDBS complexes under external force (indicated by black arrows): Here five K18 molecules are shown in yellow, SDBS in cyan, and sodium counter-ions as blue dots. (D) The computed data of applied forces under which the complex splits versus ratio of lysine to surfactant SDS (dashed line) and SDBS (solid line). When the ratio is 1:0.9, the force reaches a peak value in the K18-SDBS complex.

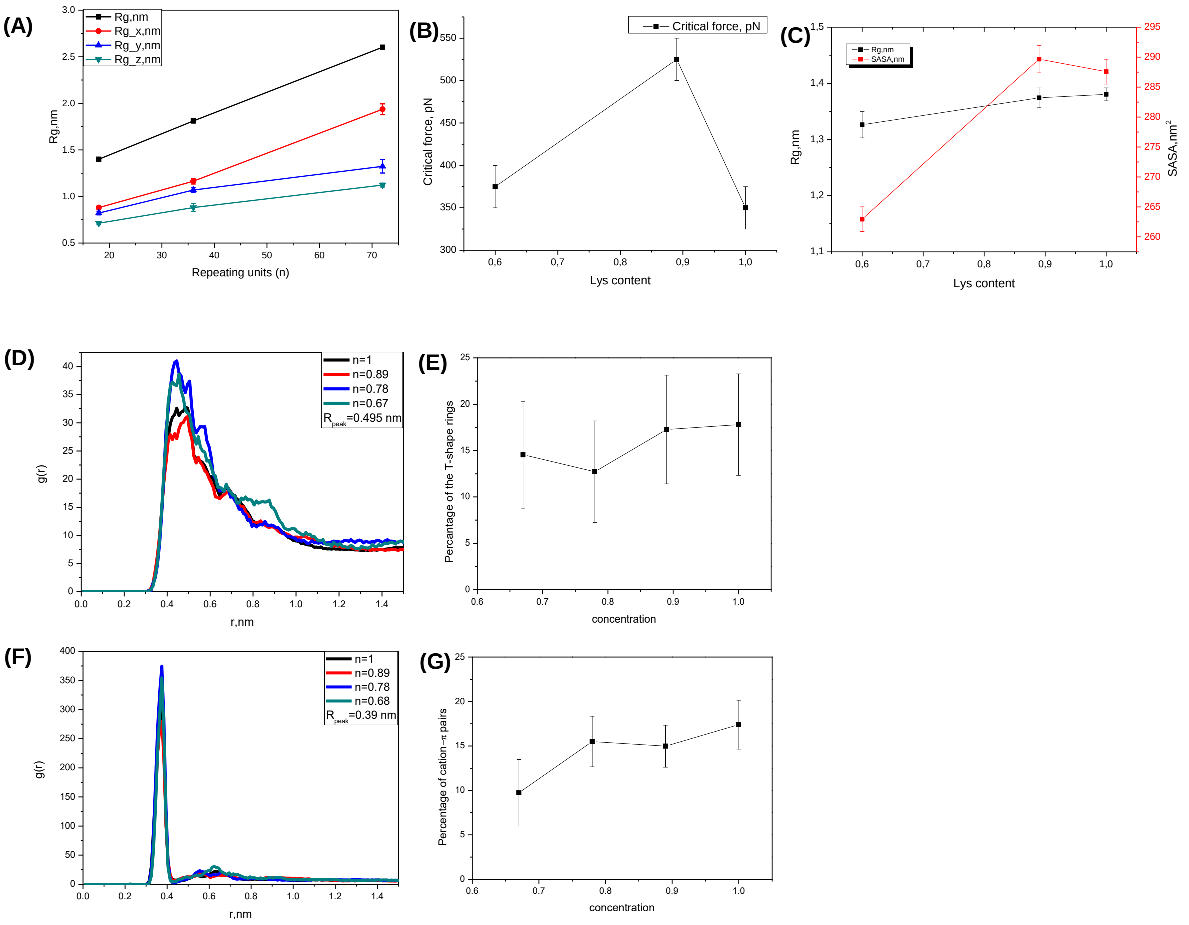

Supplementary Figure 20. The parameter profiles through molecular dynamic simulationsin the complex. (A) The x-(red), y-(blue) and z -(cyan) components of the average radius of gyration of the single peptides in the complex as a function of the number of repeating units (n). The total radius of gyration is shown by black dots. (B) The splitting force relative to Lys content.The lysine content is defined as the output of b/(a+b)in the formula [(GVG**V**P)$_a$(GVG**K**P)$_b$]$_n$, thus lysine content 0.6, 0.9 and 1.0 corresponds to Lys12, K18 and Lys20. The related peptide sequences are the following: Lys12 [(GVG**V**P)$_4$(GVG**K**P)$_6$]$_2$, K18 [(GVG**V**P)$_1$(GVG**K**P)$_9$]$_2$and Lys20 [(GVGVP)$_0$(GVG**K**P)$_{10}$]$_2$. (C) Parameters of different complexes involving Rg and solvent accessible surface area (SASA). The SASA for Lys12 is low because of its higher hydrophobicity compared to K18 and Lys20. (D) The radial distribution function g(r) between benzene rings. (E) The percentage of T-shape benzene pairs in SUP-SDBS complex with different surfactant molar ratio. (F) The radial distribution function g(r) between NH_3^+ - SO_3^-. (G) The percentage of cation-π pairs in SUP-SDBS complex with different surfactant molar ratios. All data in this figure are presented as mean values +/- SD with triplicate independent tests.

8. Biodegradability and recyclability of the SUP glue

Biodegradation behavior of the SUP glue

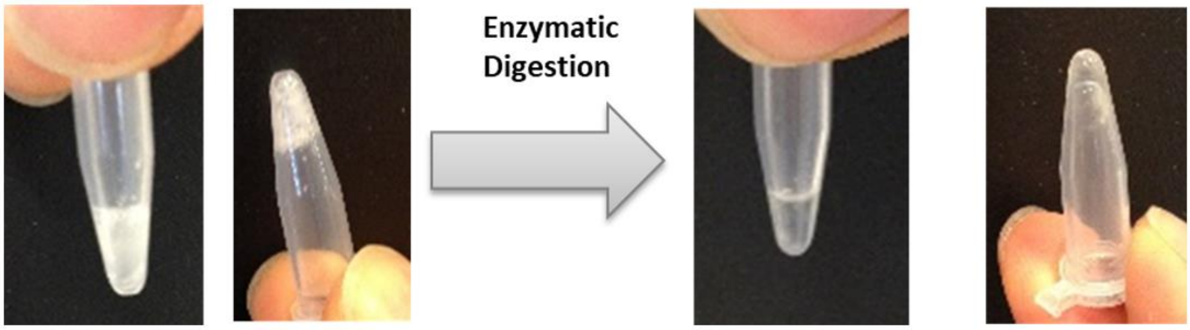

Supplementary Figure 21. Degradation experiment of the SUP glue (here K144-SDBS was taken as an example). Images on the left side show that the SUP glue prior to enzymatic treatment formed a gel with opaque appearance within theEppendorf vial. After the proteinase K digest, the SUP gluedispersion became transparent and formed a liquid as can be recognized when inverting the vial.

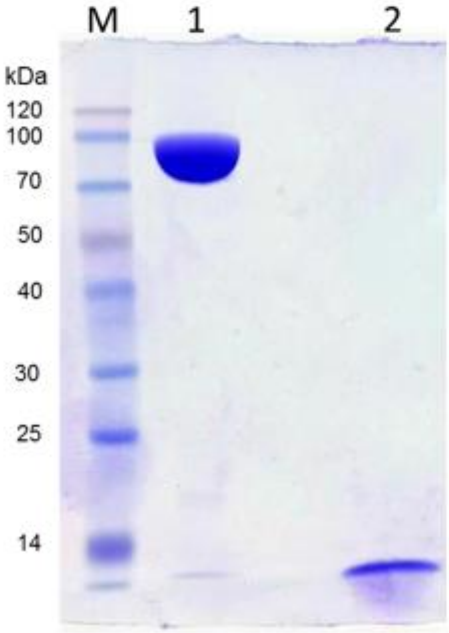

Supplementary Figure 22. Degradability test of the SUP glue (containing K144 as the polypeptide component). Lane1 representsrecombinant K144 expressed by *E. coli*. Lane 2shows the digestion products of the K144-SDBS complex treated with 0.1 mg·ml^{-1} ofelastasein PBS buffer at 37°C for 2h. M, prestained protein ladder. Three times each experiment were repeated independently with similar results.

Water cleanability of the SUP glue

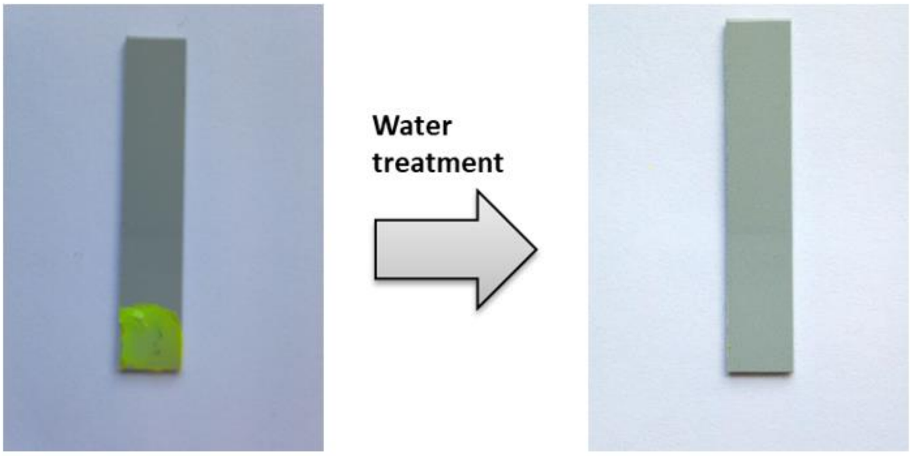

Supplementary Figure 23. The SUP glue (here GFP-K72-SDBS was taken as an example) can be easily cleanedby applying external forces such as harsh tap water or sonication treatment. The photographon the left represents a PE substrate pasted with GFP-K72-SDBS glue.After cleaning with water, the substrate shows a glue-free surface (right photograph).

Recyclability of the SUP glue

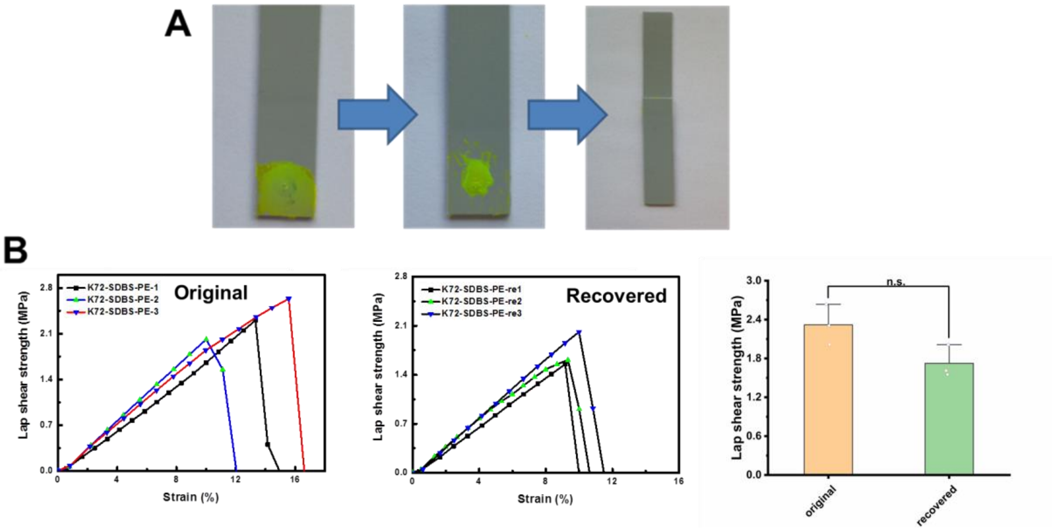

Supplementary Figure 24. The recyclability experiments of SUP glues. (A) The photographs show the SUP glue (GFP-K72-SDBS) on polyethylene (PE) that can be recovered by washing with excessive amount of H_2O and application of external force. Pure water is firstly applied on the surface of a fractured glue substrate. Subsequently, the glue can be collected when applying shearing force, as indicated in the middle. Thereafter, the recovered glue is re-applied on the surface of substrates for a second test (lap shear characterization). (B) Comparison between the original and recovered K72-SDBS glues on PE surface. The lap shear measurements indicate that the recovered glue sample is as strong as the original one. All presented data are mean values ± SD from the mean from N = 3 independent measurements on independent samples. All p-values were calculated using two-sided Student’s t-test.

9. Cytotoxicity Evaluation of the SUP Glue

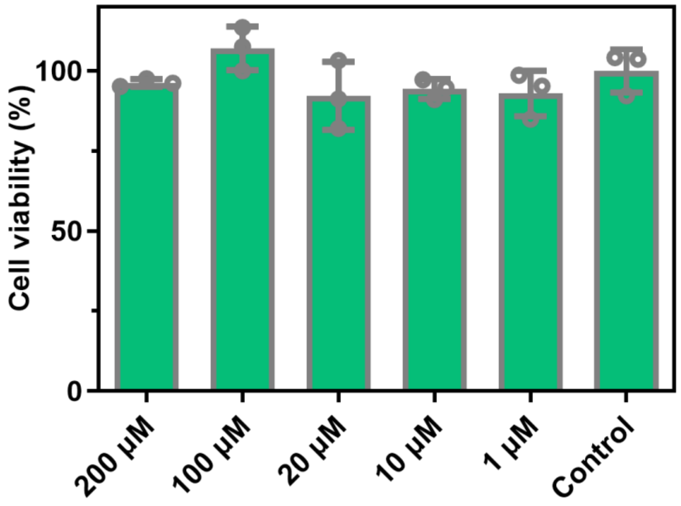

Supplementary Figure 25. Cell viability measurements carried out with different concentrations of K72-SDBS complex ranging from 1 to 200 μM using HeLa cells. The control group was cells treated with culture medium. All presented data are mean values ± SD from the mean from N = 3 independent measurements on independent samples.

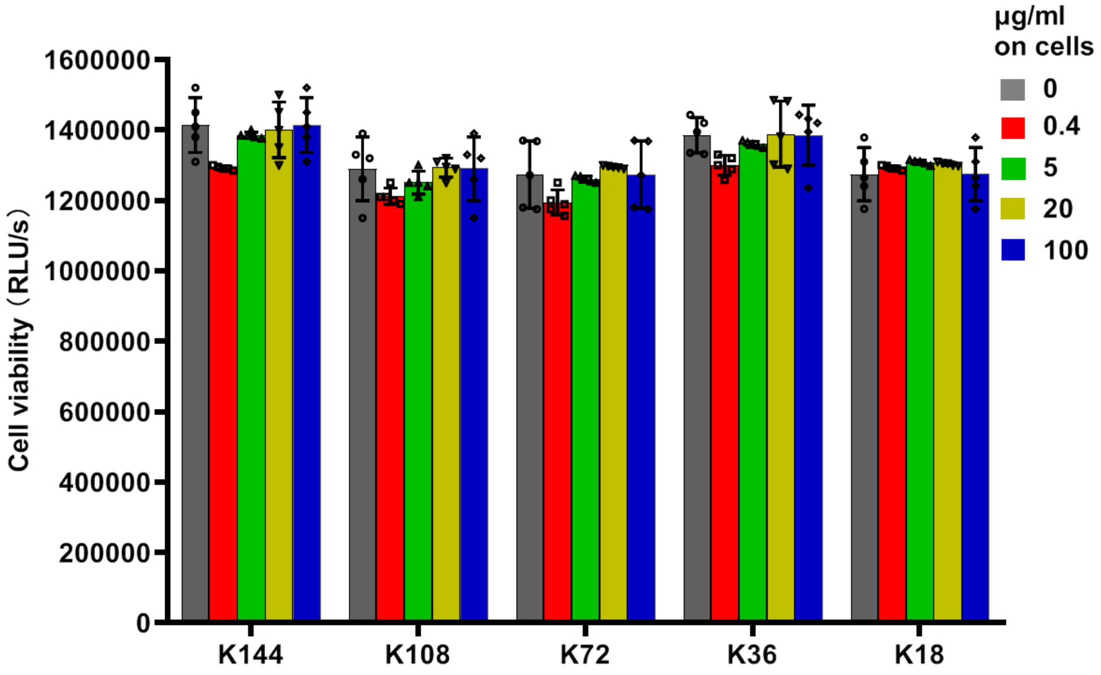

Supplementary Figure 26. Evaluation of cytotoxicity of the SUPs with HeLa cells. The measurements indicate that the cell viability is not affected by the addition of SUPs at a concentration as high as 100 μg·mL^{-1} (blue columns), which is also consistent with our previous investigation.[27] All presented data are mean values ± SD from the mean from N = 3 independent measurements on independent samples.

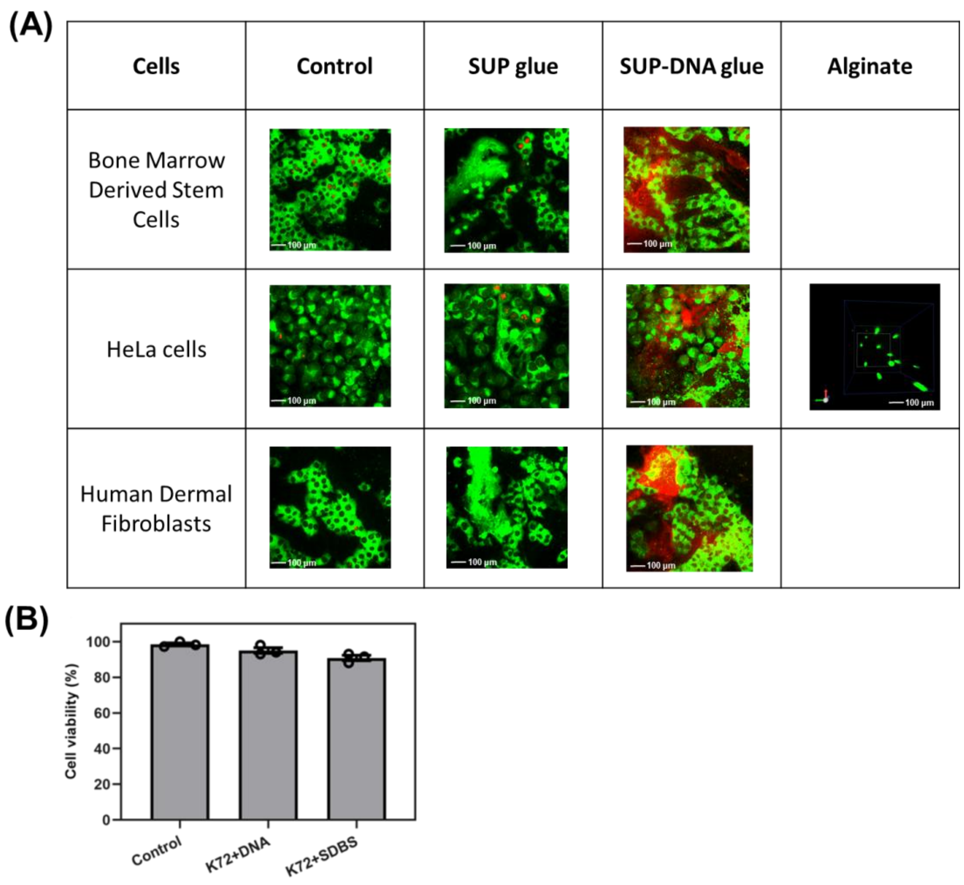

Supplementary Figure 27. (A) Encapsulation of various cells in the SUP glue, SUP-DNA glue or alginate hydrogel for 24 h in DMEM medium. The live/dead stain images were captured by confocal laser scanning microscopy. Alginate 3D culture was performed and the cells were loaded with the same densities as for the other groups. Three times each experiment were repeated independently with similar results. (B) Quantification of cell viability of the HeLa cells embedded in SUP glue and SUP-DNA glue via MTT. Important to note is that the PI dye also stains the non-cellular DNA. Thus, the background in SUP-DNA glue group appears in red to some extent due to the dye staining the salmon sperm DNA component of the glue. All presented data are mean values ± SD from the mean from N = 3 independent measurements on independent samples.

10. *Ex vivo* adhesion model tests on porcine skin and human eyelids

(A)

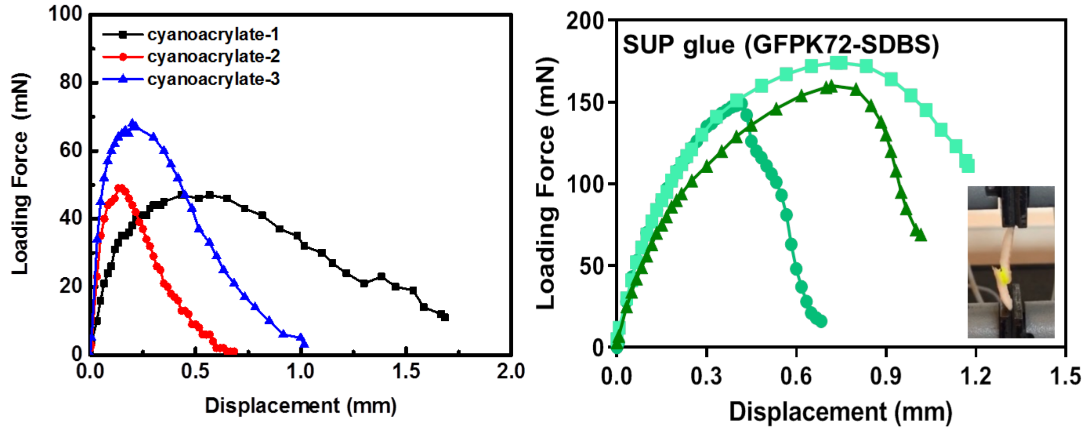

(B)

	W_{ad} (J/m²) 10mm/min	W_{ad} (J/m²) 50mm/min	W_{ad} (J/m²) 100mm/min	W_{ad} (J/m²) 600mm/min
Heart	10.8±0.2	77.8±16.9	154.4±30.6	260.3±114.3
Liver	7.2±2.3	14.4±2.3	17.4±4.8	45.3±14.8
Muscle	13.1±5.0	35.6±16.0	53.9±20.5	79.8±26.2

Supplementary Figure 28. Characterization of adhesion performance of SUP glue on wet tissue. (A) Adhesion test of cyanoacrylate and SUP glue on pig skin with a speed of 10 mm min^{-1}. Three curves were collected in lap shear measurements. (B) Overview of adhesion energy (W_{ad}) on various soft tissues. A series of stretching speeds was applied for the characterization of W_{ad} on the soft tissues.

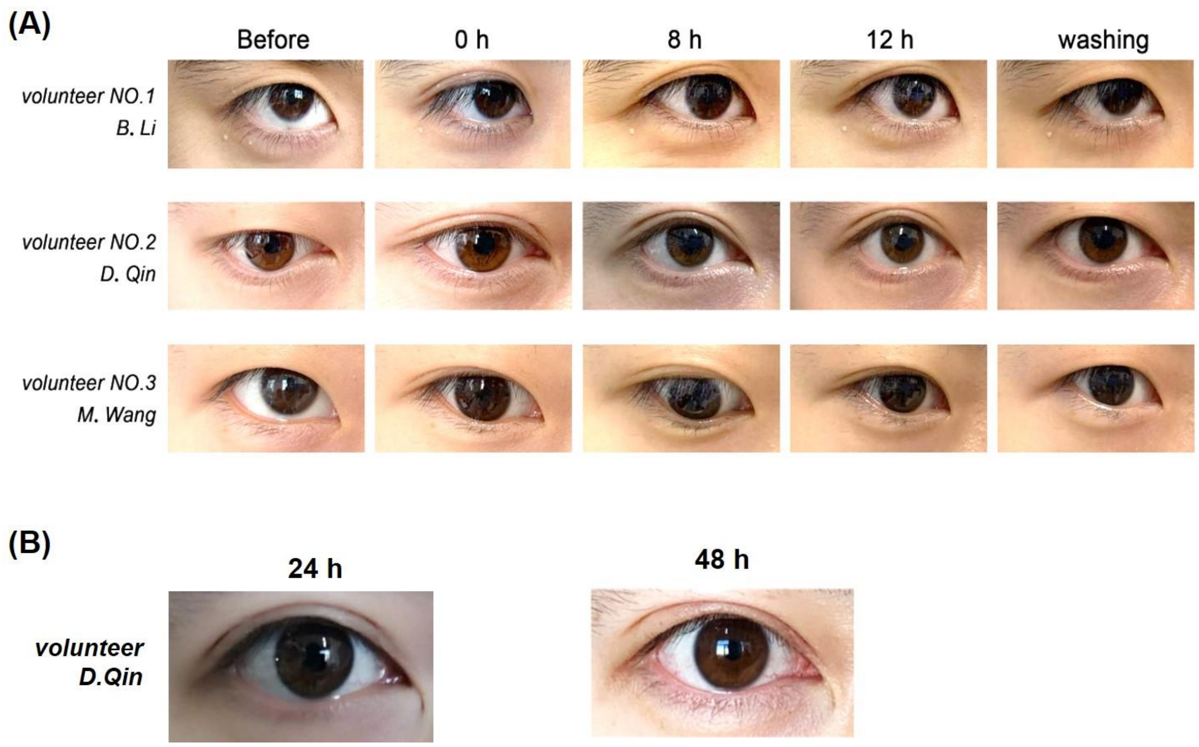

Supplementary Figure 29. Proof-of-concept demonstration of SUP glue as a new class of cosmetic adhesive. (A) Three volunteers were recruited (2 male with age of 25 or 27, 1 female with age of 23) to verify the viability and reproducibility of the glue system. The artificial double-eyelids persisted during daytime and were conveniently removed with tap water after 12 h. No extra cleansing lotion was needed. In contrast, conventional cosmetic glue products always require specific cleansing lotion, which typically contains organic solvents. (B) The adhesion performance of SUP glue persisted for as long as two days without any discernible reduction.

11. *In vivo* linear wound hemostasis and healing

Supplementary Table 5. Independent evaluation by clinical pathologists on histological slices of **linear** wounds healing.

(i). Blind scored by **Pathologist A**

Specimen	Skin repair degree	Inflammatory cell infiltration	Newborn capillaries	Collagen levels	Total Score	True Sample
a	1	2	3	3	9	SUP glue
b	3	3	3	3	12	SUP glue
c	3	1	3	1	8	SUP glue
d	1	2	1	3	7	no treatment
e	3	0	2	1	6	no treatment
f	1	0	1	1	3	suture
g	1	0	1	1	3	suture
h	3	1	2	2	8	medical glue
i	3	3	3	3	12	medical glue

(ii). Blind scored by **Pathologist B**

Specimen	Skin repair degree	Inflammatory cell infiltration	Newborn capillaries	Collagen levels	Total Score	True Sample
a	2	2	3	3	10	SUP glue
b	3	3	3	3	12	SUP glue
c	3	1	3	1	8	SUP glue
d	1	2	1	2	6	no treatment
e	3	0	1	1	5	no treatment
f	1	0	1	1	3	suture
g	1	0	1	1	3	suture
h	2	1	2	2	7	medical glue
i	3	3	3	3	12	medical glue

(iii). Blind scored by **Pathologist C**

Specimen	Skin repair degree	Inflammatory cell infiltration	Newborn capillaries	Collagen levels	Total Score	True Sample
a	2	2	3	3	10	SUP glue
b	3	3	3	3	12	SUP glue
c	3	1	3	1	8	SUP glue
d	1	2	1	3	7	no treatment
e	3	0	2	1	6	no treatment
f	1	0	1	1	3	suture
g	1	0	1	1	3	suture
h	2	1	2	2	7	medical glue
i	3	3	3	3	12	medical glue

Note: We gratefully thank Prof. Dr. Lihong Zhang (pathologist **A,** The Key Laboratory of Pathobiology, Ministry of Education, College of Basic Medical Sciences Jilin University, Changchun 130021, China), Prof. Dr. Shirui Liu (pathologist **B,** Department of dermatology, The Second Hospital of Jilin University, Changchun, Jilin 130041, China) and Prof. Dr. Hao Ming (pathologist **C,** Department of Gynecology, The Second Hospital of Jilin University, Changchun, Jilin 130041, China) for their great efforts on the evaluation of specimen slides. Here the SUP glue specimens were from K72-SDBS groups.

The details on the scoring are shown below,

Skin repair degree:

0. Tissue necrosis or suppuration around the incision

1. The wound is not completely healed, and a gap in the dermis

2. Wound is healed, but the interface is below or above the surrounding skin

3. Wound surface is smoothing

Inflammatory cell infiltration:

0.There is a large number of inflammatory cells infiltrating near the wound

1.There is moderate inflammatory cell infiltration near the wound

2. There is a small amount of inflammatory cell infiltration near the wound, but the degree of infiltration is higher than the distal skin tissue.

3. There is no difference in the degree of inflammatory cell infiltration around the wound and the distal skin tissue

Newborn capillaries:

0. There is no neovascularity near the wound

1. A small amount of new blood vessels appear near the wound

2. A large number of new capillaries appear near the wound

3. The mount of capillaries near the wound are richer than in the distal skin tissue

Collagen levels near the wound:

0. No obvious neonatal collagen near the wound

1. The new collagen near the wound is lightly colored.

2. The new collagen near the wound is dark colored, and the collagen fibers are loosely arranged.

3. The collagen is rich near the wound, and the collagen fibers are densely arranged

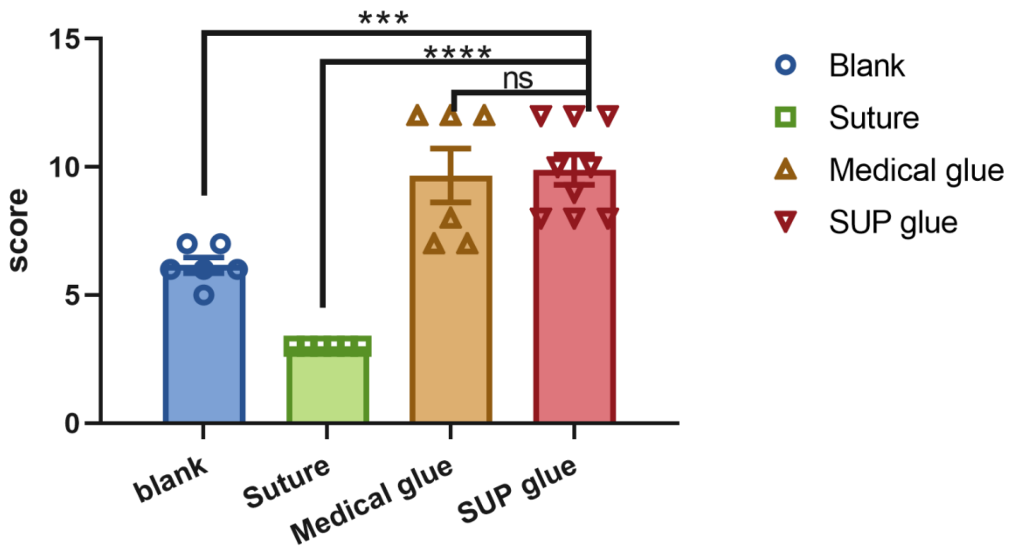

Supplementary Figure 30. Statistical analysis using two-sided Student's t-test on the independent immunohistochemistry (IHC) scoring of linear wound healing (N = 6 independent biological samples). ***, p = 0.0003; ****, p = 0.00000035; ns, not significant. Data are presented as mean values ± SD from the mean.

12. Wound hemostasis in tiny pig model

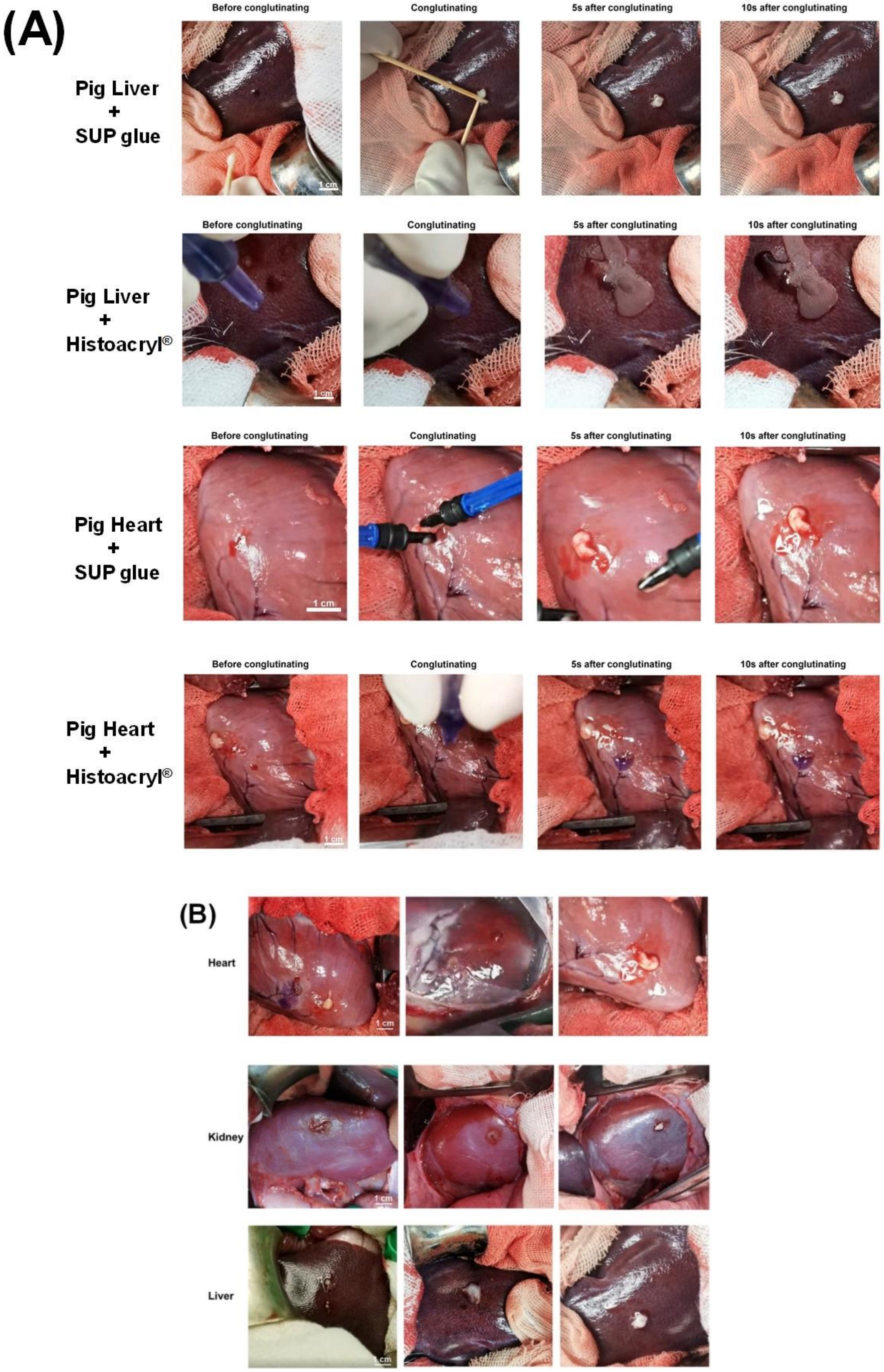

(C)

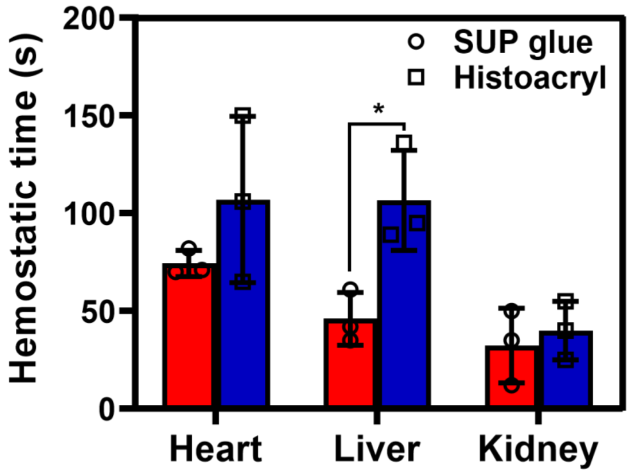

Supplementary Figure 31. (A) *In vivo* adhesion demonstration of SUP glue and commercial adhesive Histoacryl® on pig liver and heart models. The major functional component of Histoacryl® is cyanoacrylate. SUP glue shows excellent hemostasis effect due to robust adhesion performance and flexible deformation behavior. In contrast, the Histoacryl® solidified very fast when applied on the wounds, leading to leaking behavior on certain organ surfaces particularly the liver as shown here. (B) *In vivo* adhesion demonstration of SUP glue and commercial adhesive Histoacryl® on different pig liver, kidney and heart models (N = 3 biologically independent samples). (C) The quantification of hemostatic performance in terms of coagulation time in wounded areasapplied with SUP glue and Histoacryl® on different tissues. Statistical analysis was carried out employing two-sided student's t-test (N = 3 independent biological samples); *, p =0.0220. Data are presented as mean values ± SD from the mean. The results showed a significant enhancement of the performance for the liver tissue sealed with SUP glue compared to Histoacryl®. Due to various treatments on heart and kidney (artery occlusion and pre-clotting) before glue application, there is no observable difference between SUP glue and Histoacryl® in heart and kidney models.

13. *In vivo* round-shape wound dressing test

Supplementary Table 6. Independent evaluation by clinical pathologists on histological slides of **round-shape** wounds healing.

(i). Blind scored by **Pathologist A**

Specimen	Skin repair degree	Inflammatory cell infiltration	Newborn capillaries	Collagen levels	Total Score	True Sample
a	3	2	3	2	10	SUP glue
b	3	3	3	3	12	SUP glue
c	2	3	3	1	9	SUP glue
d	1	1	2	2	6	no treatment
e	2	2	3	2	9	no treatment
f	3	1	1	1	6	Saline
g	3	2	3	3	11	Saline
h	/	/	/	3	3	Cyanoacrylate
i	3	3	3	3	12	Cyanoacrylate

(ii). Blind scored by **Pathologist B**

Specimen	Skin repair degree	Inflammatory cell infiltration	Newborn capillaries	Collagen levels	Total Score	True Sample
a	3	2	3	2	10	SUP glue
b	3	3	3	3	12	SUP glue
c	2	2	3	1	8	SUP glue
d	1	1	2	3	7	no treatment
e	2	3	3	3	11	no treatment
f	1	1	1	1	4	Saline
g	3	2	3	3	11	Saline
h	/	/	/	3	3	Cyanoacrylate
i	3	3	3	3	12	Cyanoacrylate

(iii). Blind scored by **Pathologist C**

Specimen	Skin repair degree	Inflammatory cell infiltration	Newborn capillaries	Collagen levels	Total Score	True Sample
a	3	2	3	2	10	SUP glue
b	3	3	3	3	12	SUP glue
c	2	3	3	1	9	SUP glue
d	1	1	2	3	7	no treatment
e	2	2	3	2	9	no treatment
f	1	1	1	1	4	Saline
g	3	2	3	3	11	Saline
h	/	/	/	3	3	Cyanoacrylate
i	3	3	3	3	12	Cyanoacrylate

Note: Here the SUP glue specimens were from K144-SDBS groups. '/' indicates specific information in that specimen slide was missing and thereby cannot be evaluated with a score.

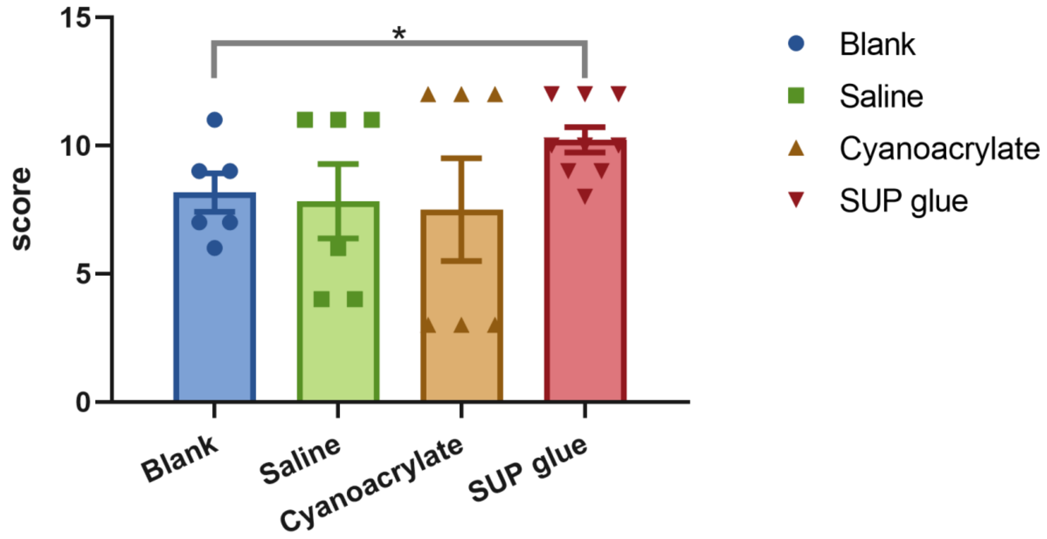

Supplementary Figure 32. Statistical analysis using two-sided Student's t-test on the independent IHC scoring of round-shape wound dressing (N = 6 independent biological samples). *, p =0.0322. Data are presented as mean values ± SD from the mean.

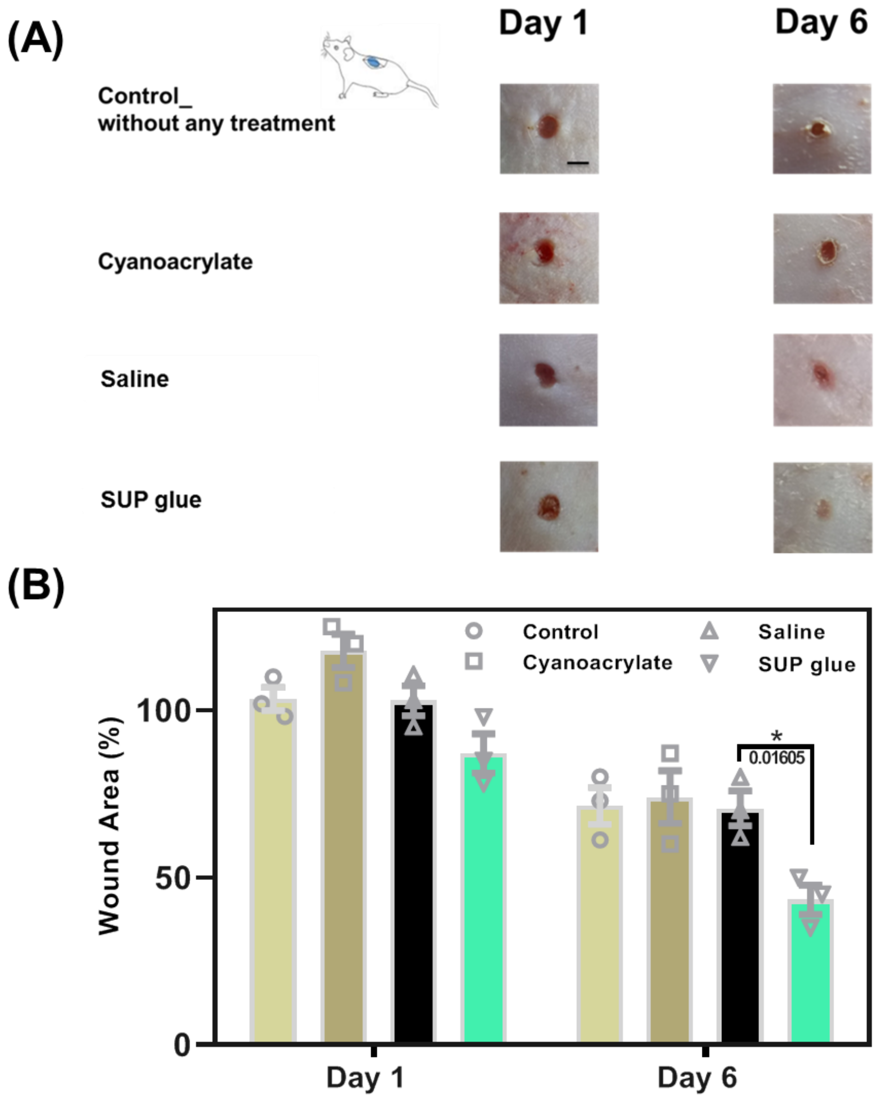

Supplementary Figure 33. Overview of *in vivo* wound dressing effects with different treatments. (A) Representative results of wound dressing on rats using different glue materials, including no treatment group, cyanoacrylate group, saline group as well as SUP glue (K144-SDBS) group. In the time course of six days, SUP glue actively accelerates the healing of round-shaped wounds. The experiments were independently performed in triplicate (N = 3 independent biological samples) for each group. Scale bar: 10 mm. (B) The quantification of wounded areas in the course of curing. The SUP glue group has a significant difference compared with the control groups. The statistical analysis was performed with two-sided Student's t-test (N = 3 independent biological samples). * p = 0.015. Data are presented as mean values ± SD from the mean.

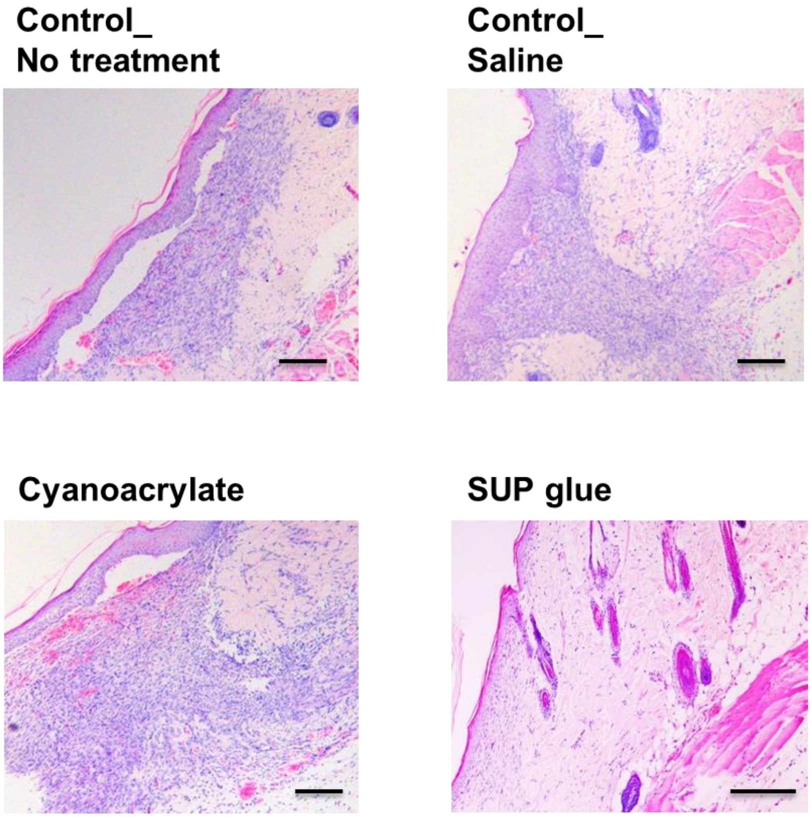

Supplementary Figure 34. Histological analysis (hematoxylin and eosin stain, H&E stain) of round-shaped woundssealed with different dressing agents. There are some new blood vessels and abundant granulation of tissue in the SUP glue (K72-SDBS) group, indicating good wound repair and skin regeneration. Three times each experiment was repeated independently with similar results. Scale bar: 100 μm.

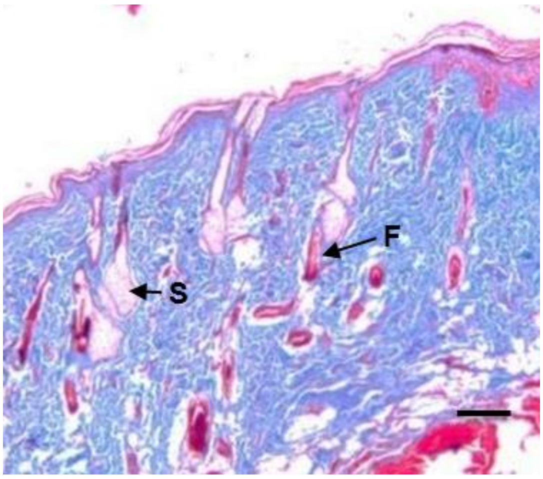

Supplementary Figure 35. Histological analysis (Masson's Trichrome stain) of round-shaped wounds sealed with dressing agent K72-SDBS glue. There was abundant,mature and compact collagen content (blue regions) in the group of SUP adhesive. F, follicle; S, sebaceous gland. Three times each experiment was repeated independently with similar results. Scale bar: 100 μm.

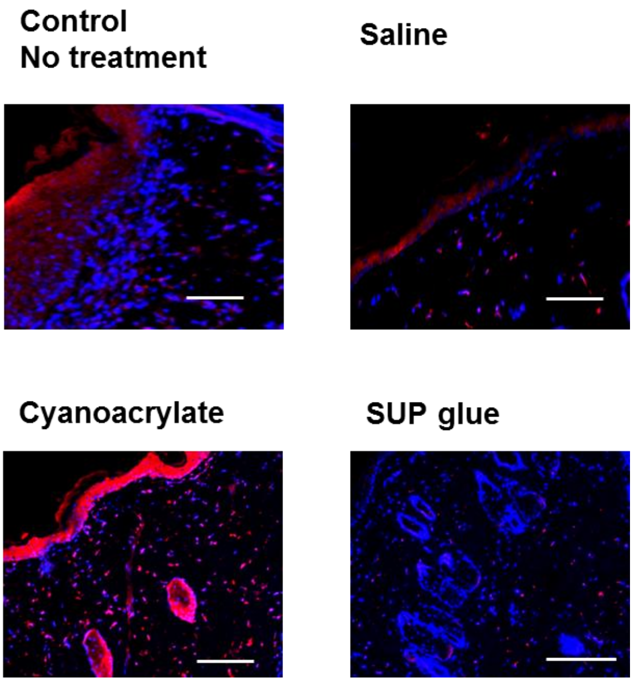

Supplementary Figure 36. Red immunofluorescence as indicator of the level of the pro-inflammatory cytokine IL-6. There was much less IL-6 expressed in the group treated with the K72-SDBS glue compared to the other samples.This is indicative for only few signs of inflammationas compared with the other control groups. Three times each experiment was repeated independently with similar results. Scale bar: 100 μm.

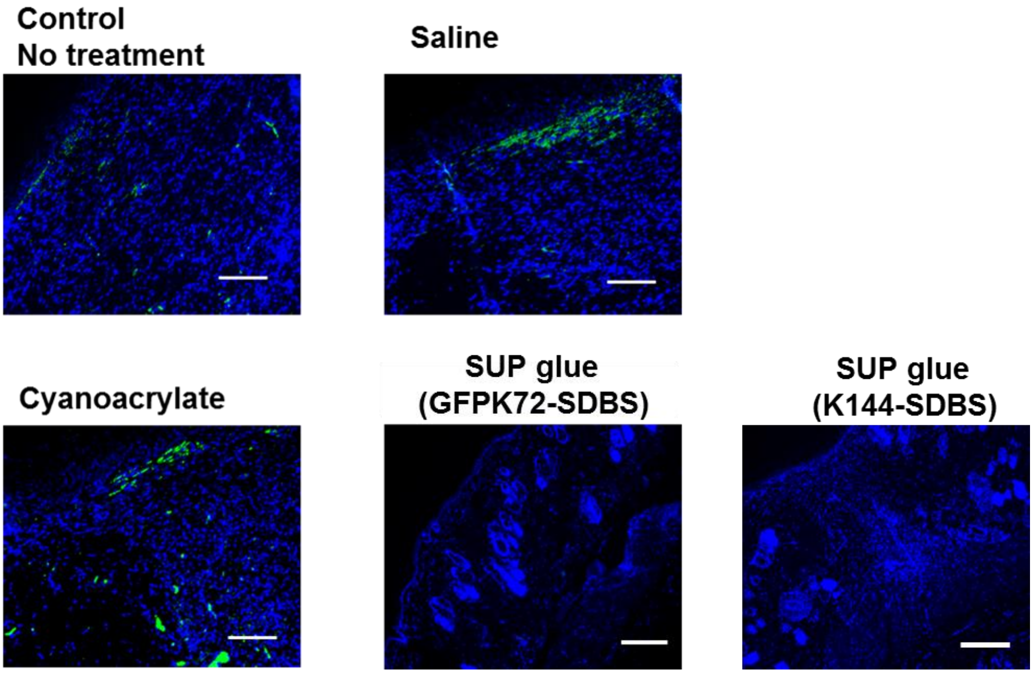

Supplementary Figure 37. Characterization of the level of pro-inflammatory factor TNF-α (in green). The results not only indicate the anti-inflammatory effect of SUP glue (here GFP-K72-SDBS and K144-SDBS were taken as examples), but also suggestits good biodegradability due to almost non GFP signal detected in the SUP glue test groups. Three times each experiment was repeated independently with similar results. Scale bar: 100 μm.

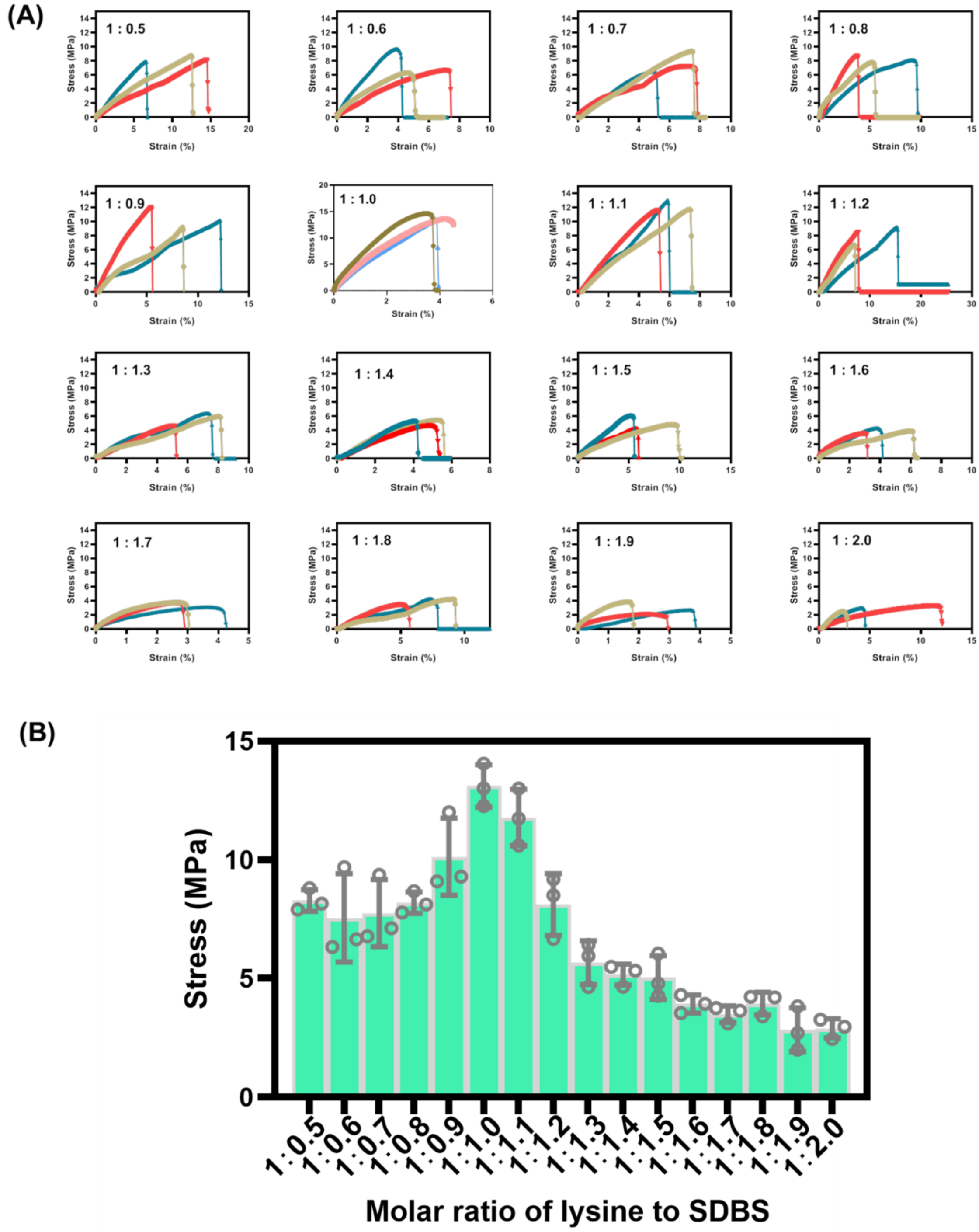

Supplementary Figure 38. Systematic investigation of the relationship between mechanical stress and molar ratio of lysine to SDBS in SUP glue systems. K144-SDBS complex was employed for this experiment. The molar ratios are from 1:0.5 to 1:2 with 0.1 intervals. Notably, the molar ratio indicated here is the initial ratio in the process of sample preparation.All mechanical characterization profiles are presented in (A). In addition, the data is compiled in a table format for better visuliaztion (B). All presented data are mean values ± SD from the mean from N = 3 independent measurements on independent samples for each subgroup.
