# Supplementary material for: Ultra-strong bio-glue from genetically engineered polypeptides
Source: Nat Commun. 2021 Jun 14;12:3613. doi: 10.1038/s41467-021-23117-9 (PMC8203747; doi:10.1038/s41467-021-23117-9)
Supplement: Supplementary file 3 — Description of Additional Supplementary Files [file 41467_2021_23117_MOESM3_ESM.docx]

**Description of Additional Supplementary Files**

**Supplementary Movie 1:** The molecular simulation of disintegrating of SUP-SDBS complex under external force. Five K18 molecules are shown in yellow, SDBS in cyan, and sodium counter-ions as blue dots.

**Supplementary Movie 2:** A mechanical test on two pieces of porcine skin glued together by SUP glue. Uniaxial extension characterization was performed by recording the corresponding force-extension curves. GFP-tagged SUP glue (GFP-K72-SDBS) was used for better tracking.

**Supplementary Movie 3:** *In vivo* adhesion on rat liver. The SUP glue (K72-SDBS) exhibits tissue adhering and hemostatic properties internally on a bleeding rat liver. The glue was transferred from the tip of a stick to the site of the bleeding liver, showing a wound adhering and hemostatic effect.

**Supplementary Movie 4:** *In vivo* adhesion application on pig liver using SUP glue (K72-SDBS) to show its tissue adhering and hemostatic properties. A pig model was employed for the test. A bleeding wound on the liver was produced by a needle. The pig was 2-3 months old and 20 kg of weight.

**Supplementary Movie 5:** *In vivo* control test on pig liver using commercial adhesive (Histoacryl^®^). The major component of Histoacryl is cyanoacrylate. When applying this adhesive on the wounds, rigid plaques appeared and then blocked the wound area because of the formation of polymer chains induced by the water contained in the blood.

**Supplementary Movie 6:** *In vivo* adhesion application of SUP glue (K72-SDBS) on pig kidney to show its tissue adhering and hemostatic properties.

**Supplementary Movie 7:** *In vivo* adhesion application of SUP glue (K72-SDBS) on pig heart to show its tissue adhering and hemostatic properties.

**Supplementary Movie 8:** *In vivo* control experiment on pig heart using a commercial adhesive (Histoacryl^®^).
