# Supplementary material for: Ultra-strong bio-glue from genetically engineered polypeptides
Source: Nat Commun. 2021 Jun 14;12:3613. doi: 10.1038/s41467-021-23117-9 (PMC8203747; doi:10.1038/s41467-021-23117-9)
Supplement: Supplementary file 2 — Peer Review File [file 41467_2021_23117_MOESM2_ESM.pdf]

The previous round of peer review came from another journal.

REVIEWERS' COMMENTS

Reviewer #1 (Remarks to the Author):

In the revised paper reporting polyelectrolyte complexes formed from elastin-like polypeptides (VPGKG)n and anionic surfactant sodium dodecylbenzenesulfonate (SDBS), which can be utilized as a biomedical glue, the authors have sufficiently addressed the major points raised regarding their study and have significantly improved the quality of their work. Considering the corrections made, this work is now suitable for publication in Nature Communications as previously recommended.
